# A Multipotent Precursor Approach for the Preparation of High‐Molecular Weight Conjugated Polymers with Redox Active Units

**DOI:** 10.1002/smtd.202500488

**Published:** 2025-04-30

**Authors:** Benedetta Bertoncini, Andrea Taddeucci, Sabrina Trano, Sofia Raviolo, Ilaria Valdrighi, Federico Maria Vivaldi, Virgilio Mattoli, Federico Bella, Marco Carlotti

**Affiliations:** ^1^ Dipartimento di Chimica e Chimica Industriale University of Pisa Via G. Moruzzi 13 Pisa 56124 Italy; ^2^ Chimie ParisTech PSL University 11 Rue Pierre et Marie Curie Paris F‐75005 France; ^3^ Department of Applied Science and Technology Politecnico di Torino Corso Duca Degli Abruzzi 24 Turin 10129 Italy; ^4^ Center for Materials Interfaces Istituto Italiano di Tecnologia Viale R. Piaggio 34 Pontedera 56025 Italy

**Keywords:** conjugated polymers, organic electronics, polymeric precursors, potassium ion batteries, redox polymers

## Abstract

Conjugated polymers have long been recognized as key materials in organic electronics, yet, in many instances, their processability remains challenging due to their inherent poor solubility and limited polymerization degrees, which limit the scope of several materials in device fabrication. In this study, a multipotent precursor strategy is introduced that enables the synthesis of high‐molecular‐weight conjugated materials incorporating either anthracene or anthraquinone units from a single precursor. These latter, based on 9,10‐dihydroanthracene units, can be polymerized to high polymerization degrees and possess high solubility and processability, thanks to the flexibility of the main chain and the presence of sacrificial side chains. Different post‐polymerization transformations allow the selective generation of conjugated polymers, preserving the polymerization degree and generating, from an identical precursor, different conjugated polymers characterized by a different chemical nature and different electronic characteristics. Remarkably, these transformations can also be performed on the precursors in the solid state without affecting drastically their morphology. Finally, the potential of this methodology is demonstrated in the fabrication of organic field‐effect transistors and organic cathodes for potassium‐ion batteries.

## Graphical abstract

A versatile precursor approach enables the synthesis of high‐molecular‐weight conjugated polymers with tunable electronic properties from a single dihydroanthracene‐based scaffold. Post‐polymerization transformations afford distinct materials incorporating anthracene or anthraquinone units. High solubility, solid‐state processability, and device integration are demonstrated, unveiling exciting opportunities in organic electronics and potassium‐ion batteries.

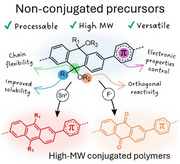

## 1 Introduction

Conjugated polymers (CP), characterized by an extended delocalization of pi electrons, small bandgaps, and accessible electronic states, can be regarded as key materials for the advancement of the technology of our time.^[^ ^1^ ^]^ Thanks to their appealing optoelectronic properties, the possibility of fine tuning their properties via synthetic methods, and availability over their inorganic counterparts, CP have attracted much attention for applications that meet the current societal needs. This spans, just to name a few, from the development of functional electronic devices—such as organic light emitting diodes (OLEDs), field‐effect and electrochemically gated transistors, electrochromic displays and flexible/conformable electronic devices^[^ ^2^ ^]^—to sensing^[^ ^3^ ^]^ and healthcare,^[^ ^4^ ^]^ to fundamental contribution in the energy harvesting and managing, as in the cases of photovoltaic devices (OPV), secondary batteries,^[^ ^5^ ^]^ and supercapacitors.^[^ ^6^ ^]^

Chemistry plays a central and fundamental role in the advancement of CP and related technologies,^[^ ^7^ ^]^ designing innovative and functional molecular structures, developing novel (and scalable) synthetic strategies to achieve high‐molecular weight defect‐free materials, and optimizing their properties and processability. This latter point is of particular importance as many of these materials show poor solubility, because of their flat structure and the possibility of forming extended aggregates guided by favorable π–π interactions.^[^ ^8^ ^]^ The low solubility can limit the degree of polymerization and make the fabrication of devices far more complex (preventing, for instance, the deposition from solution or direct printing), thus rendering chemically interesting materials useless from a practical perspective. The addition of side chains as solubilizing groups can help overcoming this issue and also improve the performances of the materials in several applications, for example, by guiding the packing of the chains in an optimal way or by allowing better compatibility between the polymer and a different phase.^[^ ^9^ ^]^ However, for many materials, the latter approach is not viable as it may also induce a torsion between the units, diminishing the conjugation length, or add unnecessary unresponsive material, thus lowering the performances of the devices in applications that require a high density of functional centers per unit weight (or volume). This is particularly relevant, for example, in the case of batteries, for which one of the strengths of organic electrodes over inorganic ones is the high specific capacity.^[^ ^10^ ^]^

Another way to address the issue of limited processability consists of the use of precursors characterized by better solubility and favorable properties, which can be transformed in the final target material through a quantitative, clean, and selective reaction.^[^ ^11^, ^12^, ^13^ ^]^ Several of such examples have existed for decades in the cases of the more classical CP such as polyacetylene,^[^ ^14^ ^]^ poly(phenylene vinylene),^[^ ^15^ ^]^ and polyphenylene,^[^ ^16^ ^]^ but similar approaches have also been reported for the preparation of more complex CP such as donor–acceptor polymers,^[^ ^17^, ^18^, ^19^ ^]^ ladder polymers,^[^ ^20^ ^]^ and small molecules.^[^ ^21^, ^22^, ^23^, ^24^ ^]^ These methods usually involve the design of a specific precursor that, through a high‐yielding final reaction promoted by heat, chemical agents (such as strong bases or acids), or, more rarely, light, generate a specific target polymer.^[^ ^25^ ^]^

In this study, we report a class of highly processable precursors that, unlike the previous examples, can undergo two different final reactions that can yield two different CP from the same starting material. Because of this characteristic, we call them “multi‐potent.” In particular, these polymeric precursors contain the 9,10‐dihydroanthracene units opportunely functionalized so that, by employing different conditions, it is possible to obtain high‐molecular weight fully conjugated materials comprising either anthracene or anthraquinone units, with quantitative yields and high selectivity. In a recent study, we showed the potential of this approach to prepare multi‐color electrochromic surfaces and unipolar p‐ and n‐type OFETs comprising different conjugated polymers originating from the same non‐conjugated precursor.^[^ ^26^ ^]^

**Figure 1:**
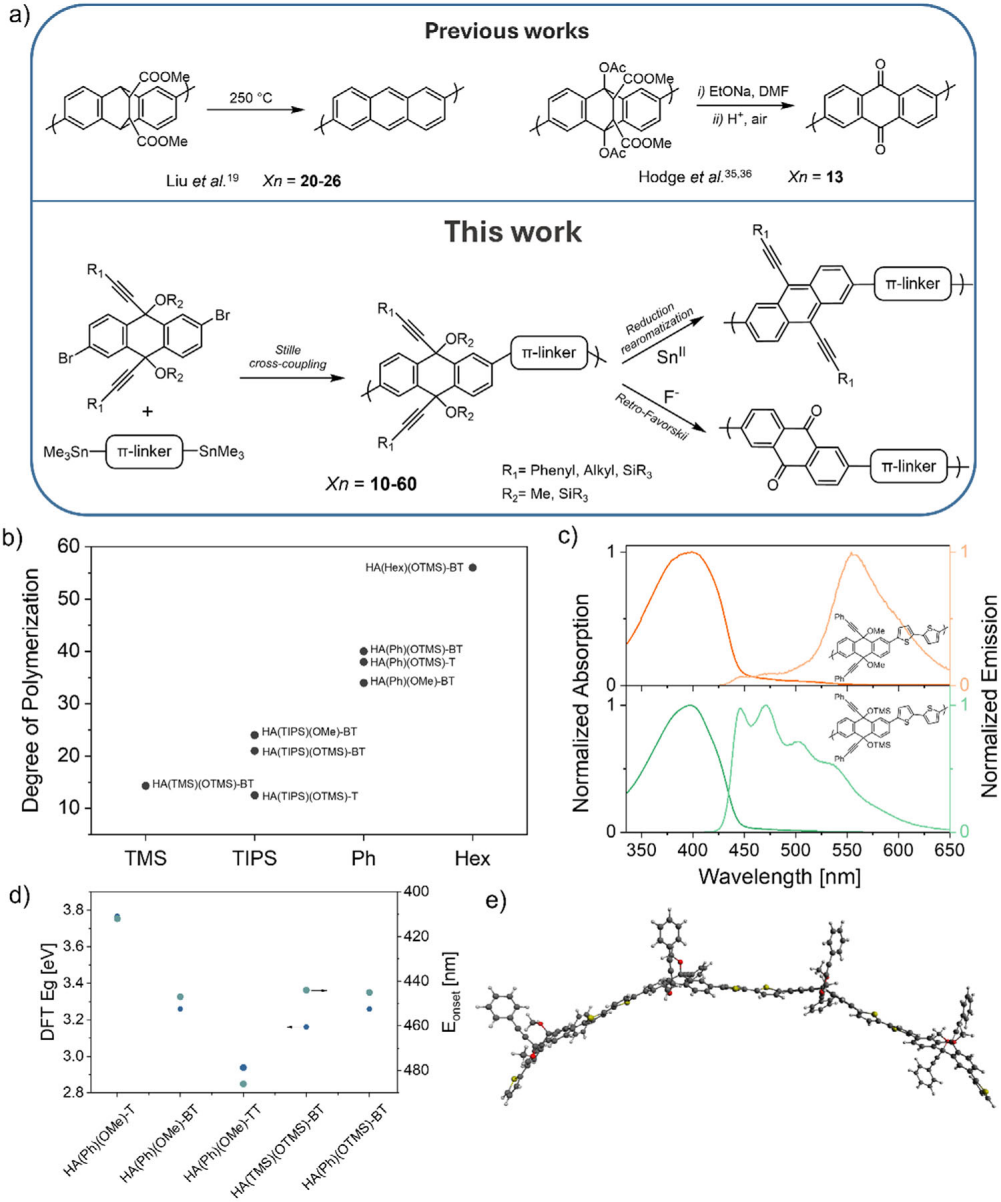
a) Comparison of this work with previous literature describing the preparation of anthracene and anthraquinone containing polymers via precursor routes. b) Correlation between the degree of polymerization (calculated employing Mn) and the substituents on the alkyne. c) Normalized absorption and emission spectra of **HA(Ph)(OMe)‐BT** (orange) and **HA(Ph)(OTMS)‐BT** (green). d) Optical bandgap of selected precursors calculated employing density functional theory (DFT) and measured onset of the absorption spectrum. e) Optimized geometry of a tetramer of **HA(Ph)(OMe)‐BT** highlighting the high‐flexibility of the precursors.

Anthracene‐based materials have found application in diverse fields where a medium bandgap can be useful, such as OPV,^[^ ^27^ ^]^ OLEDs,^[^ ^28^ ^]^ OFETs,^[^ ^29^ ^]^ and sensing.^[^ ^30^ ^]^ Materials comprising the anthraquinone moiety, on the other hand, are known for their reliable redox properties and chemical stability, and are often used in dye‐sensitized solar cells,^[^ ^31^ ^]^ charge storage devices,^[^ ^10^ ^]^ catalysis,^[^ ^32^ ^]^ sensing,^[^ ^33^ ^]^ and in antimicrobial coatings.^[^ ^34^ ^]^ Synthetic strategies for the preparation of polymers containing either anthracene^[^ ^19^ ^]^ or anthraquinone^[^ ^35^, ^36^ ^]^ in chain units based on precursor methodologies have been proposed in the literature. These latter rely on the use of Diels–Alder adducts to induce torsions and introduce steric hindrance to prevent aggregation (as summarized in **Figure** **1**). However, the obtained degrees of polymerization were not very high (especially in the case of anthraquinone polymers) and the procedures for their transformation in the fully conjugated polymers relied on high temperatures and harsh conditions, thus preventing their use in common fabrication procedures and their incorporation in functional devices.

Contrarily to this approach, the methodology we are proposing not only allows to reach large polymerization degrees and employs mild conditions for the final transformation, but also offers the opportunity to prepare both the anthracene and the anthraquinone containing polymers from the same precursor. Remarkably, one can perform these different transformations in solution or in the solid state (e.g., on a thin film), thus allowing as well the preparation of single organic layers with different chemical and electronic characteristics. Following the approach described herein, one can obtain from the same precursors different target CP characterized by complementary electronic properties, the performances of which can be additionally tuned by employing different linkers. Finally, we show the versatility of this approach in the preparation of OFETs and energy storage devices.

## 2 Result and Discussion

### 2.1 Synthesis and Characterization of the Precursor Polymers

To obtain multipotent precursors, as shown in Figure 1, we investigated polymers comprising a 9,10‐dihydroanthracene (**HA**) units, functionalized with substituted propargyl alcohol moieties in the 9 and 10 positions, and a linker. These materials could be obtained employing standard Pd‐catalyzed cross‐coupling polymerization conditions, starting from the 2,6‐dibromo derivative of the dihydroanthracene unit, and resulting in high degrees of polymerization, usually comprising between 10 and 60 units (**Table** **1**). The presence of the propargyl alcohol moiety not only breaks the conjugation by inserting a more flexible sp^3^ carbon, but also offers several functionalization opportunities (on the triple bond and/or on the hydroxyl group) to insert solubilizing chains or particular functionalities, resulting in different options to improve the solubility and the overall processability of the precursors and/or imparting a specific reactivity. A list of the main **AH**‐containing polymeric precursors obtained via Stille polymerization and employed in this study is summarized in Table 1, together with their characteristics. They were characterized by nuclear magnetic resonance (NMR), gas permeation chromatography (GPC), infrared (IR) spectroscopy, and elemental analysis (see Supporting Information). More precursor polymers and more characterizations are reported in the Supporting Information. As it will be described more in detail in the following sections, the **HA** groups can be transformed, with fast and high‐yielding reactions, to either 9,10‐diethynyl‐anthracene (**AC**) or anthraquinone (**AQ**) units without affecting the overall degree of polymerization, thus generating fully conjugated polymers, the direct synthesis of which would only generate oligomers (Table 1 bottom).

**Table 1:** Summary of the characteristics of the precursor polymers discussed in the study. More polymers can be found in the Supporting Information.

Polymer	R1	R2	Linker	Yield^a)^ [%]	Mn	Mw	PDI	Xn^b)^
HA(Ph)(OMe)‐T	Phenyl	Methoxy	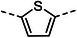	85	15 400	64 100	4.2	30
HA(Ph)(OTMS)‐T	Phenyl	TMS	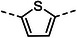	83	23 900	128 700	5.4	38
HA(Ph)(OMe)‐BT	Phenyl	Methoxy	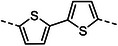	87	20 600	9200	4.5	34
HA(Ph)(OTMS)‐BT	Phenyl	TMS	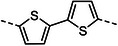	83	29 300	130 900	4.5	40
HA(Ph)(OMe)‐TT	Phenyl	Methoxy	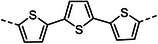	73	9700	26 300	2.7	14
HA(Ph)(OTMS)‐TT	Phenyl	TMS	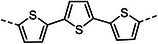	67	17 100	73 600	4.3	21
HA(TIPS)(OTMS)‐T	TIPS^c)^	TMS	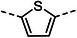	72	9900	16 700	1.7	13
HA(TIPS)(OMe)‐BT	TIPS	Methoxy	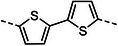	85	18 200	34 700	1.9	24
HA(TIPS)(OTMS)‐BT	TIPS	TMS	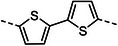	77	18 600	42 900	2.3	21
HA(TIPS)(OTMS)‐Tz	TIPS	TMS	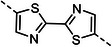	74	11 600	19 900	1.7	13
HA(Ph)(OTMS)‐Tz	Phenyl	TMS	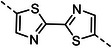	71	9500	52 700	5.6	13
HA(Hex)(OTMS)‐BT	Hexyl	TMS	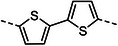	73	34 800	135 000	3.9	56
HA(Hex)(OTMS)‐T	Hexyl	TMS	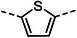	82	17 600	57 300	3.2	27
HA(TMS)(OTMS)‐BT	TMS	TMS	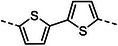	81	10 200	17 700	4.0	14
*Direct polymerization*								
AC(Ph)‐T	–	–	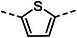	<1%	600	800	1.3	1.4
AC(TIPS)‐BT	–	–	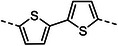	<1%	1300	1600	1.2	1.9
AQ‐T	–	–	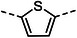	<1%	600	800	1.4	1.9
AQ‐BT	–	–	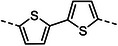	<1%	700	500	1.4	1.5

^a)^ Chloroform fraction;

^b)^ Calculated by dividing the Mn obtained via GPC by the molecular weight of the repeating unit;

^c)^ Triisopropyl silyl.

The synthesis of the **HA** monomers is quite straightforward and can be obtained by nucleophilic attack on 2,6‐anthaquinone of an acetylide formed in situ. The alkoxide that forms can be protonated during workup, alkylated or protected with silicon protecting groups, such as tetramethylsilane (TMS). This approach offers a high degree of freedom on the groups that can be inserted on the **HA** unit so that the precursor can meet the desired requirements in terms of chain length, reactivity, processability, and compatibility with specific substrates. As these groups are lost during the transformation, they can be regarded as expendable solubilizing chains that increase the solubility of the precursor, but not that of the final polymers.

For example, it is possible to observe from the data reported in Table 1 and visualized in Figure 1a that the group present on the alkyne moiety has a prominent effect on the molecular weight of the obtained polymer. In particular, from our results, the solubility imparted from such side chain seems to correlate positively with the degree of polymerization, with the use of TMS groups resulting, on average, in the lower polymerization degree and the hexyl chains allowing the insertion of the largest number of units. Other parameters—such as the catalyst employed, the functionalization on the oxygen, or the linker—seem to correlate less with the degree of polymerization (see Supporting Information).

These latter, however, affect other characteristics of these systems. For instance, we observed that the steric hindrance of the groups present on the oxygen atom of the propargyl alcohol prevents the formation of aggregates in solution and produces intense fluorescence emission even in the solid state. A notable example of this can be observed from the absorption and fluorescence spectra of solutions of **HA(Ph)(OMe)‐BT** and **HA(Ph)(OTMS)‐BT**, two polymers that only differ for the different substitutions on the hydroxyl moiety. As one can observe from Figure 1c, while the absorption maxima are very similar (398 and 399 nm, respectively; see Figure S3, Supporting Information), the peak for **HA(Ph)(OMe)‐BT** is broader and characterized by a red‐shifted tail which we ascribed to an indication of aggregation of the polymeric chains.^[^ ^37^ ^]^ The fluorescence spectra and DLS analysis (which evidenced the presence of particles with a solvodynamic radius in chloroform of about 190 nm) are consistent with this observation. In particular, the emission of **HA(Ph)(OTMS)‐BT** is characterized by a Stokes shift of 47 nm and a profile that suggests a band structure; these signals, notably, are present with very low intensity in the case of the methoxy analogue, the emission of which is dominated by a feature‐less emission centered at 556 nm (a Stokes shift of 157 nm), suggesting the formation of fluorescent small‐sized aggregates even at dilute concentrations.^[^ ^37^ ^]^ Similar results are obtained for polymers comprising different linkers and units (see Figure S4, Supporting Information). However, we cannot exclude that these features are due to the presence of in‐chain defects, which have been shown to induce similar behaviors in several systems.^[^ ^38^, ^39^ ^]^ A better understanding of the optical properties of these systems is currently under investigation.

Conversely, also the choice of the linker seems to affect the degree of polymerization marginally—albeit linkers characterized by long and flat geometries (such as terthiophene, **TT**) or that promote planarization (as in the case of bisthiazole, **Tz**) seems to yield lower molecular weights (see Table 1). The linker, by affecting the conjugation length, affects the optical properties of the precursor **AH** polymers, as shown in Figure 1d, and, as it will be discussed in a later section, also of the redox properties of the final donor–acceptor conjugated polymers.

Besides the role of the sacrificial side chains, the flexibility of the main chain, that originates from the presence of the sp^3^ carbon atoms in the **HA** units,^[^ ^40^ ^]^ plays an even more fundamental role in the preparation of high‐molecular weight, highly processable precursors. DFT‐optimized geometries of model tetramers (employing B3LYP/6‐31g(d) basis set and GD3BJ dispersion correction) were indeed found to be bent structures with the side groups protruding preferably on the same molecular plane (as shown in Figure 1e) or on alternated sides (see Figures S5–S7, Supporting Information). While the former geometry is preferred by polymers comprising linkers characterized by an even number of rings and the latter is preferred by those materials containing an odd number of rings in the linker, the differences in single point energies are generally non‐significant at room temperature (see Supporting Information). These observations are meant to be qualitative, to support the fact that the chains can rapidly assume different conformations thanks to their flexibility, rather than actually describing the states and energies of the polymer chains. To support these observations, when in place of the flexible **HA** unit we employed a fluorene derivative with a similar functionalization on the 9 position (see Supporting Information), the polymerization rapidly yielded an insoluble material for which the GPC analysis of the chloroform fraction evidenced a low polymerization degree (Xn = 5.4). This difference in behavior during polymerization can be ascribed to the flatness and rigidity of the core in the case of the latter, which, despite the presence of similar side chains, still maintains a full conjugation and make the precursor prone to aggregation and precipitation (see Supporting Information). As shown in this latter example, it is worth mentioning that the same strategy could also be employed for the synthesis of other copolymers comprising solubilizing units that are not based on the anthracene three‐ring skeleton. In particular, to show this possibility we prepared precursors comprising units derived from benzophenone and benzoquinone, which resulted in polymers with Xn of 28 and 25 units, respectively (see Supporting Information).

### 2.2 Precursors Transformation to the Conjugated Materials

As mentioned before, a peculiar trait of the multipotent precursors we are presenting here is the fact that they can undergo different transformations to generate two different conjugated polymers, which are characterized by different chemical structures, but that retain the degree of polymerization (Figure 1). As a result, it is possible to obtain conjugated polymers with a much higher molecular weight compared to what it would be possible to obtain by direct polymerization of the same units. Notably, the final polymers, which either comprise anthracene or anthraquinone units, have different redox properties and complementary transport properties as recently shown.^[^ ^26^ ^]^ Upon generating a fully conjugated backbone, the materials become highly insoluble due to the strong aggregation between chains.^[^ ^36^ ^]^

To transform the **HA** units into 9,10‐diethynylantrhacene units (**AC**) one can employ a reduction‐rearomatization reaction.^[^ ^41^, ^42^, ^43^ ^]^ Conversely, a retro‐Favorskii reaction on the same units yields anthraquinone (**AQ**) centers. It is worth mentioning that the direct polymerization of the same **AQ** or **AC** units with the linkers described in this manuscript, in the same reaction conditions, only yields insoluble materials even after 30 min. Extraction with boiling chloroform yielded only a small amount of material consisting in extremely short chains (see Table 1).

The reduction‐rearomatization reaction commonly employs Sn^II^ ions in an acidic environment to eliminate the –OR_2_ group and establish the aromaticity within the central ring of the molecule.^[^ ^43^, ^44^ ^]^ While this is not the only way to perform this reaction, we were able to optimize a variation such protocol so that the transformation of **HA** to **AC** was quantitative, selective, and rapid.^[^ ^26^ ^]^ In particular, these reactions were carried employing SnCl_2_ in a solution of methanol containing 1% of concentrated HCl at 60 °C. The reaction is accompanied by a rapid change in color (to dark red). The efficacy of this methodology was reported and investigated profusely in a previous study.^[^ ^26^ ^]^ In particular, in most cases for **AC** polymers, the ratio between the carbon and sulfur content obtained from elemental analysis was found to be in good agreement with what expected from the molecular formulas (within 5% from the expected value; see Table S2, Supporting Information). The deviations are due to the possible presence of precursor units that did not react and to byproducts of the reaction. For instance, the reduction‐rearomatization reaction can leave behind SnO_2_ residues which are not easily washed away. XPS measurements indeed evidenced the presence of traces of Sn in the samples (Table S3 and Figure S15, Supporting Information). The role of XPS and elemental analysis in describing the role of impurities in these systems are discussed in details elsewhere.^[^ ^26^ ^]^

**Figure 2:**
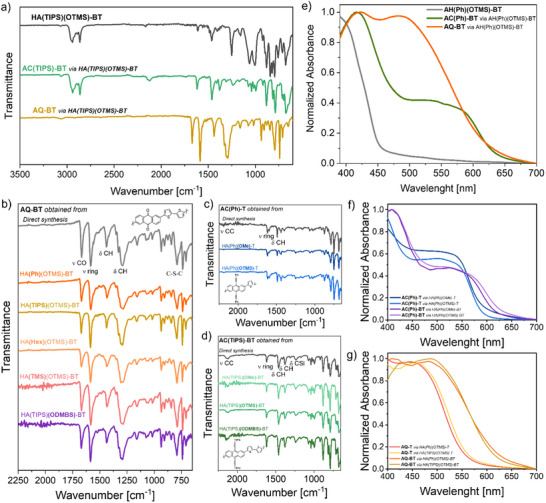
a) FT‐IR spectra of precursor polymer **HA(TIPS)(OTMS)‐BT** (gray) and the conjugated polymers **AC(TIPS)‐BT** (light green) and AQ‐BT (ocher) obtained from the former. b) Confrontation of the FT‐IR spectral profiles of AQ‐BT obtained from direct polymerization of anthraquinone and bithiophene (gray) and from different precursors (as specified in the image). c,d) Similar plots concerning the conjugated polymers **AC(Ph)‐T and AC(TIPS)‐BT**. e) Absorption spectra of thin films of **HA(Ph)(OTMS)‐BT** (gray) and the conjugated polymers **AC(Ph)‐BT** (green) and **AQ‐BT** (orange) derived from the former. Confrontation of absorption profiles of f) **AC** and g) **AQ** polymers with identical structures obtained from different precursors.

In **Figure** **2**a, we reported the IR spectra of **HA(TIPS)(OTMS)‐BT** as prepared and after both the reduction‐rearomatization and the retro‐Favorskii (which will be discussed more in details below) reactions. Similar plots for the rest of the polymers can be found in the Supporting Information (Figures S10 and S11, Supporting Information). By comparison of the profiles of the precursor and the related **AC** polymer (in this case, **AC(TIPS)‐BT**), it is possible to observe the loss of the signal relative to the OTMS group, which is characterized by a cluster of signals in the 1200–1000 cm^−1^ (related to the C─O─Si bonds). Remarkably, as shown in Figure 2c, the resulting spectrum is identical to that of **AC(TIPS)‐BT** obtained by the direct polymerization of the linker and the respective **AC** unit or the same conjugated polymer generated from **HA(TIPS)(OMe)‐BT** employing identical conditions. This fact suggests that it is possible to employ different precursors to yield the same final polymer, thus allowing more flexibility in the synthetic process and in the processability of the precursor.

Remarkably, by employing a methanolic solution, which does not dissolve the polymer, the transformation can also be performed on a solid film of **HA** precursor, yielding a film of **AC** polymer without redissolving it. In this sense, this approach can be used as an immobilization strategy to produce organic layers that cannot be easily redissolved and thus it can help the design of more accessible multi‐step fabrication strategies for complex devices, which are often limited by orthogonality issues. Thanks to this feature, we were able to acquire the absorption spectra of spin coated films of various **AC**‐polymers (Figure 2f). It is worth mentioning that films of these materials would not be otherwise obtainable if not through the precursor route because of their intrinsic low processability.

Precursors films are faintly colored, with absorption maxima found in a range that goes from about 360 nm (for the ones bearing a single thiophene ring as inker) to about 432 nm (for those characterized by the longer terthiophene linker), further red shifted from that of the precursors. These results qualitatively agree with DFT calculations which predicted a shorter bandgap as the length of the conjugated spacers between the **HA** units increases. As we show in Figure 2e–g, upon reduction‐rearomatization the absorption profile shifts to longer wavelengths, in accordance with the lowering of the bandgap as a result of the extension of the conjugation path along the main chain. The absorption spectra of films prepared with the rest of the polymers listed in Table 1 are reported in Figure S4 (Supporting Information).

The reduction of the bandgap depends on both the linker, with longer conjugated linkers shortening the bandgap more effectively, and the side group connected to the ethynyl groups, which influence the electronic characteristics of the polymer. In Figure 2f, for example, we show the absorption spectra of films of **AC(Ph)‐T** and **AC(Ph)‐BT** originating from **HA(TIPS)(OMe)‐T** and **HA(TIPS)(OTMS)‐T**, **HA(TIPS)(OMe)‐BT**, and **HA(TIPS)(OTMS)‐BT**, respectively. The plots indeed suggest that the extension of the linker from one thiophene ring to two shifts the absorption onset of about 30 nm (0.10 eV). This evidence is supported by DFT calculations, as shown in the Supporting Information (Figures S8 and S9, Supporting Information). In addition, it is possible to observe that identical polymers are characterized by similar optical properties even when originating from different precursors. Deviations of perfectly identical behaviors are due to experimental artifacts related to the possible formation of different aggregated states and optical aberrations arising from the interference in thin films.

The transformation of **HA** units in **AQ** ones, as mentioned earlier, uses the retro‐Favorskii reaction.^[^ ^45^ ^]^ This latter employs a strong base and high temperatures to remove the alkyne fragment from a propargyl alcohol and form a carbonyl group. Such strategy often finds use in synthetic protocols that require the protection of a terminal alkyne. Conventional procedures for this reaction usually entail treating precursors with a base such as KOH, at high temperatures (>100 °C) for several hours.^[^ ^46^, ^47^ ^]^ Notably, we found that the same reaction would occur when tetrabutylammonium fluoride (TBAF) was employed, but at a lower temperature (70 °C), for much shorter time (full conversion within minutes), and in a broad range of solvents.^[^ ^48^, ^49^ ^]^ This is because the tendency of the fluoride ion to behave like a strong base in absence of water, thus allowing the retro‐Favorskii to proceed.^[^ ^50^ ^]^ Simultaneously, fluoride ions can also efficiently and rapidly remove the silicon protecting group the propargyl alcoxide, thus allowing to use this approach for the precursors polymers reported in Table 1 bearing the TMS group as if they were free alcohols. As the case above, this transformation method was investigated in depth in previous studies.^[^ ^26^ ^]^ The resulting **AQ**‐polymers are characterized by a relatively low‐energy LUMO level and a cross‐conjugation pattern.

An example of an IR spectrum of a polymer comprising **AQ** units connected in positions 2 and 6 by a bithiophene (**AQ‐BT**) is reported in Figure 2a together with the one obtained from the precursor used to prepare it, **AH(TIPS)(OTMS)‐BT**. Similar data for the rest of the precursor polymers listed in Table 1 are reported in Figures S10 and S11 (Supporting Information). The formation of the quinone is evident from the double band at 1660 and 1580 cm^−1^ (characteristic of systems comprising anthraquinone) and the absence of the signals ascribable to the C─O─Si bond (1200–1000 cm^−1^), the triple bond (2100 cm^−1^), and aliphatic methyls and methylenes (2800–3000 cm^−1^). Moreover, the profile is identical to that of the same **AQ**‐containing polymer obtained via the direct polymerization of 2,6‐dibromoanthraquinone and 2,5‐bis(trimethylstannyl)bithiophene (Figure 2b). Elemental analysis of the **AQ** polymers evidenced a good agreement with regards to the expected C/S ratio (within 7%; see Table S2, Supporting Information), although, as already discussed in the case of the AC counterparts, parts of unreacted precursor could still be present. The elemental analysis also evidenced the presence of traces of nitrogen in the sample (>0.15%), suggesting the presence of possible byproducts arising from the decomposition of the tetrabutylammonium. These, however, were not observed in the XPS (nor was fluorine). Such discrepancy could indicate that these contaminants are not bound to the chains and are removed in the high‐vacuum required by this technique.

As the retro‐Favorskii reaction eliminates both the alkyne and the group linked to the oxygen, the **AQ** polymers obtained from different **HA** precursors comprising the same linker will be chemically identical. This is shown in Figure 2, where both the IR spectra and the UV–vis absorption of these materials are compared. Such evidence suggests that it is possible to use different **HA** precursors bearing several groups on the alkyne to prepare polymers characterized by a different number of units in their chains and different chemical characteristics, from which the same **AQ**‐containing polymer structure can be easily obtained. In other words, one can use the precursor that better fits their needs and, after the transformation, obtain polymers which are chemically identical. A similar reasoning was already made above for **AC** polymers. This highlights the freedom offered by the multipotent precursor approach described herein, in which one can design a precursor that not only can generate two different target materials, but also prepare different precursors to obtain the same final polymer.

As it was for the Sn^II^ promoted reduction‐rearomatization reaction in methanol, also in the case of this transformation from **HA** to **AQ** one can employ a solvent that does not dissolve the precursor (e.g., acetone, methyl‐isobutyl‐ketone, hexane) so that the retro‐Favorskii reaction can be carried out on films and fine powders without dissolving them. The effects of the transformation on the optical properties of the films are summarized in Figure 2d,f, and in Figure S13 (Supporting Information). The formation of fully conjugated backbones shortens the optical band gap in the case of both **AC** and **AQ** polymers. As also suggested by the IR results, films of identical polymers originated from different precursors —e.g., AQ polymers comprising the same linker, AC polymers comprising the same linker and side functionalization—were also characterized by a comparable absorption onset (Figure 2e–g).

Unlike the reduction‐rearomatization reaction, which can be applied successfully to all the precursors listed in Table 1, the transformation described to obtain **AQ** does not proceed in the case of polymers carrying the methoxy group on the propargyl alcohol, as this latter does not degrade in a basic environment, thereby preventing the conversion from **HA** to **AQ**. As a result, the treatment with TBAF has no effect on the chemical nature of precursors bearing the OMe group (see Supporting Information). Evidence of this can also be seen in Figure 2e where one can observe no large variation in the optical properties of a film of **HA(Ph)(OMe)‐BT** that underwent the retro‐Favorskii transformation protocol, unlike its TMS counterpart reported in Figure 2d (small differences in the absorption profile are due to a slight degradation of the precursor film that, unlike the final conjugated polymers, has a non‐negligible solubility).

**Figure 3:**
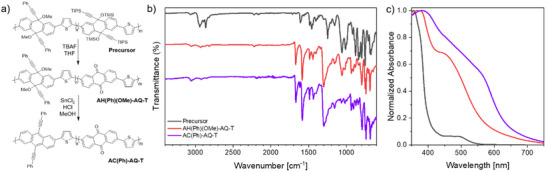
a) Reaction scheme describing the strategy for the preparation of **AC(Ph)‐AQ‐T** random copolymer. b) FT‐IR spectra of the starting precursor polymer (gray), the polymer obtained after the retro‐Favorskii reaction (red), and the final **AC(Ph)‐AQ‐T** random copolymer (violet). c) Normalized absorption spectra of thin films of the same polymers.

This difference in reactivity toward the retro‐Favorskii, but not the reduction‐rearomatization, allows the design of orthogonal fabrication and synthetic strategies in which copolymers carrying both **AC** and **AQ** units can be obtained. A straightforward example of this approach can be found in preparation of a random **AC**‐**AQ** copolymer (**AC(Ph)‐AQ‐T**) employing the precursor copolymer depicted in **Figure** **3**, which comprises **HA**‐units carrying either OTMS or OMe functionalities (Figure S12, Supporting Information). By treating it first using the retro‐Favorskii conditions, one can generate the **AQ** centers only from those units characterized by the presence of TMS groups; a subsequent reduction‐rearomatization step can thus be used to introduce **AC** units only where the **HA** units carrying the OMe group were located, finally generating a polymer characterized by the presence of both **AC** and **AQ** units. This material has a lower bandgap compared to its **AC** or **AQ** analogue, in accordance with DFT calculation performed on tetramers (see Supporting Information). If the reduction‐rearomatization is performed before the retro‐Favorskii, then an all‐**AC** polymer is obtained.

### 2.3 Characterization of Electrochemical Properties

**Figure 4:**
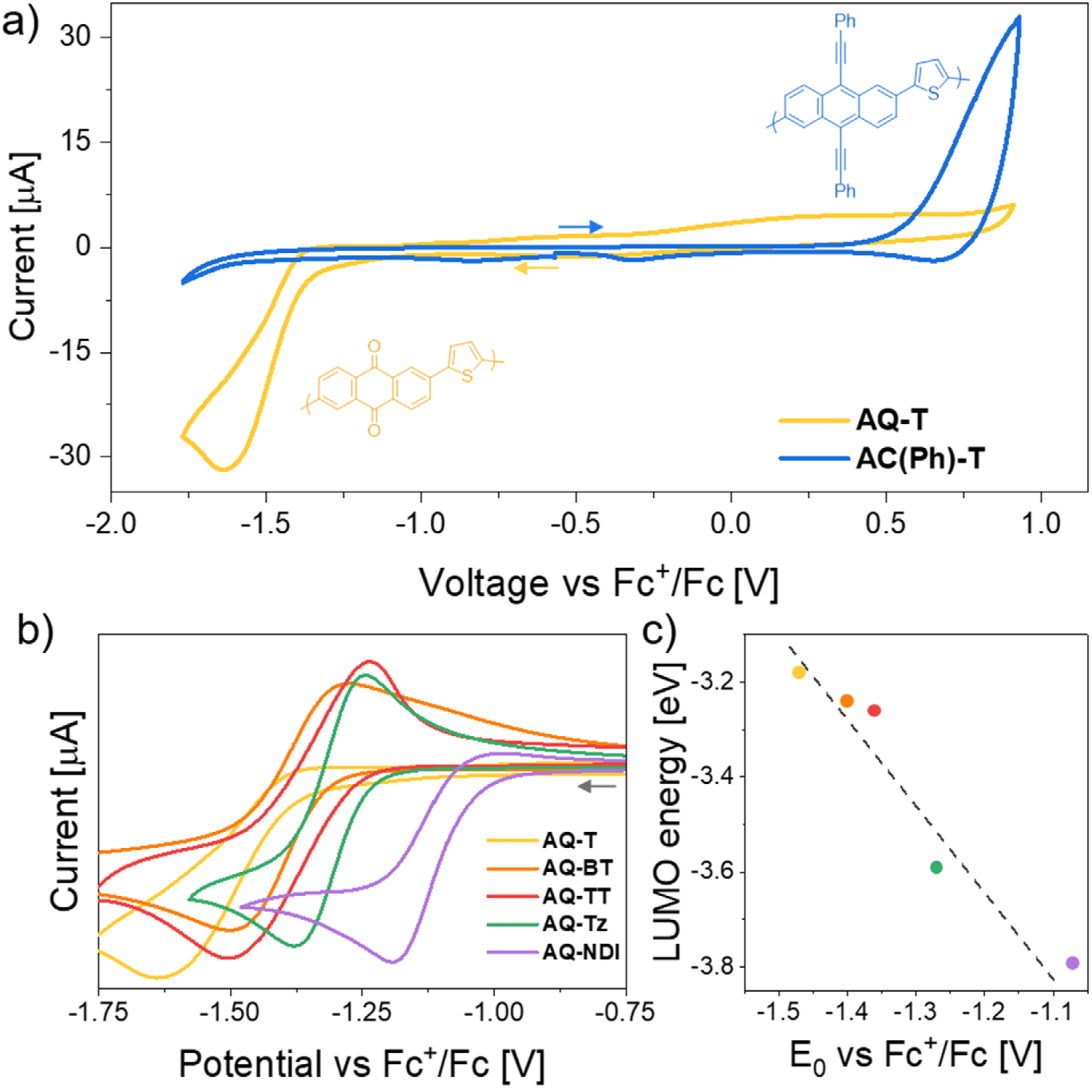
a) Comparison of cyclic voltammograms of thin films of **AQ‐T** and **AC(Ph)‐T** on Au electrodes in 0.1 m tetrabutylammonium hexafluorophosphate in acetonitrile (the arrows represent the starting and direction of the scan for the different materials). b) Reduction peak of thin films of the different anthraquinone polymers prepared in this study. c) Correlation between the LUMO energy obtained via DFT on model tetramers of the same anthraquinone polymers and the position of the reduction peak (the dotted line is meant to guide the eyes).

To evaluate the electronic and electrochemical characteristics of the conjugated materials obtained from the described precursors, we performed cyclic voltammetry on films of both **AC** and **AQ** conjugated polymers prepared via the abovementioned transformations. As mentioned earlier, the possibility of employing these reactions on the films in the solid state allowed us to prepare thin films of the conjugated polymers directly on Au electrodes despite their low processability. Examples of the curves obtained from these materials are shown in **Figure** **4** for the conjugated polymers obtained from **HA(Ph)(OTMS)‐T**. Similar analysis for the other materials are reported in the Supporting Information.

The most striking difference between **AC(Ph)‐T** and **AQ‐T** obtained from the same precursors is that the former presents an evident oxidation peak that the latter does not, while the opposite is true for the reduction peaks. No peaks are observed in the case of the precursor, both in reduction and oxidation. Remarkably, this result supports the generation, from an inert precursor, of redox active species that are characterized by complementary properties, i.e., **AC** is easier to oxidize and **AQ** is easier to reduce. This is in line with what expected from the theoretical calculations, which predicted an increase in the energy of the HOMO in case of **AC** polymers and a more pronounced reduction in LUMO energy in the case of **AQ** polymers. The different HOMO and LUMO energy levels for the different conjugated materials are reported in Figure 4b and in the Supporting Information.

As already reported by us^[^ ^26^ ^]^ and other groups,^[^ ^51^, ^52^ ^]^ the oxidation of 9,10‐substituted anthracenes is generally not fully reversible, and upon cycling it is possible to observe a lowering of the current and, often, the appearance of signals also in reduction (Figure S17, Supporting Information). This complicates the analysis of these features. On the contrary, the reduction of **AQ** containing polymers, characterized by the presence of two peaks in acetonitrile, is highly reversible and reproducible (see Figure S15, Supporting Information). This is common for materials comprising quinones.^[^ ^53^ ^]^ Notably, by changing the linker, it is possible to shift the reduction potential across a range of about 0.5 V (Figure 4c). For example, E_1/2_ was measured to be about −1.47 V versus Fc^+^/Fc for the **AQ‐T** polymer, while such parameter was found to be about 1.31 V when the linker was changed to the more electrowithdrawing dithiazole (**AQ‐Thia**). Insertion of naphtalenebisimide unit in the linker, which does not show redox activity in acetonitrile when teatrabutylammonium hexafluorophospate is used as electrolyte, lowers even further the E_1/2_ to 1.07 V (**AQ‐NDI**). These values follow generally well the trend predicted via DFT, as shown in Figure 4c. These observations support the hypothesis that the proposed precursor strategy can be very versatile for the preparation of high‐molecular weight redox polymers characterized by different electronic characteristics which can be easily tuned by accurate choice of the linker.

### 2.4 Example of Uses of the Precursors in the Fabrication of Devices

One of the peculiar opportunities offered by the precursors we described here consists in the possibility of employing different side groups on the precursors to affect the compatibility between different materials. This has relevant implications for solution‐based fabrication processes for which a good compatibility between the target material and the substrate can help obtaining uniform layers and retaining the desired patterns. To show an example of this approach, we tested the possibility of employing polymers containing **AC** units, obtained from the abovementioned precursors, in bottom‐contacts bottom‐gate p‐type organic field‐effect transistor (OFET) devices. OFETs are a class of transistors that employs, as semiconducting layer, an organic material in which the density of charge carriers can be modulated by applying an electric field through the gate electrode. For their inherent lightweight, flexibility, and the possibility of being prepared via solution processes, they are among the most studied devices in organic electronics. In this context, anthracene‐containing polymers have been reported to show p‐type semiconducting characteristics, albeit with limited mobilities. This was also shown in a previous work from our group in which we fabricated both unipolar p‐ and n‐type OFETs employing the same precursor and an identical geometry.^[^ ^26^ ^]^

In particular, the devices were prepared by spin coating on the devices of ortodichlorobenzene solutions of either **HA(Ph)(OMe)‐BT** or **HA(Ph)(OTMS)‐BT** precursors and performing the subsequent transformation to the conjugated semiconductors by immersion of the substrate in a Sn^II^ methanolic solution, as described above and more in details in the Supporting Information. We chose these two different precursors as, while they yield the same conjugated **AC(Ph)‐BT** polymer (i.e., bearing identical phenyl side groups), they present different aggregation properties in solution (see Figure S3, Supporting Information) and different wetting properties (i.e., the precursor characterized by the methoxy functionality could hardly wet the entire surface).

The obtained mobilities in the linear (*µ*_lin_) and the saturation (*µ*_sat_) regimes were found to be 2.2 ± 0.4 × 10^−2^ and 4 ± 1 × 10^−2^ cm^2^ V^−1^ s^−1^, respectively, for the conjugated polymer **AC(Ph)‐T** originated form the precursor comprising the OTMS group, and 9.6 ± 0.5 × 10^−5^ and 3.9 ± 0.8 × 10^−4^ cm^2^ V^−1^ s^−1^ for the one obtained from the methoxy equivalent, two order of magnitude lower. Such values are lower than the state‐of‐the‐art of p‐type materials, but, nonetheless, in line with previous reports.^[^ ^54^ ^]^ It is worth mentioning that we did not perform any optimization of the device fabrication to improve the charge injection as we believe this is outside the scope of this study. We obtained similar results for polymers in which a TIPS group was employed in place of the phenyl (Figures S17 and S18, Supporting Information). In this latter case, the mobilities were found to be lower—for the **AC(TIPS)‐BT** polymer originating from the OTMS precursor *µ*_lin_ = 2.9 ± 0.8 × 10^−3^ cm^2^ V^−1^ s^−1^ and *µ*_sat_ = 1.3 ± 0.6 × 10^−3^ cm^2^ V^−1^ s^−1^; for the same material obtained from the OMe precursor, *µ*_lin_ = 1.3 ± 0.4 × 10^−4^ cm^2^ V^−1^ s^−1^ and *µ*_sat_ = 3 ± 1 × 10^−4^ cm^2^ V^−1^ s^−1^, one order of magnitude lower.

These results highlight the importance of the affinity between the substrate material and semiconductor both during fabrication and during the operation of the device. By changing the nature of the substituents on the precursors, one can find the best compromise in terms of processability of the materials, compatibility with the substrate, and performances of the target devices. At the same time, the extremely low solubility of the final materials compared to that of the precursors can serve as a platform for the design of novel immobilization strategies for semiconducting active materials, thus facilitating the fabrication of complex and integrated devices which require multiple and orthogonal fabrication steps.

The high polymerization degrees that characterize the conjugated materials obtained via the precursor route is what makes them unprocessable and non‐redissolvable after the final transformation. Next to the possible advantages in the fabrication of devices just described, this aspect can also be particularly interesting for those applications in which the functional material is prone to leaching, affecting the performances and the overall lifetime of the devices. Examples of these latter can be found in organic cathodes employed in electrochemical energy storage (i.e., metal‐ion batteries, supercapacitors) or catalysis. Materials containing quinone functionalities, either small molecules or polymeric, often find use in these applications because of their reliable redox properties, chemical stability, and lightweight. While these materials are generally characterized by low solubility in their neutral state, they may become more soluble in polar environments upon charging, thus detaching from the electrode. This is a common phenomenon for organic‐based energy storage devices, which usually make use of electrolyte solutions characterized by high dielectric constants.

The high molecular weight conjugated polymers which can be obtained through the precursor strategy described in this study, however, can limit this outcome thanks to the intrinsically low solubility of longer polymeric chains. In order to test this hypothesis, we prepared electrodes that employed as active material a mixture of conductive carbon and different conjugated polymers bearing in‐chain anthraquinone units, obtained from the direct synthesis of the conjugated materials or via a precursor, and tested them in lab‐scale potassium ion batteries (KIBs). KIBs have recently garnered significant attention owing to the growing global energy demand, limited lithium reserves, and the unique advantages of potassium, including low cost, abundant natural occurrence (2.09% of the Earth crust), a low standard reduction potential (2.936 V vs SHE), and fast diffusion through the electrolyte/electrode interface.^[^ ^55^ ^]^ Consequently, the development of novel materials suitable for this technology is paramount.

To investigate the performances of the synthesized organic materials as KIB cathodes, we employed an **AQ‐T** oligomer (**oAQ‐T**; Xn < 4), obtained from the direct polymerization of 2,6‐dibromoanthraquinone and 2,5‐bis(trimethylstannyl)‐thiophene, and the **AQ‐T** polymer generated from the **HA(Ph)(OTMS)‐T** (**pAQ‐T**), characterized by a Xn of 38. The cathodes were fabricated by mixing the organic material with C65 conductive carbon and polyvinylidene fluoride binder in a 50:40:10 ratio using *N*‐methyl‐2‐pyrrolidone (NMP) as a solvent. The resulting slurry was coated onto an aluminum current collector. The electrochemical performance was evaluated in potassium‐metal cells, using potassium bis(fluorosulfonyl)imide (KFSI) 1 m in dimethoxyethane as electrolyte, Whatman glass fiber as separator, and potassium metal foil as anode. DME solvent was chosen since, among different solvents (Figure S20, Supporting Information), **oAQ‐T** showed null solubility in DME even after 13 days (Figure S22, Supporting Information). Galvanostatic cycling and cyclic voltammetry (CV) were conducted between 1.2 and 3.5 V with a current density of 0.05 A g^−1^ and a scan rate of 0.1 mV s^−1^, respectively.

**Figure 5:**
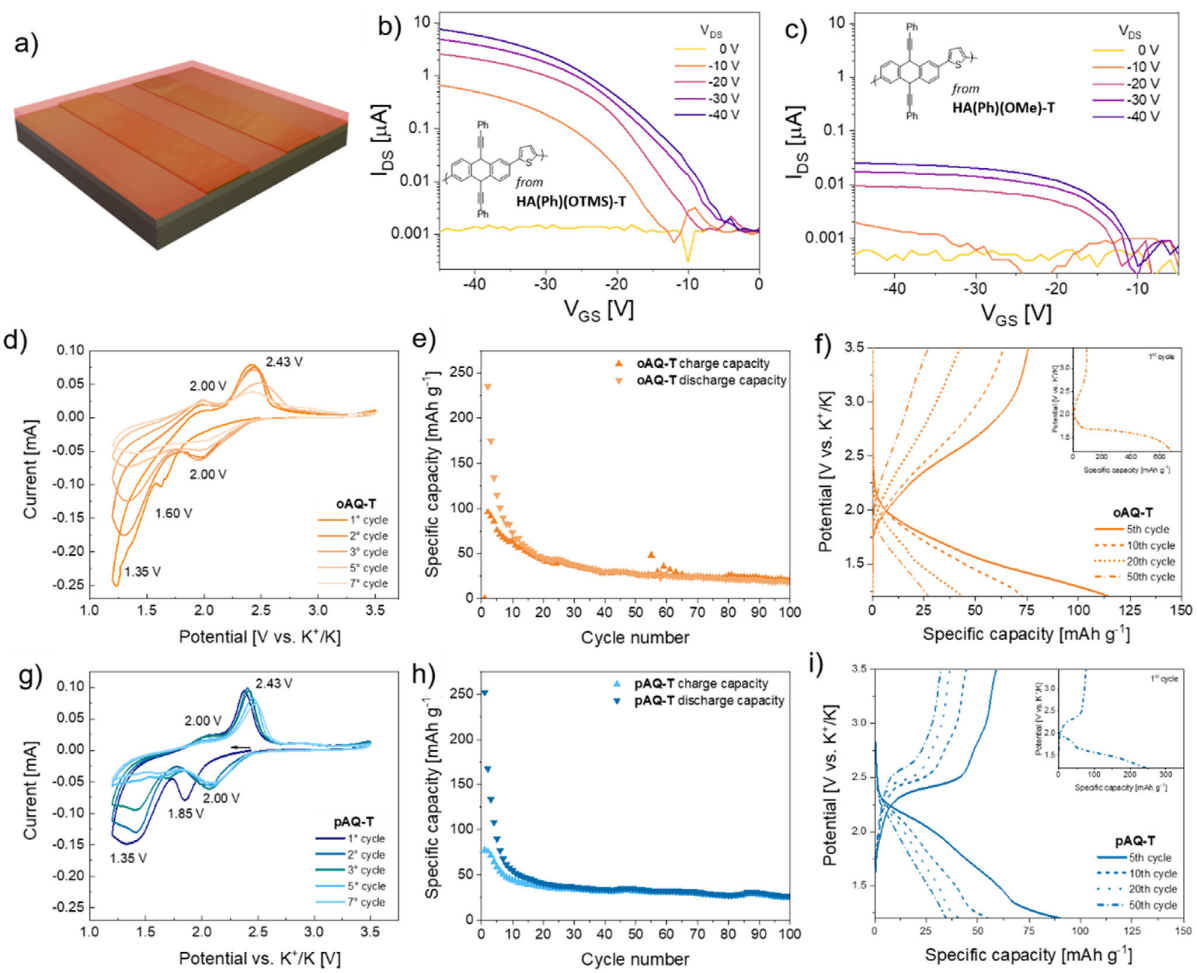
a) Scheme of the design of the bottom‐gate bottom‐contact OFET devices. Transfer curves for devices comprising **AC(Ph)‐T** obtained from b) **HA(Ph)(OTMS)‐T** and c) **HA(Ph)(OMe)‐T**. Comparison of the electrochemical performance of **oAQ‐T** and **pAQ‐T** as cathode active material in KIBs: d,g) cyclic voltammograms; e,h) specific capacity obtained by galvanostatic cycling; f,i) voltage profiles among different cycles.

The redox behavior with two‐electron transfer of **oAQ‐T** and **pAQ‐T** in KIBs, shown in the CV curves reported in **Figure** **5**d, reveals two reduction peaks for the former at ≈2 and 1.35 V (vs K^+^/K), with corresponding oxidation peaks at about 2 and 2.43 V. This suggests a two‐step potassium‐ion storage mechanism through two sequential enolations and K‐ions coordination. The reduction peak at 1.6 and 1.85 V of **oAQ‐T** and **pAQ‐T**, respectively, are both shifted to 2 V during the second cycle of the CV, as it is also possible to observe in the shift of the reduction plateau from the 1st to the 2nd cycle in the potential profiles (Figure 5f,i). The explanation of this shift may relay on the possible rearrangement of the molecules and chains position after the first changing of the oxidation state.^[^ ^56^ ^]^ Both the CV curves and the potential profiles highlight that the irreversible capacity loss in the first cycles is higher in the case of the oligomer. In particular, the CV peaks of **oAQ‐T** are less reversible upon cycling compared to those of **pAQ‐T**. Indeed, while the difference between the discharge and charge capacities of the 1st cycle of **oAQ‐T** is higher than 500 mAh g^−1^, **pAQ‐T** irreversible discharge capacity is around 100 mAh g^−1^. The latter loss is mainly related to decomposition reactions of potassium‐based electrolyte in K‐metal half‐cells,^[^ ^57^ ^]^ while immediate dissolution of **oAQ‐T** into the electrolyte contributes to its higher irreversible discharge capacity (Figure S22, Supporting Information). This decline may stem from changes in the solubility of the organic materials in the electrolyte due to oxidation state variations upon cycling. From the galvanostatic cycling, shown in Figure 5e,h, it can be seen that the initial capacities of both materials are competitive with carbonyl‐based redox cathode materials for potassium batteries,^[^ ^58^, ^59^, ^60^, ^61^ ^]^ but they rapidly degrade after a few cycles. Nonetheless, **pAQ‐T** retained 34% of the 1st cycle specific charge capacity after 100 cycles, against 20% of **oAQ‐T**. Thus, rather than dissolution of the longer chains of **pAQ‐T**, possible agglomeration of the polymeric material during the slurry preparation can be the cause of the improvable specific capacities. Ongoing studies from our research groups aim at further improving the contact and distribution of the polymeric redox active material with the conductive matrix to boost their electrochemical performances.

## 3 Conclusion

In this study we introduced a precursor‐based approach that allows access to high molecular weight conjugated polymers incorporating either anthracene (**AC**) or anthraquinone (**AQ**) units from the same starting material through rapid and selective transformations. In particular, these former rely on the use of 9,10‐dihydroanthracene units functionalized with propargyl alcohol moieties at the 9 and 10 position, which induces structural flexibility in the polymer chain and introduce sacrificial side chain that can be designed to control the reactivity and processability of these materials. Notably, these multipotent precursors can undergo two different post‐polymerization transformations, either a reduction‐rearomatization or a retro‐Favorskii reaction, to generate fully conjugated polymers with different molecular structure and complementary electronic properties, without affecting the polymerization degree. In addition, this precursor strategy also allows for the fine‐tuning of the electronic properties of the conjugated polymers, through the use of different linkers, influencing the redox properties of the final materials.

As the transformation can happen in the solid state, the described strategy allows for the integration of these materials into functional devices while maintaining the structural integrity of the organic layer. As an example of this approach, we prepared OFETs comprising **AC**‐based polymers prepared directly from thin‐films of the precursors. The devices showed decent mobilities, in the order of 10^−2^ cm^2^ V^−1^ s^−1^, without further optimization. In addition, we employed **AQ**‐containing polymers, which demonstrated stable and tunable redox activity, as active material in cathodes for KIB. Compared to the oligomer that one obtains from the direct coupling of the conjugated monomers, the use of high‐molecular weight materials, originating from the longer precursor chains, results in cathodes that have a longer lifetime as a result of the lower solubility of the active material in the polar electrolyte, while, at the same time, allowing an in depth characterization of the characteristics of the material.

Beyond the specific examples reported in this work, this strategy can be extended to the design of new classes of conjugated polymers with tailored optoelectronic characteristics, expanding the scope of organic materials for next‐generation electronic and energy applications.

## 4 Experimental Section

Triisopropylsilylacetylene, trimethylsilylacetylene, trimethylsilylchloride, methyl iodide, phenylacetylene, XPhos Pd G2, 2,5‐bis(trimethylstannyl)thiophene, 5,5′‐bis(trimethylstannyl)‐2,2′‐bithiophene, 5,5″‐bis(trimethylstannyl)‐2,2′:5′,2″‐terthiophene, methyl lithium 1.6 m in diethyl ether, lithium diisopropylamide 1.0 m in THF/hexane, Pd(II) acetate, tetrabutylammonium bromide, *N*,*N*‐diisopropylethylamine, 2‐bromothiazole, hydrochloric acid 37%, *o*‐dichlorobenzene, and tin(II) chloride were purchased from Merck Life Sciences (Italy). 2,6‐dibromoanthraquinone, 1‐octyne, 4‐tertbutyl‐1‐bromobenzene, tetrabutylammoniumfluoride 1 m in THF were purchased from TCI Europe (Belgium). Anhydrous solvents employed in the reactions were obtained from a house system. The synthesis of the monomers and the characterization of the compounds reported in this manuscript can be found in the Supporting Information.

ATR‐FTIR spectra were recorded on a Shimadzu IRAffinit‐1 spectrometer mounting a MIRacle 10 ATR module. NMR (^1^H and proton‐decoupled ^13^C) characterization of the compounds was performed on a Bruker Avance DRX 400 spectrometer at room temperature and employing the chloroform residual peak as internal standard. The spectra were analyzed employing Mestrenova (v14.1.2).

The elemental composition of the polymers was determined via an Elementar Vario Micro Cube for sulfur, nitrogen, carbon, and hydrogen (CHNS).

Gel permeation chromatography (GPC) measurements were performed with an HP1100 Hewlett‐Packard employing polymer solutions in chloroform with a maximum concentration of 0.5 mg mL^−1^ previously filtered through a Teflon filter with 0.22 µm pore size and employing PS as standard.

UV–vis spectroscopy was performed on an Agilent Cary 5000 UV–vis–NIR spectrophotometer.

Fluorescence spectra were acquired on a Horiba Jobin–Yvon Fluorolog‐3 spectrofluorometer equipped with a 450 W xenon arc lamp, double‐grating excitation, and single‐grating emission monochromator.

XPS survey spectra were obtained with a VG Escalab MKII (ENEA C.R. Casaccia, Italy) equipped with a twin anode (Mg, Al) X‐rays source, using Al X‐rays at 1486.6 eV, to assess the surface composition of the polymers.

Thin films were prepared via spin coating (1000 rpm, 60 s, 500 rpm s^−1^) from 10 mg mL^−1^ *o*‐dichlorobenzene solutions on glass slides previously washed with acetone, isopropanol, and treated with oxygen plasma (60 s, 50 W).

Cyclic voltammetry (CV) experiments were performed in a conventional three electrodes electrochemical cell using a Palmsens4 (Palmsens, The Netherland) employing a Pt wire as counter electrode and an Ag wire as reference in a 0.1 m tetrabutylammonium hexafluorophospahte (NBu_4_PF_6_) acetonitrile solution (scan rate 0.1 V s^−1^; E step size 0.05 V). The measurements were then referenced to the ferrocene/ferrocenium (Fc/Fc^+^) couple measured by adding Fc (5 mm) to the solution.

DFT calculations for optimized geometries, single‐point energies, and molecular orbitals of the polymers described in this study were calculated on thiophene terminated model tetramers employing Gaussian16 (ES64L‐G16RevC.01) and GaussView 6.1.1. In particular, the calculations were performed using B3LYP/6‐31g(d) basis set and employing GD3BJ empirical dispersion. Different orientation of the thiophene linkers were employed to evaluate the most stable conformation (see Figures S5–S7, Supporting Information). Orbital energy values are given considering the most stable conformation which may be different in the case of linkers comprising a different number of thiophene units.

### General Synthetic Procedure for HA‐Based Polymeric Precursors

All the HA‐based polymeric precursors were synthesized via a general Stille cross‐coupling procedure according to the previous work.^[^ ^26^ ^]^ The required 2,6‐dibromo HA‐based monomer (1 equiv.; see Supporting Information) was dissolved in toluene in a dry Schlenk tube. The solution was then degassed with N_2_ for 30 min. Following, the desired bis(trimethylstannyl)‐linker (1.05 equiv.) and XPhos Pd G2 (0.10 equiv.) were added and the mixture was heated up at 105 °C under stirring for 8 h, after which 1‐bromo‐4‐*tert*‐butylbenzene (excess) was added. The reaction was stirred at 105 °C for another hour, then the mixture was poured in methanol and left to flocculate for 30 min. The filtered product was then extracted with boiling acetone followed by boiling chloroform. The chloroform faction was reprecipitated in methanol to afford the final product. The characterization of the different polymers is reported in the Supporting Information (^1^H NMR, ^13^C NMR, FTIR, elemental analysis).

### General Procedure for the Preparation of AC‐Based Conjugated Polymers from HA Precursors

A procedure similar to that employed in the previous work was employed.^[^ ^26^ ^]^ In a 20 mL screw cap vial, 50 mg of HA‐precursor were suspended in 10 mL of a methanolic solution containing 50 mg of SnCl_2_ and 0.1 mL of HCl 37% at 50 °C for 10 min. Immediately after the addition, the polymer turned red, and a dim red fluorescence could be detected. The solid was then filtered and washed abundantly with methanol, water, acetone and let dry (99% yield). The characterization of the insoluble final polymer was carried out by elemental analysis and FTIR. A similar approach was employed for the transformation of the thin films.

### General Procedure for the Preparation of AQ‐Based Conjugated Polymers from HA Precursors

A procedure similar to that described in a previous work was employed.^[^ ^49^ ^]^ In a 20 mL screw cap vial, 50 mg of HA‐P were suspended in 5 mL of acetone containing 50 mg of TBAF trihydrate. The reaction was left for 10 min at 70 °C. Brown precipitate formed, which was filtered and washed abundantly with water and then acetone. The product was collected after drying (99% yield). The characterization of the insoluble final polymer was carried out by elemental analysis and FTIR. A similar approach was employed for the transformation of the thin films. In the latter case, washing was performed first with methanol to avoid delamination of the film.

### Fabrication and Characterization of OFET Devices

Organic thin‐film transistor‐substrate (OFET‐substrates) with four groups of four identical transistors per chip with a channel length of 2.5, 5, 10, 20 µm, respectively, have been purchased from Fraunhofer Institute for Photonic Microsystems IPMS (Dresden, Germany). Each transistor is a Generation IV—150 mm wafer with a gate oxide of 230 ± 10 nm SiO_2_. The protective resist was removed by boiling in acetone for 10 °C, and rinsing with acetone and isopropanol. The substrates were then treated with oxygen plasma (50 W, 60 s).

A 10 mg mL^−1^ solution of the precursor in *o*‐dichlorobenzene was spun‐coated on the substrate (1000 rpm, 60 s, 500 rpm s^−1^) and dried over a hot plate at 120 °C for 15 min. The conjugated anthracene polymer was prepared by immersion of the coated substrate in 10 mL of a methanol solution containing 100 mg of SnCl_2_ and 0.1 mL of HCl 12 m at 70 °C for 5 min. The sample was then rinsed with abundant methanol, water, and acetone, and it was dried over a hotplate for 30 min at 120 °C before measurement. The fabricated organic field effect transistors (with a channel length of 5 µm) were electrically characterized by using a two channel Source/Measure Unit (SMU) (model Keysight B2912A), and data were recorded through a custom software made in VB.NET (by Microsoft). Transfer curves were obtained applying 0, −10, −20, −30, and −40 V of VDS and sweeping VGS from 0 to −40 V with interval of 50 mV.

### Fabrication and Characterization of Organic Cathodes for K Ion Batteries

The organic material oAQ‐T solubility was tested by stirring it in different vials with dimethyl carbonate (DMC), ethylene carbonate:diethyl carbonate (EC:DEC) 1:1, triethylene glycol dimethyl ether:ethylene carbonate (TEGDME:EC) 1:1, and DME, respectively. The cathodes were fabricated by mixing each selected organic material (oAQ‐T and pAQ‐T) with C65 conductive carbon and polyvinylidene fluoride (PVDF) binder in a 50:40:10 ratio, using *N*‐methyl‐2‐pyrrolidone (NMP) as a solvent. The mixture was processed by ball milling for 20 min at 30 Hz. The resulting slurry was then coated onto an aluminum current collector using a doctor blade, with a wet thickness of 100 µm, and dried in an oven at 50 °C for 2 h to remove the solvent. Once dried, the electrodes were cut into 15 mm disks and further dried under vacuum in a Büchi oven at 120 °C for 4 h.

Cell assembly was carried out inside an argon‐filled glove box (MBRAUN MB10 compact, O₂ < 0.5 ppm, H₂O < 0.5 ppm) in a half‐cell configuration, using both coin cell (LIR2032) and ELCELL (ECC‐Std) architectures. Metallic potassium (Merck) disk (16 mm in diameter) served as both counter and reference electrode, while Whatman glass fiber disk (18 mm in diameter, 0.65 mm thick) was used as separator. The electrochemical performance was evaluated using 1 m potassium bis(fluorosulfonyl)imide in dimethoxyethane as the electrolyte. Galvanostatic cycling and cyclic voltammetry were performed within a voltage range of 1.2–3.5 V, with a current density of 0.05 A g^−1^ and a scan rate of 0.1 mV s^−1^, respectively.

## Conflict of Interest

The authors declare no conflict of interest.

## Supporting information



Supporting Information

## Data Availability

The data that support the findings of this study are available from the corresponding author upon reasonable request.
