# Supplementary material for: A Multipotent Precursor Approach for the Preparation of High‐Molecular Weight Conjugated Polymers with Redox Active Units
Source: Small Methods. 2025 Apr 30;9(9):2500488. doi: 10.1002/smtd.202500488 (PMC12464798; doi:10.1002/smtd.202500488)
Supplement: Supplementary file 1 — Supporting Information [file SMTD-9-2500488-s001.pdf]

small
methods

Supporting Information

for *Small Methods*, DOI 10.1002/smtd.202500488

A Multipotent Precursor Approach for the Preparation of High-Molecular Weight Conjugated Polymers with Redox Active Units

Benedetta Bertoncini, Andrea Taddeucci, Sabrina Trano, Sofia Raviolo, Ilaria Valdrighi, Federico Maria Vivaldi, Virgilio Mattoli, Federico Bella and Marco Carlotti**

Supporting Information

A multipotent precursor approach for the preparation of high-molecular weight conjugated polymers with redox active units

Benedetta Bertoncini, Andrea Taddeucci, Sabrina Trano, Sofia Raviolo, Ilaria Valdrighi, Federico Maria Vivaldi, Virgilio Mattoli, Federico Bella, Marco Carlotti**

Synthesis of the linkers

Synthesis of 2,2'-bithiazole

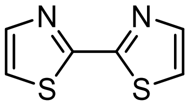

This reaction was performed following a previously reported procedure.[S1] In a 50 mL dry Schlenk, 7.59 g (46.27 mmol) of 2-bromothiazole, 0.52 g (2.31 mmol) od Pd(II) acetate, 5.98 g (46.27 mmol) of N,N-diisopropylamine, and 7.46 g (23.13 mmol) of tetrabutylammonium bromide were dissolved in 18 mL of anhydrous toluene. The reaction mixture was stirred at 105 °C for 41 h, then treated with water and extracted with dichloromethane. The organic phase was washed with water, dried over anhydrous $MgSO_4$, and the solvent was removed under vacuum to afford the crude. The crude was purified firstly with a plug column on silica gel eluting with petroleum ether/ethyl acetate (1:1) followed by recrystallization with heptane to afford 1.53 g (9.10 mmol, 39% yield) of 2,2'-bithiazole. ^{1}H-NMR (400 MHz, $CDCl_3$), δ (ppm): 8.09 (d, J = 2.1 Hz, 1H), 7.80 (d, J = 8.5 Hz, 1H), 7.53 (dd, J = 8.5, 2.1 Hz, 1H), 1.05 (s, 21H), -0.28 (s, 9H). These results are comparable with previously reported characterizations.[S1]

Synthesis of 5,5'-bis(trimethylstannyl)-2,2'-bithiazole

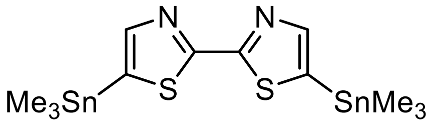

In a 250 mL dry flask, 0.76 g (4.52 mmol) of 2,2'-bithiazole were dissolved in 55 mL of anhydrous THF. The mixture was put at -78 °C with a N_2/ethanol bath, then 12.48 mL of lithium diisopropylamide 1.0 M in THF/hexane were added dropwise. The reaction mixture was left going back to room temperature. The reaction mixture was put again at -78 °C and 2.49 g (12.48

mmol) of trimethyl tin chloride were added. The mixture was left stirring while going back to room temperature and then overnight, before to be diluted with chloroform. The obtained organic solution was washed with water, dried over anhydrous $MgSO_4$ and the solvent was removed under vacuum to obtain the crude. The crude was purified via recrystallization in hexane to afford 1.58 g (3.20 mmol, 71% yield) of 5,5'-bis(trimethylstannyl)-2,2'-bithiazole. ^{1}H-NMR (400 MHz, $CDCl_3$) δ (ppm): 7.82 (s, 2H), 0.45 (s, 18H). ^{13}C-NMR (400 MHz, $CDCl_3$) δ (ppm): 166.26, 150.17, 132.15, -7.94. These results are comparable with previously reported characterizations.[S2]

Synthesis of the HA-monomers

General procedure for the synthesis of diBrHA-based monomeric precursors

All the HA-based monomeric precursors were synthesized from 2,6-dibromoanthraquinone via a general procedure derived from our previous works.[S3,S4]

2.55 eq of triisopropylsilyacetylene (TIPS-acetylene; R_1 = TIPS)/phenylacetylene (Ph-acetylene; R_1 = Ph)/1-octyne (R_1 = n-hexyl) were dissolved in of dry tetrahydrofuran (THF) and the solution was placed in an ice bath. Methyllithium 1.6 M in diethyl ether (2.5 eq) was added dropwise and the reaction was left for 20 min. 2,6-dibromoanthraquinone (1 eq) was added and the bath was removed. After 1 h, a large excess of trimethylsilyl chloride (TMSCl; R_2 = TMS)/iodomethane (CH_3I; R_2 = Me) was added. The solution was then extracted with a saturated aqueous ammonium chloride solution and dried over magnesium sulfate. The solvent was removed under vacuum, and the residue recrystallized twice from acetone to obtain the product.

diBrHA(TIPS)(OTMS): the product was obtained as a white powder (45% yield). ^{1}H-NMR (400 MHz, $CDCl_3$), δ (ppm): 8.09 (d, J = 2.1 Hz, 2H), 7.80 (d, J = 8.5 Hz, 2H), 7.53 (dd, J = 8.5, 2.1 Hz, 2H), 1.05 (s, 42H), -0.28 (s, 18H). ^{13}C-NMR (101 MHz, $CDCl_3$) δ (ppm): 138.67, 135.45, 132.29, 131.90, 131.05, 122.56, 111.16, 87.24, 67.97, 18.63, 11.32, 1.42. This was in line with previously reported characterizations.[S3]

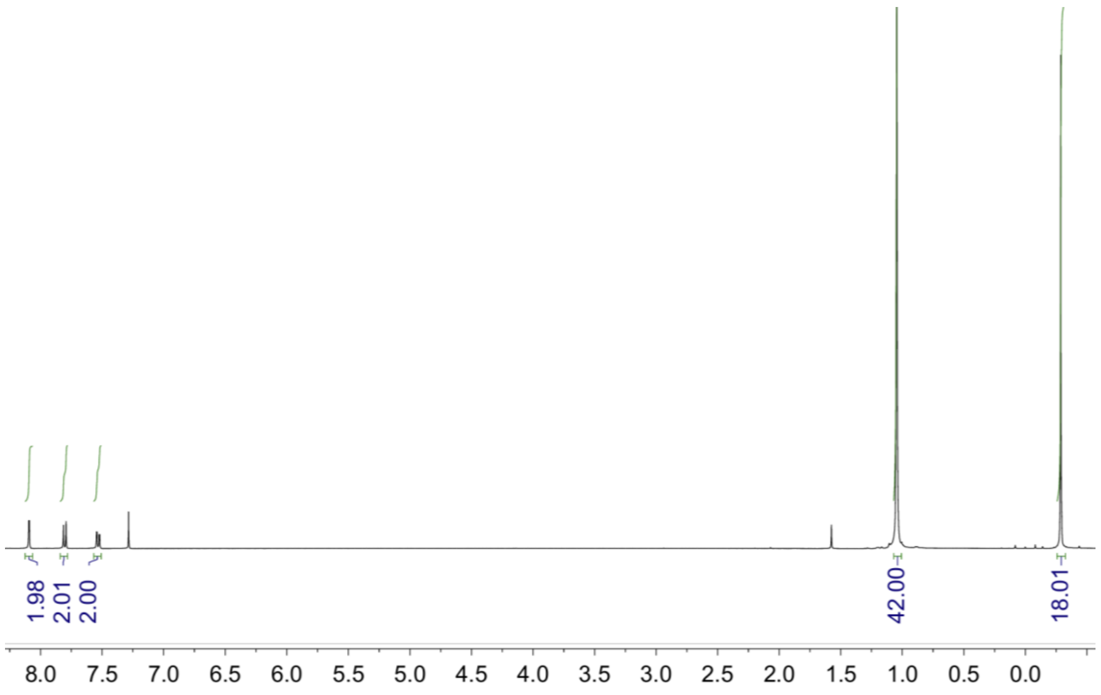

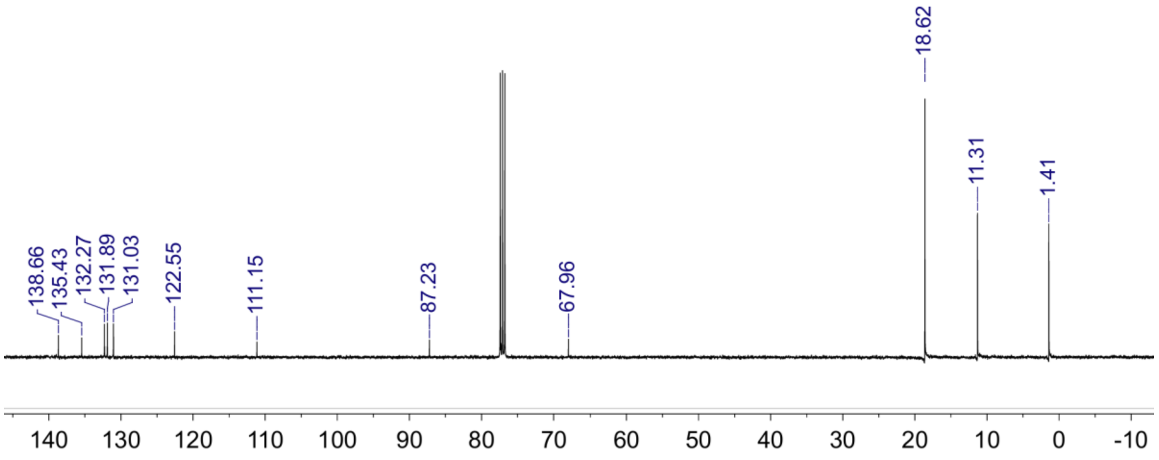

diBrHA(TIPS)(OMe): the product was obtained as a white powder (53% yield). ^{1}H-NMR (400 MHz, $CDCl_3$), δ (ppm): 8.14 (d, J = 2.1 Hz, 2H), 7.82 (d, J = 8.5 Hz, 2H), 7.61 (dd, J = 8.5, 2.1 Hz, 2H), 2.80 (s, 6H), 1.03 (d, J = 2.8 Hz, 42H). ^{13}C-NMR (101 MHz, $CDCl_3$) δ (ppm): 137.82, 134.40, 132.40, 131.26, 130.00, 123.33, 108.01, 89.39, 72.03, 51.33, 18.61, 11.18. This was in line with previously reported characterizations.[S3]

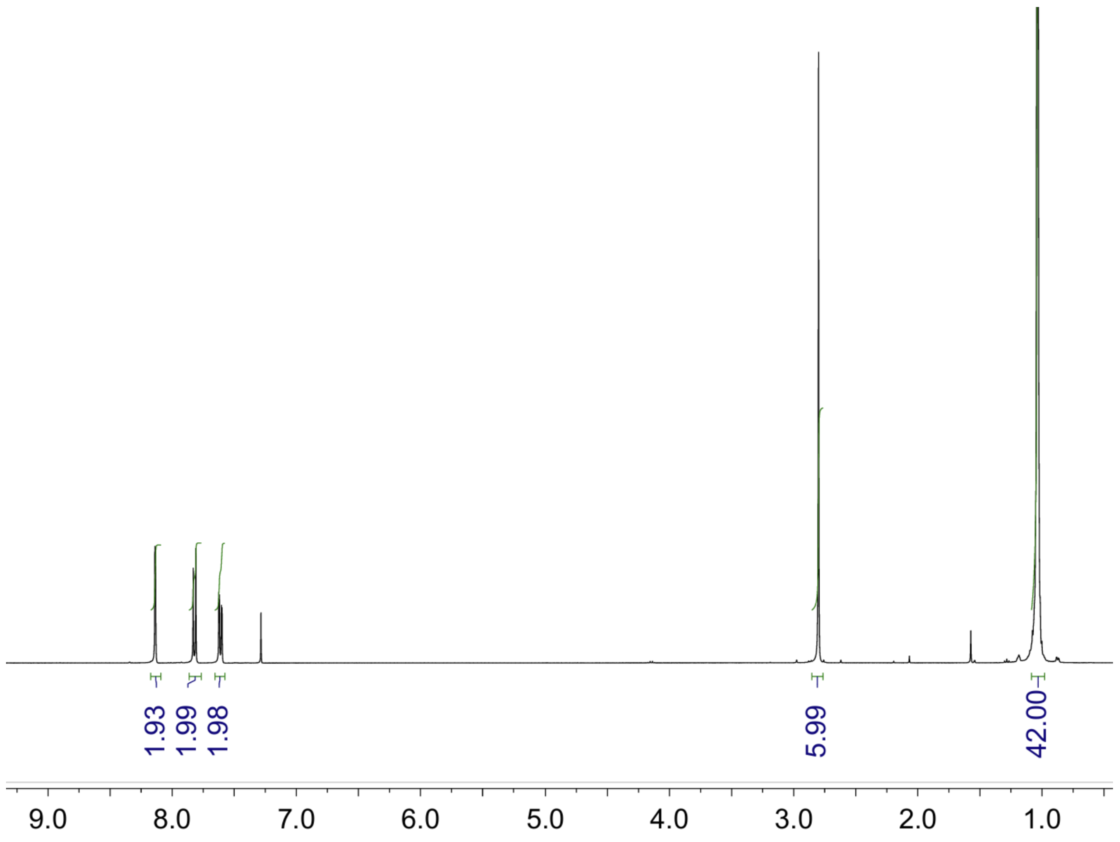

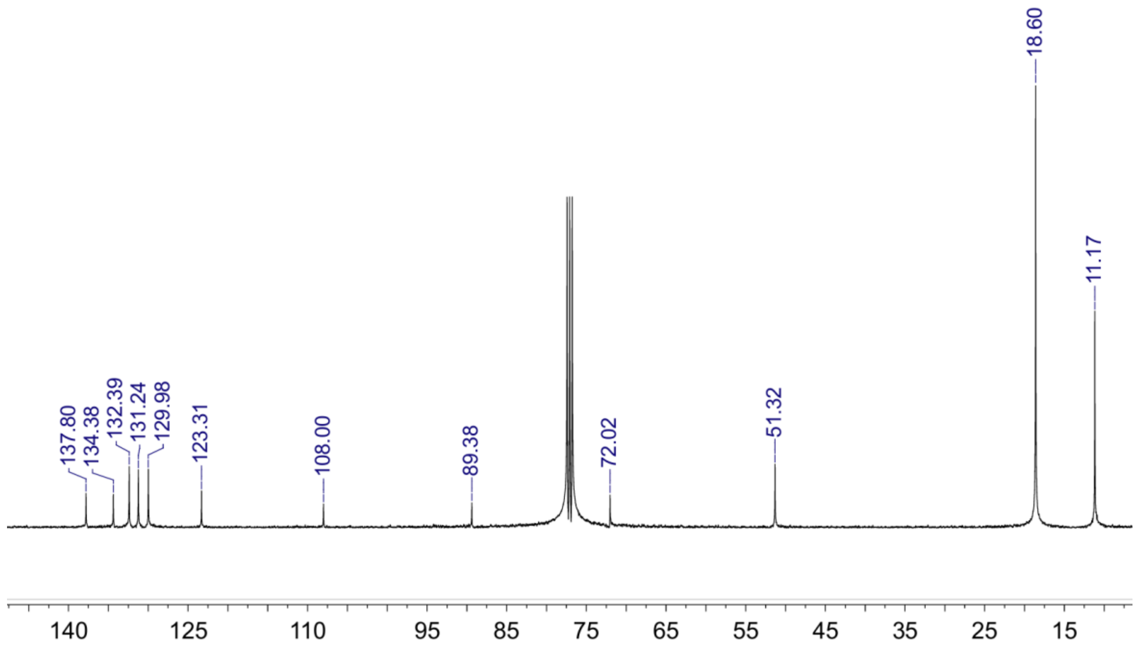

diBrHA(Ph)(OTMS): the product was obtained as a white powder (44% yield). ^{1}H-NMR (400 MHz, $CDCl_3$), δ (ppm): 8.13 (d, J = 2.1 Hz, 2H), 7.88 (d, J = 8.4 Hz, 2H), 7.56 (dd, J = 8.5, 2.1 Hz, 2H), 7.43 – 7.36 (m, 4H), 7.30 (m, 6H), -0.06 (s, 18H). ^{13}C-NMR (101 MHz, $CDCl_3$) δ (ppm): 139.76, 136.73, 131.87, 131.63, 131.04, 129.97, 128.70, 128.30, 122.70, 122.34, 92.11, 87.07, 76.70, 68.93, 1.64. This was in line with previously reported characterizations.[S3]

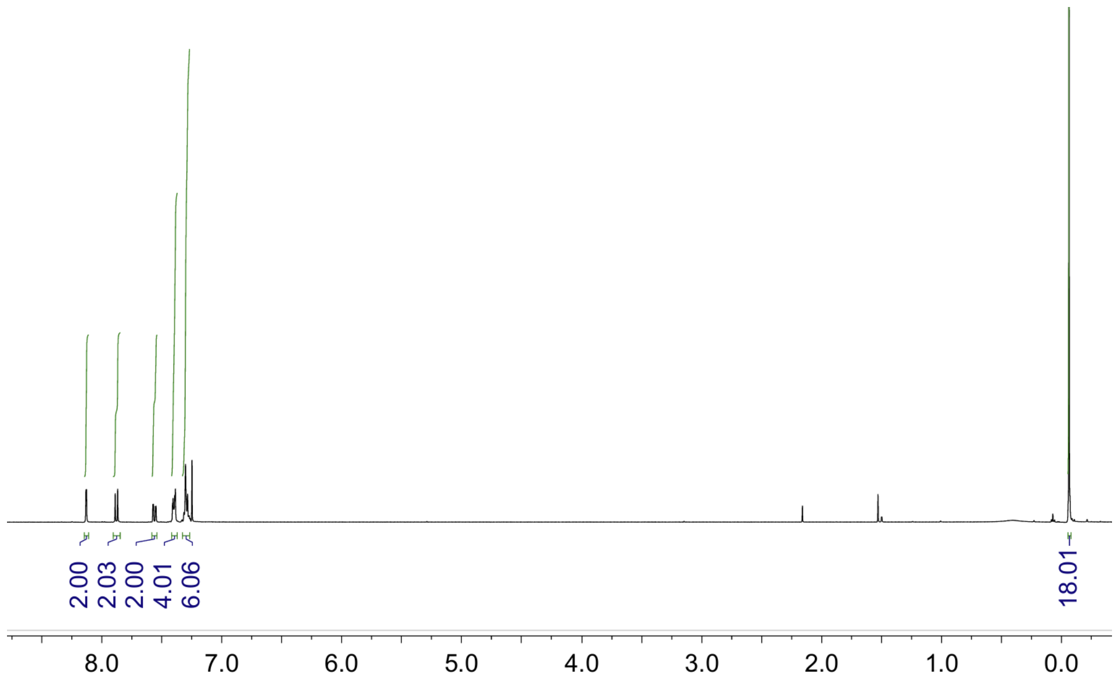

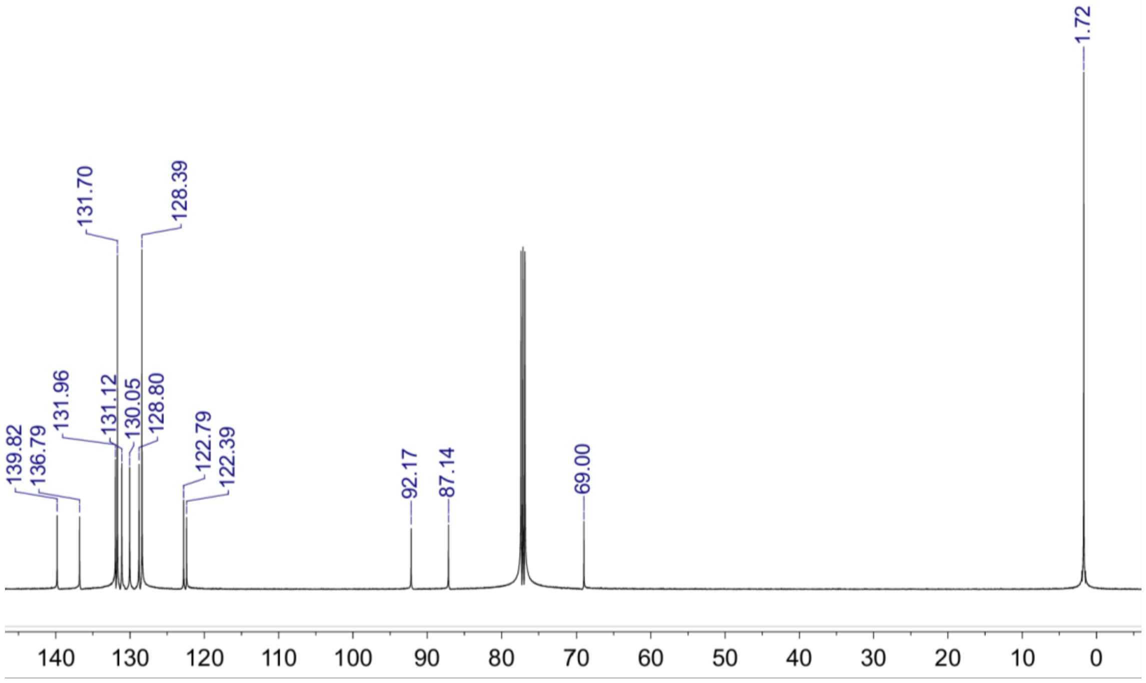

diBrHA(Hex)(OTMS): the product was obtained as a white powder (22% yield). ^{1}H-NMR (400 MHz, CDCl$_3$), δ (ppm): 8.05 (d, J = 2.1 Hz, 2H), 7.79 (d, J = 8.5 Hz, 2H), 7.52 (dd, J = 8.4, 2.1 Hz, 2H), 2.24 (t, J = 7.1 Hz, 4H), 1.56 – 1.42 (m, 4H), 1.39 – 1.18 (m, 4H), 0.88 (t, J = 6.8 Hz, 6H), - 0.11 (s, 18H). ^{13}C-NMR (101 MHz, CDCl$_3$) δ (ppm): 139.87, 136.80, 131.58, 131.19, 130.15, 122.38, 88.16, 83.87, 68.45, 31.35, 28.66, 22.61, 19.08, 14.12, 1.63. This was in line with previously reported characterizations.[S3]

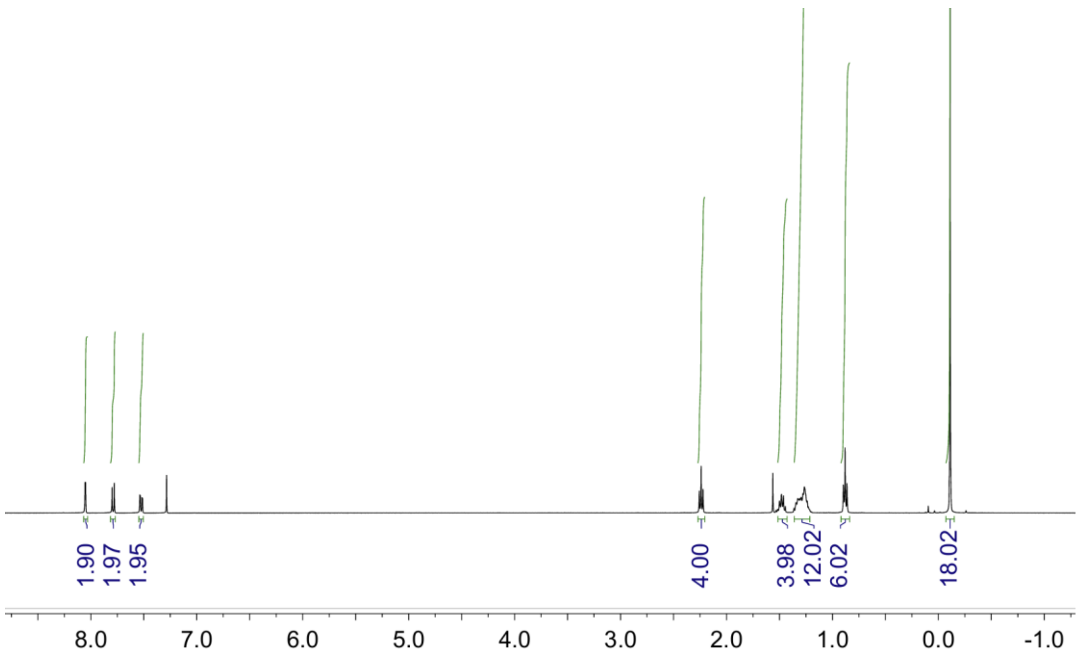

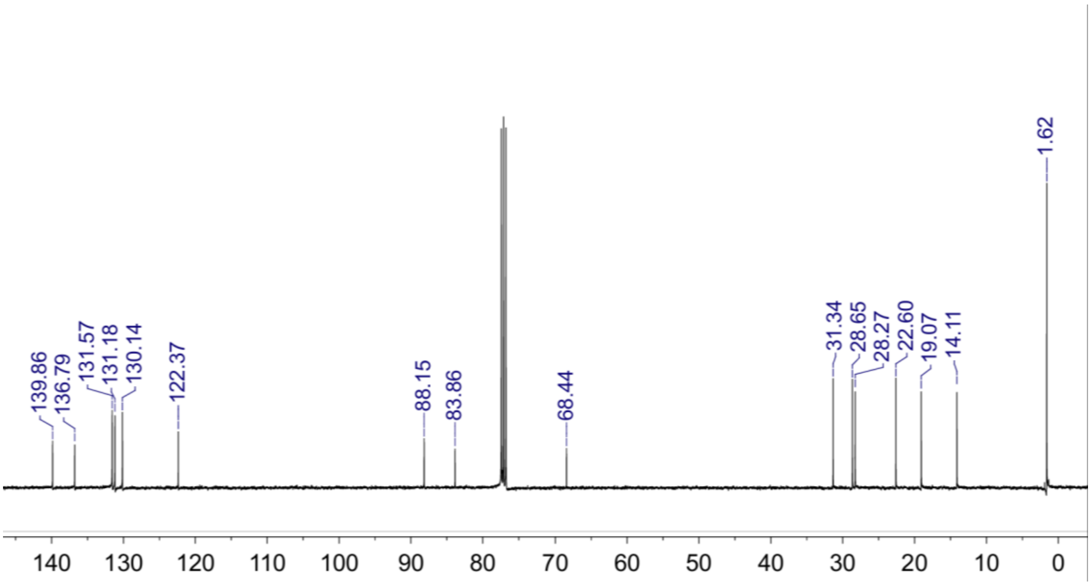

diBrHA(Ph)(OMe): the product was obtained as a white powder (54% yield). ^{1}H-NMR (400 MHz, $CDCl_3$), δ (ppm): (d, J = 2.1 Hz, 2H), 7.89 (d, J=8.4 Hz, 2H), 7.63 (dd, J = 8.4 Hz 2.1 Hz, 2H), 7.44-7.38 (m, 4H), 7.33–7.26 (m, 6H), 2.92 (s, 6H). ^{13}C-NMR (101 MHz, $CDCl_3$) δ (ppm): 137.76, 134.59, 132.73, 131.87, 131.15, 130.21, 128.98, 128.39, 123.69, 122.05, 89.94, 87.71, 72.39, 51.72.

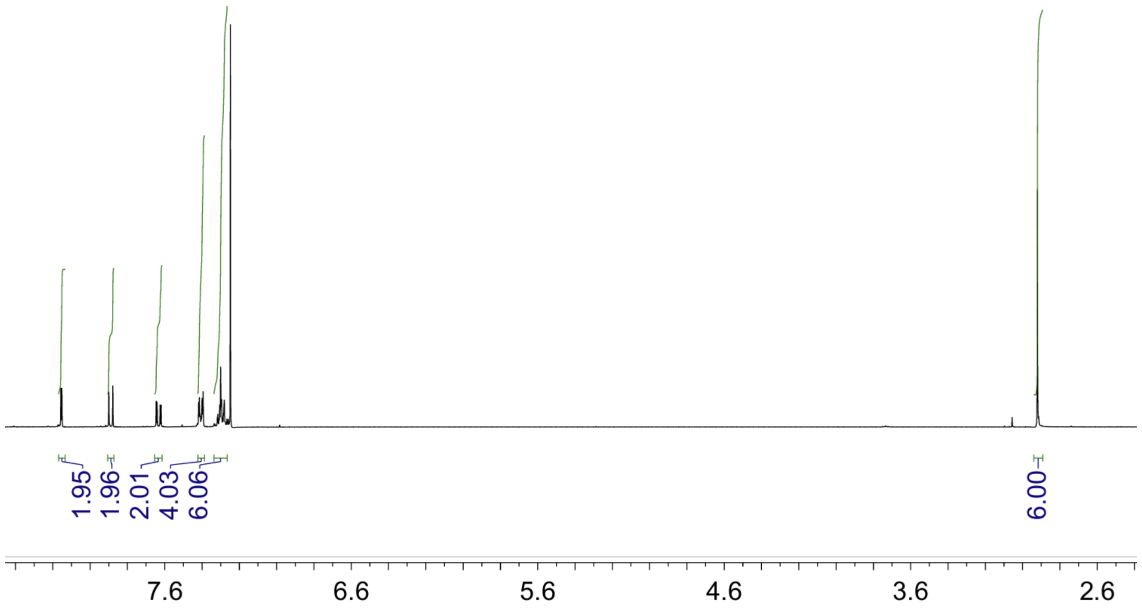

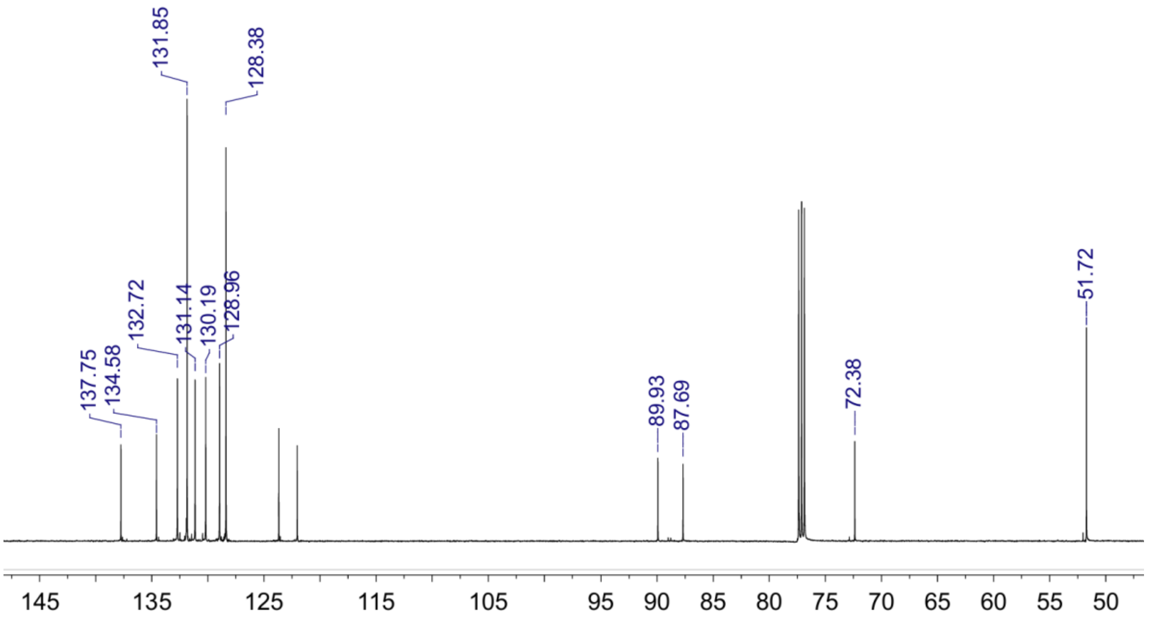

diBrHA(TMS)(OTMS): the product was obtained as a white powder (64% yield). ^{1}H-NMR (400 MHz, $CDCl_3$), δ (ppm): 8.06 (d, J = 2.0 Hz, 2H), 7.79 (d, J = 8.5 Hz, 2H), 7.55 (dd, J = 8.5, 2.0 Hz, 2H), 0.16 (s, 18H), -0.10 (s, 18H). ^{13}C-NMR (101 MHz, $CDCl_3$) δ (ppm): 139.32, 136.26, 131.87, 131.34, 130.25, 122.64, 108.18, 91.79, 68.50, 1.69, -0.28.

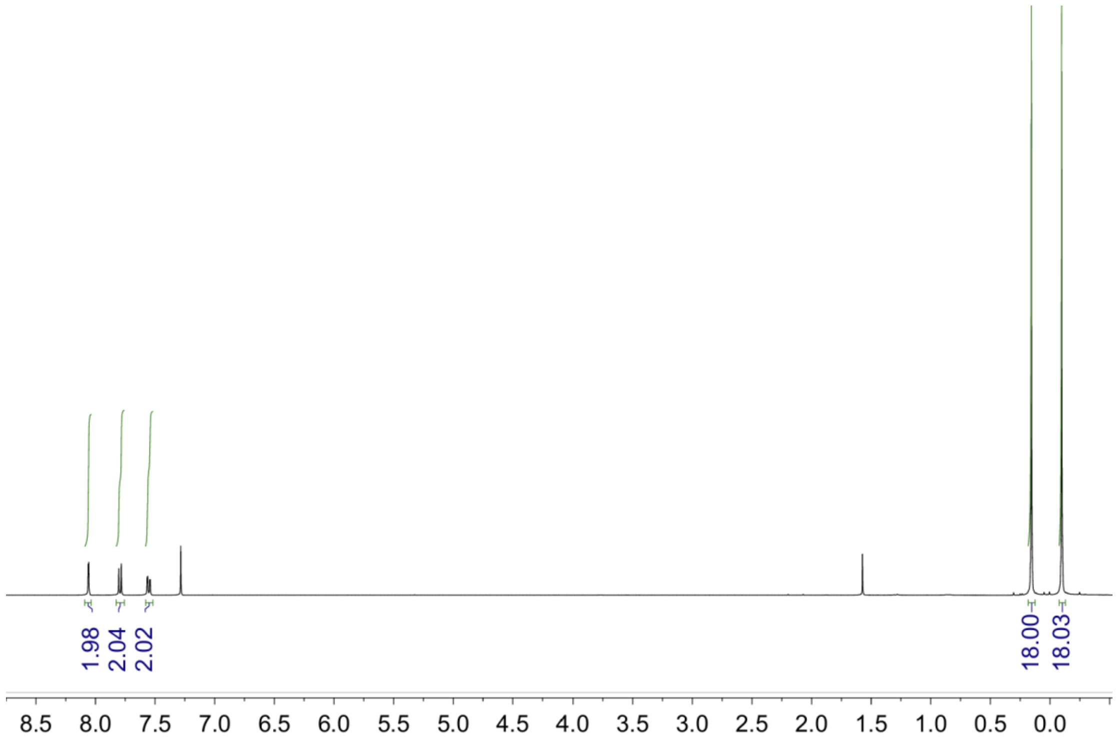

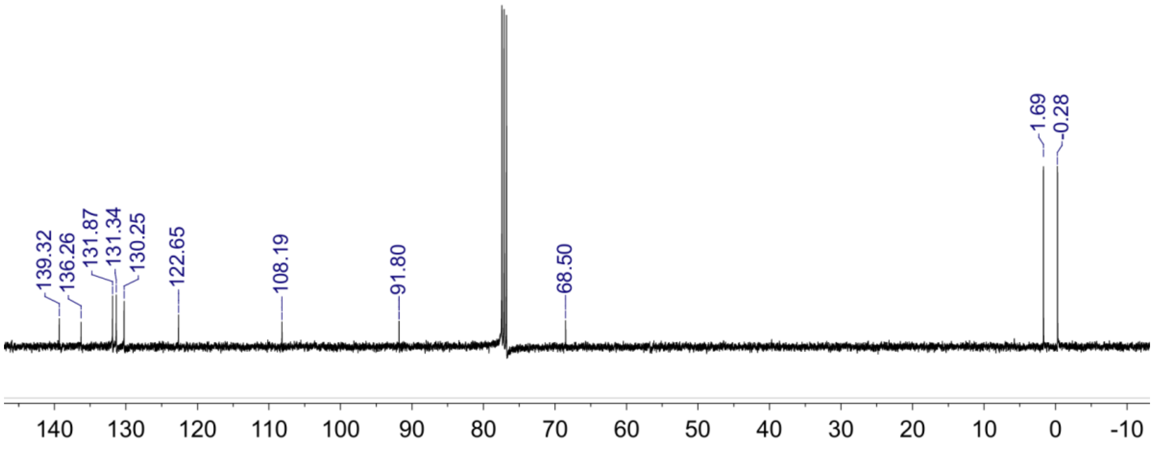

Synthesis of ((2,5-dibromo-1,4-bis(phenylethynyl)cyclohexa-2,5-diene-1,4-diyl)bis(oxy)) bis(trimethyl-silane) – diBrBQ(Ph)(OTMS)

2.55 eq of phenylacetylene were dissolved in of dry tetrahydrofuran (THF) and the solution was placed in an ice bath. Methyllithium 1.6 M in diethyl ether (2.5 eq) was added dropwise and the reaction was left for 20 min. 2,5-dibromo-1,4-benzoquinone (1 eq) was added and the bath was removed. The solution was allowed to react for 4 h at room temperature, then a large excess of trimethylsilyl chloride (TMSCl; R_2 = TMS) was added. The solution was then extracted with a saturated aqueous ammonium chloride solution and dried over magnesium sulfate. The solvent was removed under vacuum, and the residue recrystallized twice from acetone to obtain the product as a white powder (14% yield). ^{1}H-NMR (400 MHz, $CDCl_3$), δ (ppm): 7.47-7.43 (m, 4H), 7.35-7.28 (m, 6H), 6.39 (s, 2H), 0.27 (s, 18H). Elem. Anal. $C_{28}H_{30}Br_2O_2Si_2$. Calc. (%) C 54.73, H 4.92, Br 26.01, O 5.21, Si 9.14. Exp. (%) C 54.04, H 4.90.

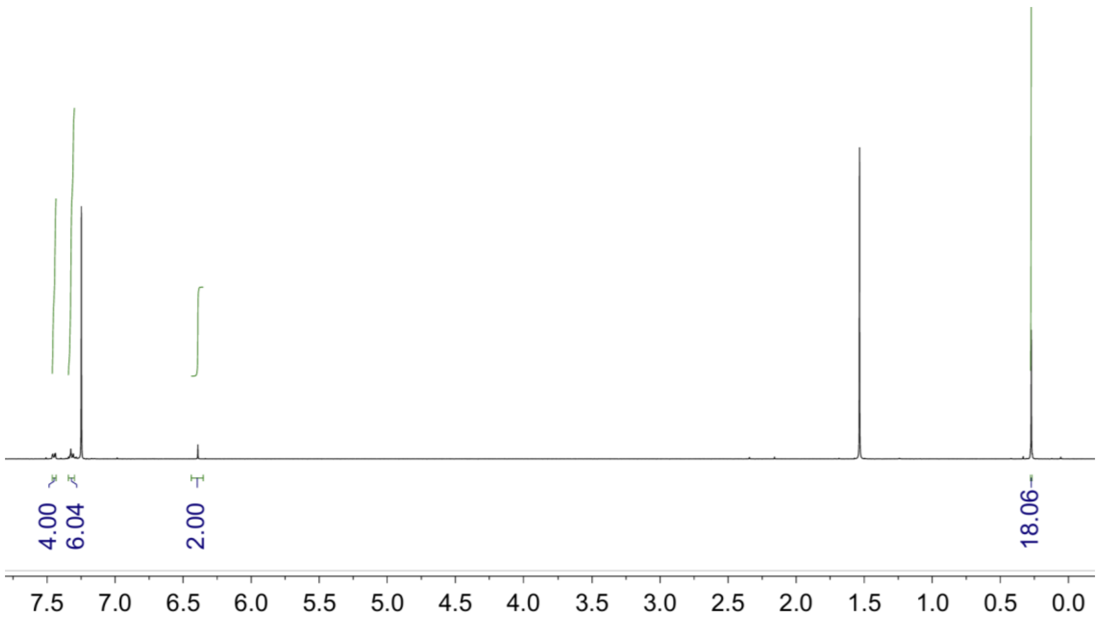

Synthesis of ((2,7-dibromo-9-(phenylethynyl)-9H-fluoren-9-yl)oxy)trimethylsilane – diBr(Ph)(OTMS)FL

1.55 eq of phenylacetylene were dissolved in of dry tetrahydrofuran (THF) and the solution was placed in an ice bath. Methyllithium 1.6 M in diethyl ether (1.5 eq) was added dropwise and the reaction was left for 20 min. 2,7-dibromo-9-fluorenone (1 eq) was added and the bath was removed. The solution was allowed to react for 3 h at 50 °C using an oil bath, then a large excess of trimethylsilyl chloride (TMSCl; R_2 = TMS) was added. The solution was then extracted with a saturated aqueous ammonium chloride solution and dried over magnesium sulfate. The solvent was removed under vacuum, and the residue recrystallized twice from acetone to obtain the product as a white powder (65% yield). ^{1}H-NMR (400 MHz, $CDCl_3$), δ (ppm): 7.77 (dd, J = 1.8 Hz, 0.4 Hz, 2H), 7.52 (d, J = 1.8 Hz, 1H), 7.50 (d, J = 1.8 Hz, 1H), 7.45 (s, 1H), 7.43 (s, 1H), 7.42 (d, J = 1.6 Hz, 1 H), 7.40 (d, J = 2.0 Hz, 1H), 7.31-7.27 (m, 3H), 0.11 (s, 9 H).

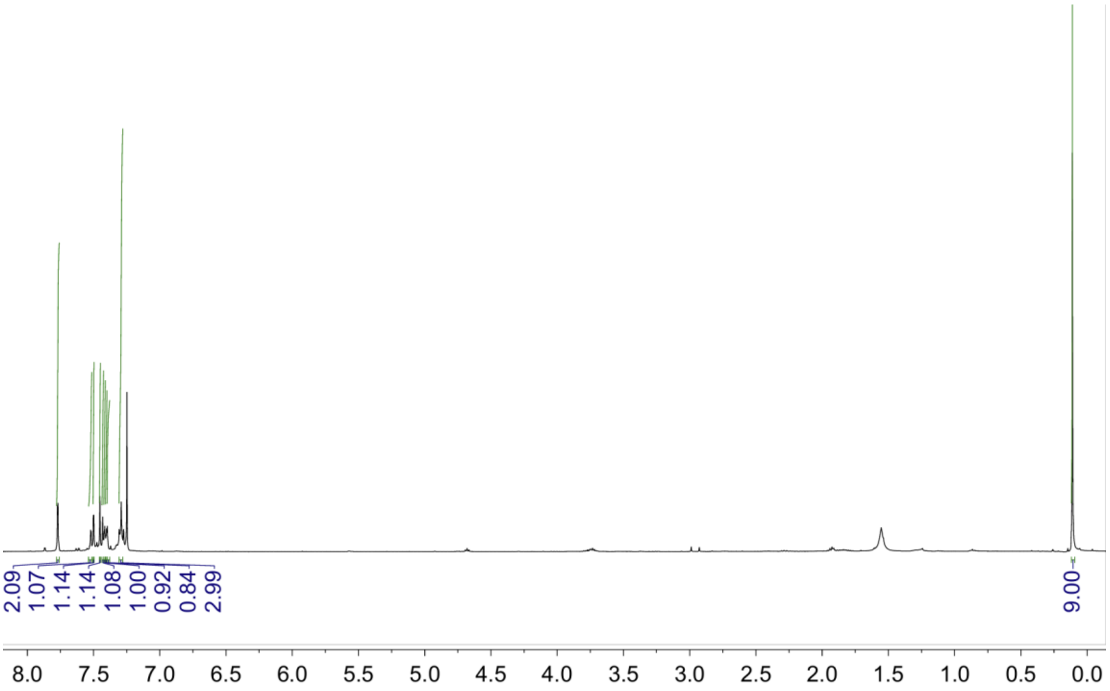

Synthesis of ((2,5-dibromo-1,4-bis((triisopropylsilyl)ethynyl)cyclohexa-2,5-diene-1,4-diyl)bis(oxy))bis-(trimethylsilane) – diBr(TIPS)(OTMS)BP

1.3 eq of triisopropylsilyacetylene were dissolved in dry tetrahydrofuran (THF) and the solution was placed in an ice bath. Methyllithium 1.6 M in diethyl ether (1.25 eq) was added dropwise and the reaction was left for 20 min. Benzophenone (1 eq) was added, and the bath was removed. After reacting for 2 h at room temperature, the solution was extracted with a saturated aqueous ammonium chloride solution and dried over magnesium sulphate. The solvent was removed under vacuum, and the residue recrystallized from hexane. The intermediate product was dissolved in anhydrous DCM with imidazole (6 eq) and a large excess of trimethylsilyl chloride (TMSCl; R_2 = TMS). The solution was allowed to react overnight. The solution was extracted with a saturated aqueous ammonium chloride solution and dried over magnesium sulphate. The solvent was removed under vacuum, and the residue recrystallized twice from acetone to obtain the product as a green viscous oil (87% yield). ^{1}H-NMR (400 MHz, $CDCl_3$), δ (ppm): 7.42-7.36 (m, 8H), 1.11 (s, 21H), 0.14 (s, 9H).

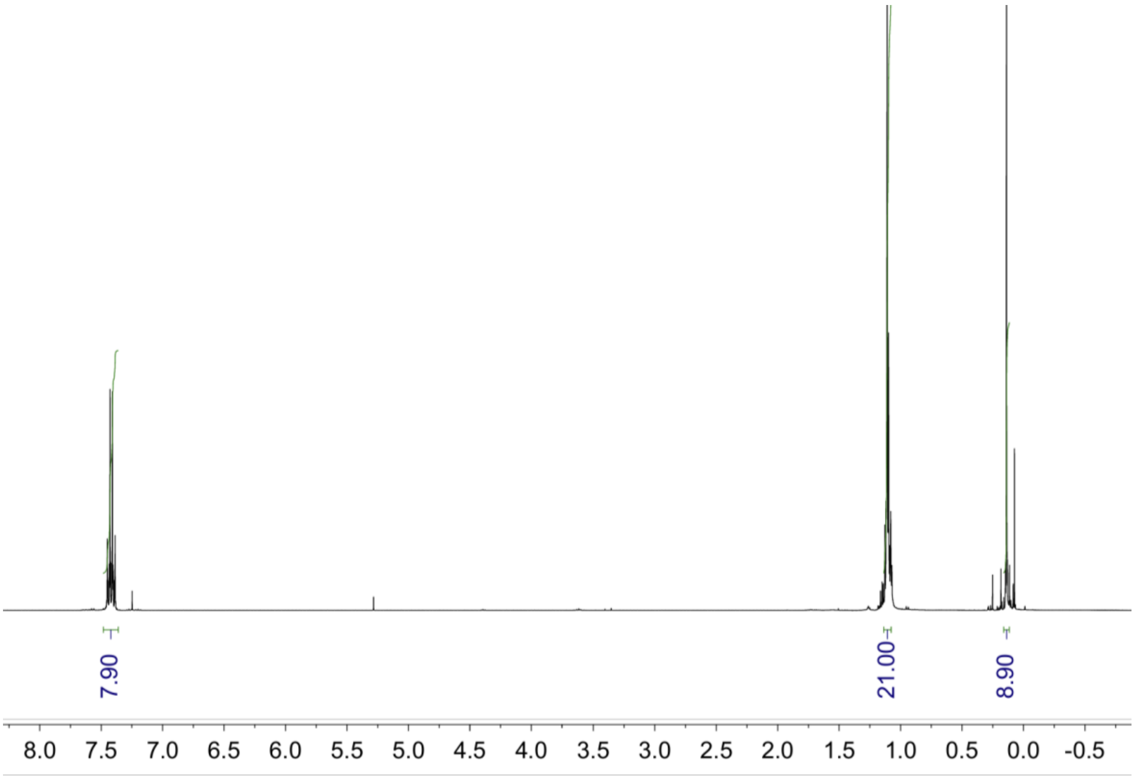

Synthesis of the precursor polymers

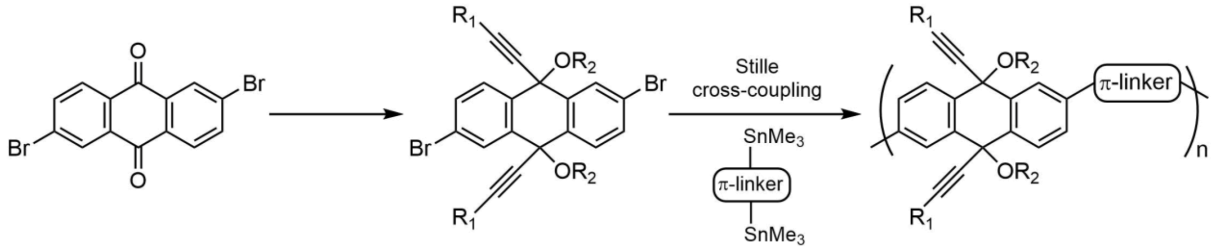

Scheme S1. General synthetic strategies to obtain HA-based polymeric precursors.

HA(Ph)(OTMS)-T: The polymer was obtained as a yellow powder (83% yield). ^{1}H-NMR (400 MHz, $CDCl_3$), δ (ppm): 8.39 (s, 2H), 8.13 (d, J = 8.1 Hz, 2H), 7.80 (d, J = 8.5 Hz, 2H), 7.48 (d, J = 7.0 Hz, 6H), 7.32 (d, J = 4.6 Hz, 6H), 0.11-0.00 (m, 18H). Elem. Anal. $C_{40}H_{38}O_2SSi_2$. Calc. C 75.19, H 5.99, O 5.01, S 5.02, Si 8.79. Exp. (%) C 75.28, H 5.783, S 5.079.

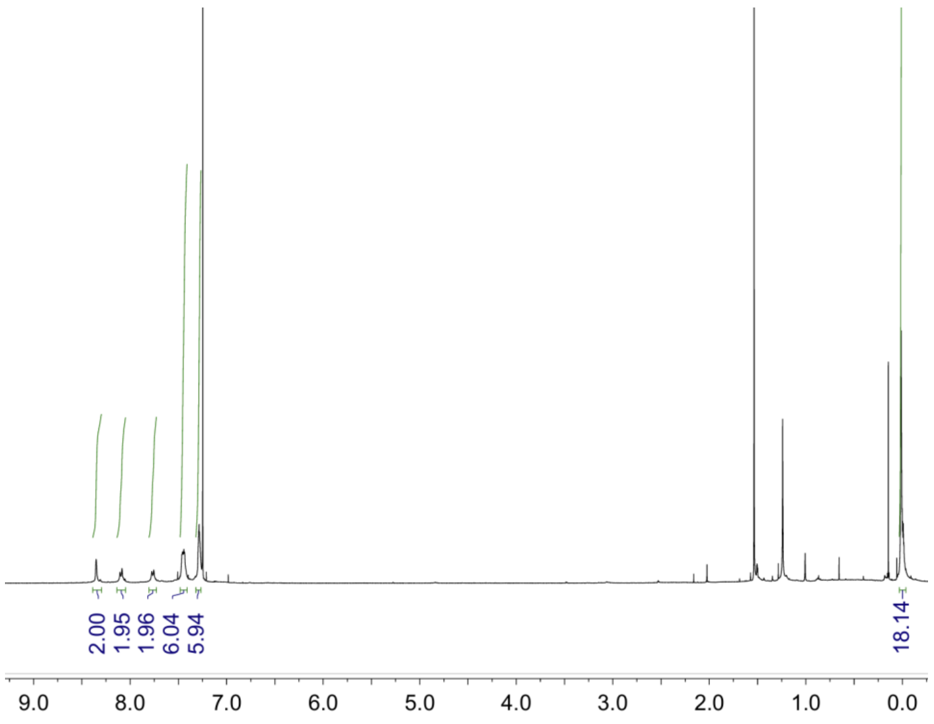

HA(Ph)(OMe)-T: The polymer was obtained as a green powder (85% yield). ^{1}H-NMR (400 MHz, $CDCl_3$), δ (ppm): 8.35 (s, 2H), 8.11 (d, J = 8.2 Hz, 2H), 7.82 (d, J = 8.4 Hz, 2H), 7.52-7.44 (m, 8H), 7.32-7.26 (m, 4H), 3.02 (s, 6H). Elem. Anal. $C_{36}H_{26}O_2S$. Calc. (%) C 82.73, H 5.01, O 6.12, S 6.13. Exp. (%) C 82.91, H 4.95, S 6.05.

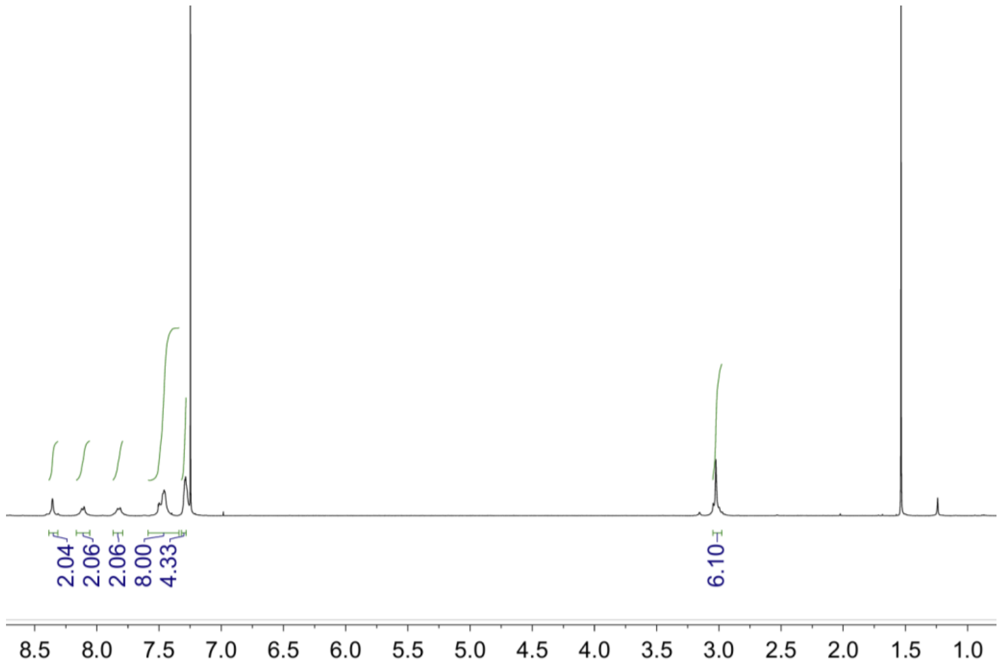

HA(TIPS)(OTMS)-T: The polymer was obtained as a dark green powder (72% yield). ^{1}H-NMR (400 MHz, $CDCl_3$), δ (ppm): 8.31 (s, 2H), 8.03 (d, J = 7.6 Hz, 2H), 7.75 (d, J = 7.3 Hz, 2H), 7.49 (d, 2H), 1.11 (s, 42 H), -0.22 (s, 18H). ^{13}C-NMR (101 MHz, $CDCl_3$) δ (ppm): 143.34, 143.25, 137.40, 137.38, 136.06, 135.97, 134.31, 134.27, 130.00, 126.14, 126.00, 125.87, 125.73, 125.48, 125.41, 124.25, 124.19, 112.10, 112.06, 86.36, 86.28, 86.25, 86.19, 68.46, 68.44, 68.42, 68.41, 31.30, 18.67, 11.38, 1.48. Elem. Anal. $C_{46}H_{70}O_2SSi_4$ Calc. (%) C 69.11, H 8.83, O 4.00, S 4.01, Si 14.05. Exp. (%) C 69.24, H 8.97, S 3.91.

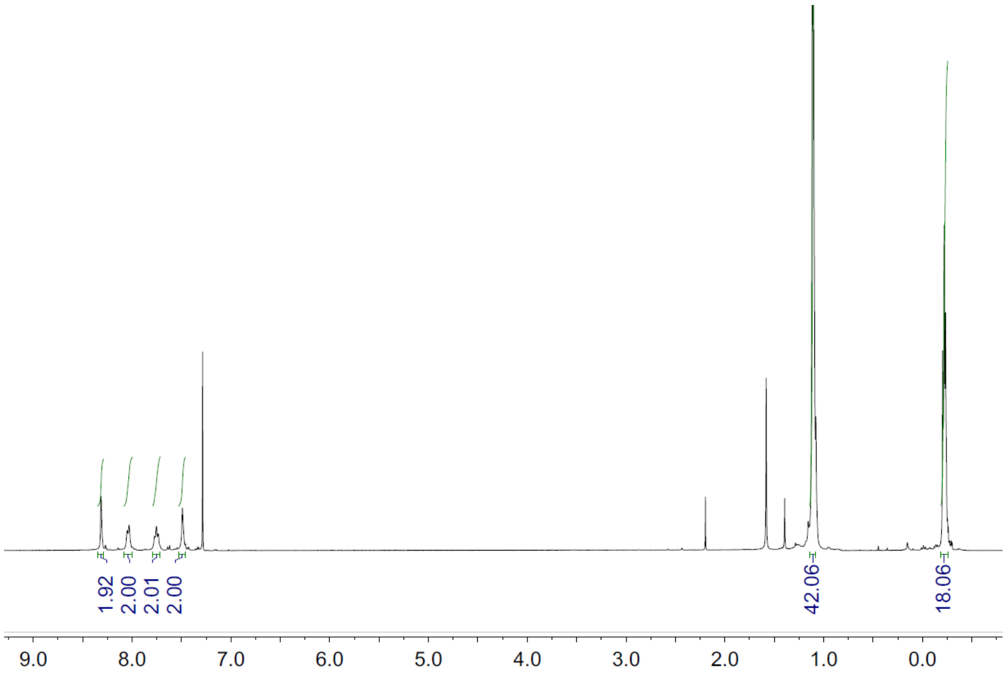

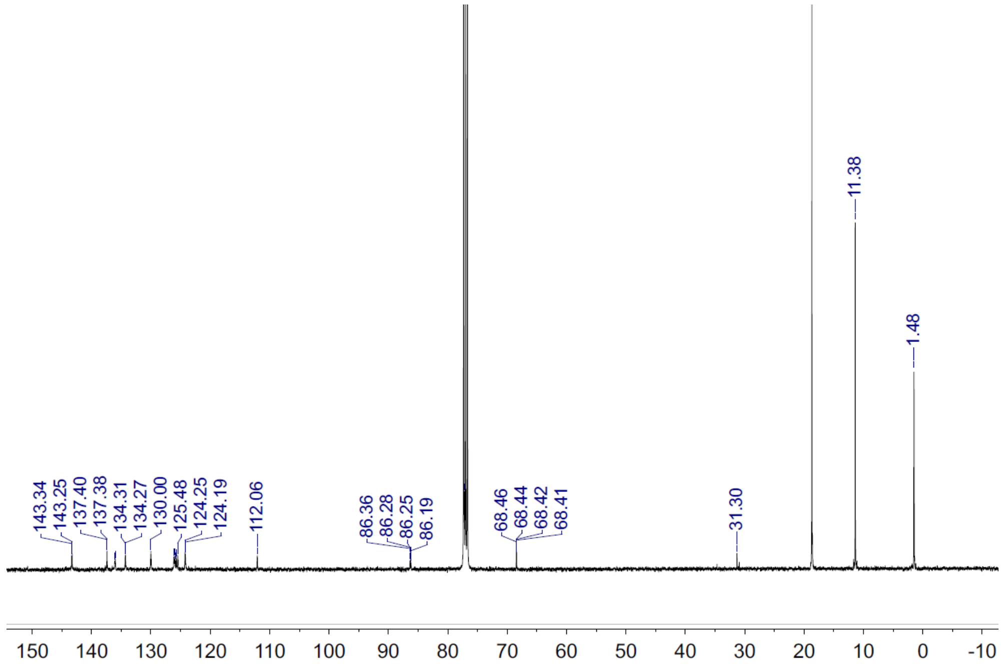

HA(TIPS)(OTMS)-Tz: The polymer was obtained as a dark green powder (74% yield). ^{1}H-NMR (400 MHz, $CDCl_3$), δ (ppm): ^{1}H NMR (400 MHz, $CDCl_3$), δ (ppm): 8.29 (s, 2H), 8.22 (s, 2H), 8.07 (d, J = 7.6 Hz, 2H), 7.74 (d, J = 7.9 Hz, 2H). 1.08 (s, 42H), -0.19 (s, 18H). Elem. Anal. $C_{46}H_{68}N_2O_2S_2Si_4$. Calc. (%) C 64.43, H 7.99, N 3.27, O 3.73, S 7.48, Si 13.10. Exp. (%) C 64.53, H 8.033, N 3.13, S 7.397.

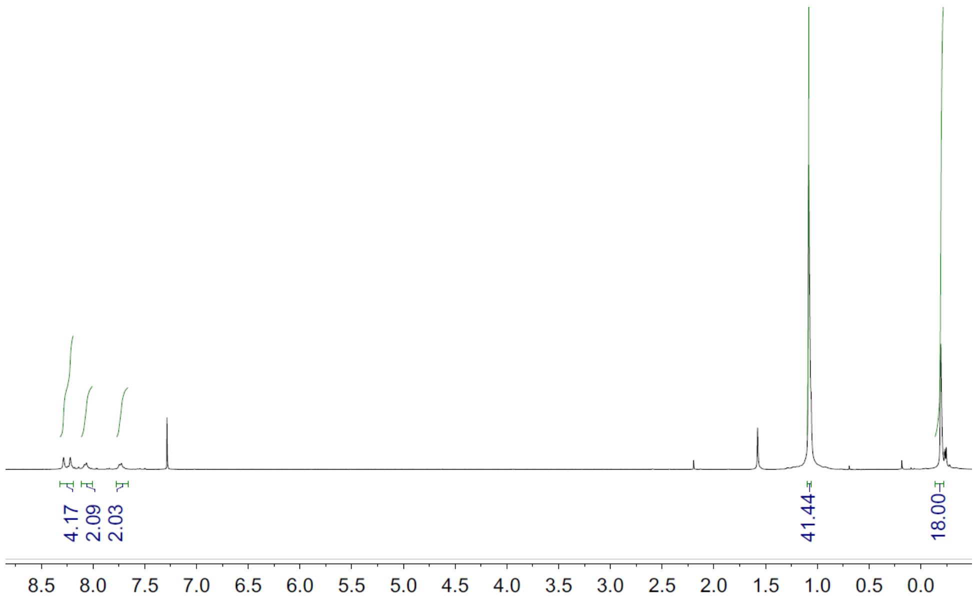

HA(Hex)(OTMS)-T: The polymer was obtained as a dark green powder (82% yield). ^{1}H-NMR (400 MHz, $CDCl_3$), δ (ppm): NMR (400 MHz, $CDCl_3$): δ 8.23 (s, 2H), 7-98-7.96 (d, J = 7.98 Hz, 2H), 7.71-7.69 (d, J = 7.69 Hz, 2H), 7.44 (s, 2H), 2.28-2.24 (m, 4H), 1.51-1.47 (m, 4H), 1.32 (s br, 4H), 1.20 (s br, 8H), 0.80 (s, 6H), -0.07 (s, 18 H). Elem. Anal. $C_{40}H_{54}O_2SSi_2$. Calc. (%) C 73.34, H 8.31, O 4.88, S 4.89, Si 8.57. Exp. (%) C 73.45, H 8.44, S 4.80.

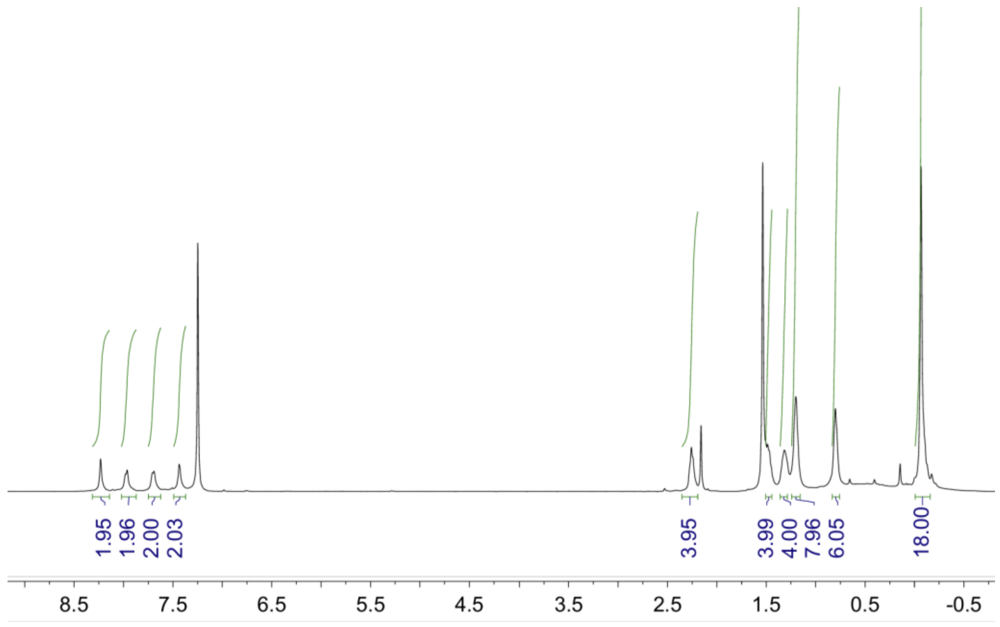

HA(Ph)(OMe)-BT: The polymer was obtained as a dark orange powder (87% yield). ^{1}H-NMR (400 MHz, $CDCl_3$), δ (ppm): 8.30 (s, 2H), 8.09 (d, J = 8.0 Hz, 2H), 7.78 (d, J = 8.5 Hz, 2H), 7.50-7.43 (m, 4H), 7.34-7.27 (m, 6H), 3.01 (s, 6H). ^{13}C-NMR (101MHz, $CDCl_3$), δ (ppm): 142.44, 137.31, 136.61, 134.99, 131.83, 129.38, 129.15, 128.69, 128.27, 126.46, 125.03, 124.89, 124.75, 122.37, 90.84, 87.17, 72.70, 51.57. Elem. Anal. $C_{40}H_{28}O_2S_2$. Calc. (%) C 79.44, H 4.67, O 5.29, S 10.60. Exp. (%) C 79.19, H 4.525, S 10.608.

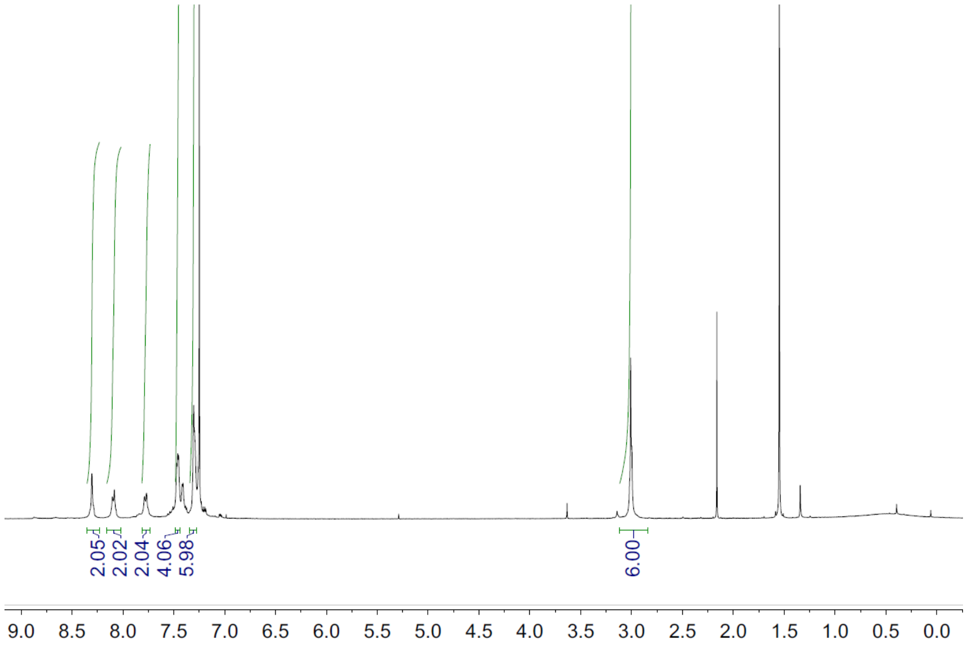

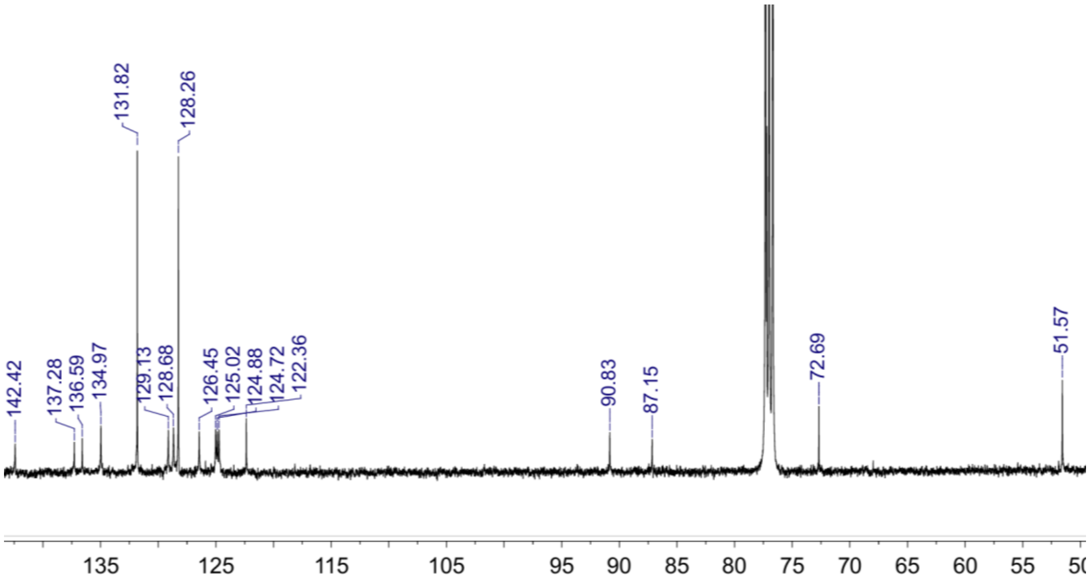

HA(Ph)(OTMS)-Tz: The polymer was obtained as a dark green powder (71% yield). ^{1}H-NMR (400 MHz, $CDCl_3$), δ (ppm): 8.35 (s, 2H), 8.22 (s, 2H), 8.15 (s, J = 7.6 Hz, 2H), 7.77 (d, J = 7.6 Hz, 2H), 7.46 (m, 4H), 7.34 (m, 6H), 0.09 (s, 18H). Elem. Anal. $C_{42}H_{36}N_2O_2S_2Si_2$. Calc. (%) C 69.96, H 5.03, N 3.89, O 4.44, S 8.89, Si 7.79. Exp. (%) C 69.65, H 4.89, N 3.76, S 9.020.

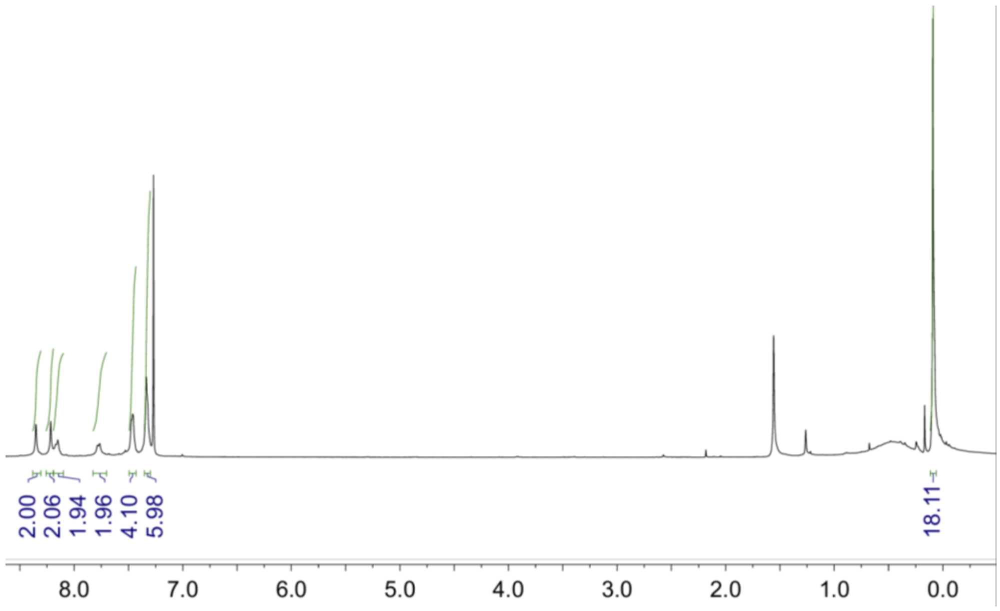

HA(TMS)(OTMS)-BT: The polymer was obtained as a yellow powder (81% yield). ^{1}H-NMR (400 MHz, $CDCl_3$), δ (ppm): δ 8.24 (s, 2H), 8.00 (d, J = 7.9 Hz, 2H), 7.73 (d, J = 8.2 Hz, 2H), 7.41 (s, 2H), 7.27 (2H, under solvent signal) 0.21 (s, 18H), -0.00 (s, 18H). Elem. Anal. $C_{36}H_{46}O_2S_2Si_4$. Calc. (%) C 62.92, H 6.75, O 4.66, S 9.33, Si 16.35. Exp. (%) C 62.82, H 7.983, S 9.179.

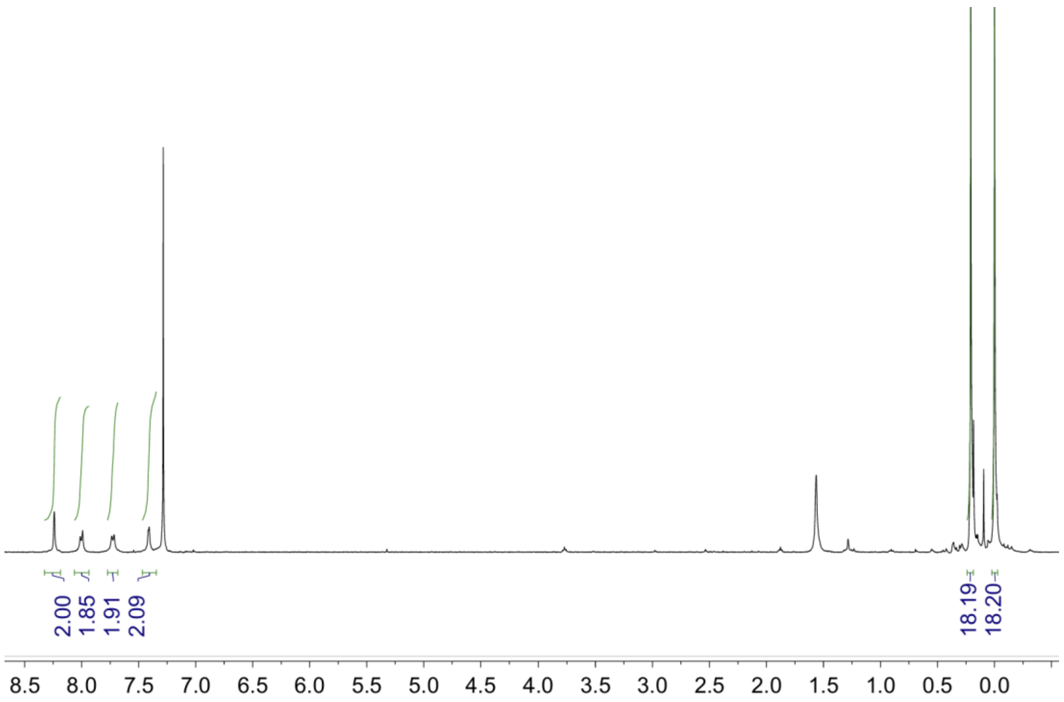

HA(Ph)(OTMS)-BT: The polymer was obtained as an orange powder (83% yield). ^{1}H-NMR (400 MHz, $CDCl_3$) δ (ppm): 8.34 (s, 2H), 8.12 (d, J = 7.7 Hz, 2H), 7.76 (d, J = 8.3 Hz, 2H), 7.55-7.45 (m, 4H), 7.42 (s, 2H), 7.38-7.31 (m, 6H), 0.04 (d, J = 2.1 Hz, 18H). ^{13}C-NMR (101 MHz, $CDCl_3$) δ (ppm): 142.80, 138.58, 137.23, 137.06, 134.23, 129.01, 128.49, 128.24, 125.71, 125.19, 124.84, 124.28, 122.75, 93.09, 86.72, 69.39, 1.79. Elem. Anal. $C_{44}H_{38}O_2S_2Si_2$. Calc. (%) C 73.49, H 5.33, O 4.45, S 8.92, Si 7.81. Exp. (%) C 73.18, H 4.681, S 8.603.

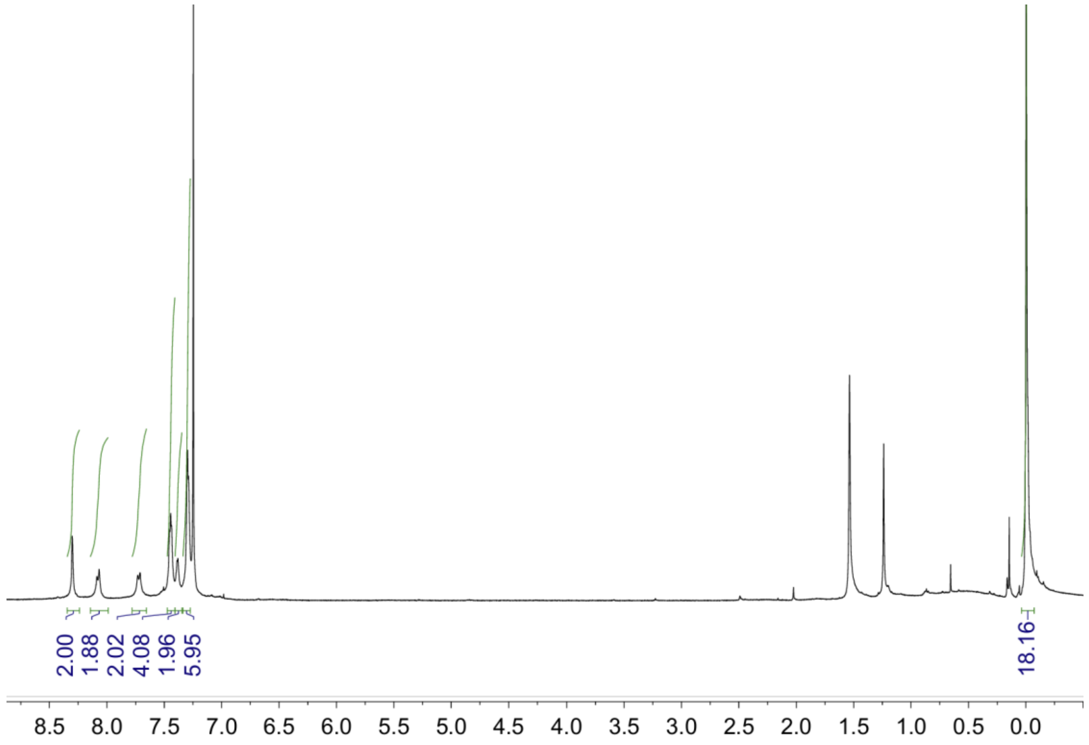

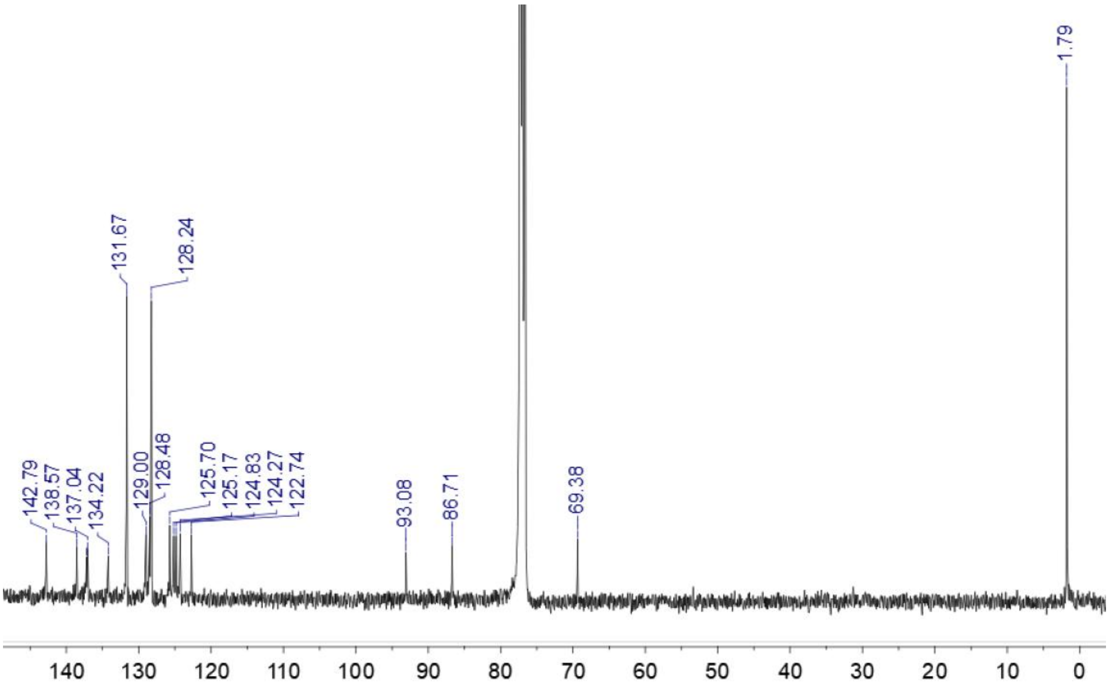

HA(TIPS)(OTMS)-BT: The polymer was obtained as a yellow/green powder (77% yield). ^{1}H-NMR (400 MHz, $CDCl_3$) δ (ppm): 8.26 (s, 2H), 8.01 (d, J = 8.2 Hz, 2H), 7.71 (d, J = 8.4 Hz, 2H), 7.41 (d, J = 3.7 Hz, 2H), 7.27 (s, 2H), 1.10 (s, 42H), -0.22 (s, 18H). ^{13}C-NMR (101 MHz, Chloroform-d) δ (ppm): 142.69, 137.49, 136.98, 136.09, 134.02, 129.98, 126.06, 125.57, 124.6, 124.06, 111.96, 86.43, 68.40, 18.69, 11.37, 1.51. Elem. Anal. $C_{50}H_{70}O_2S_2Si_4$. Calc. (%) C 68.28, H 8.02, O 3.64, S 7.29, Si 12.77. Exp. (%) C 67.81, H 7.780, S 7.350.

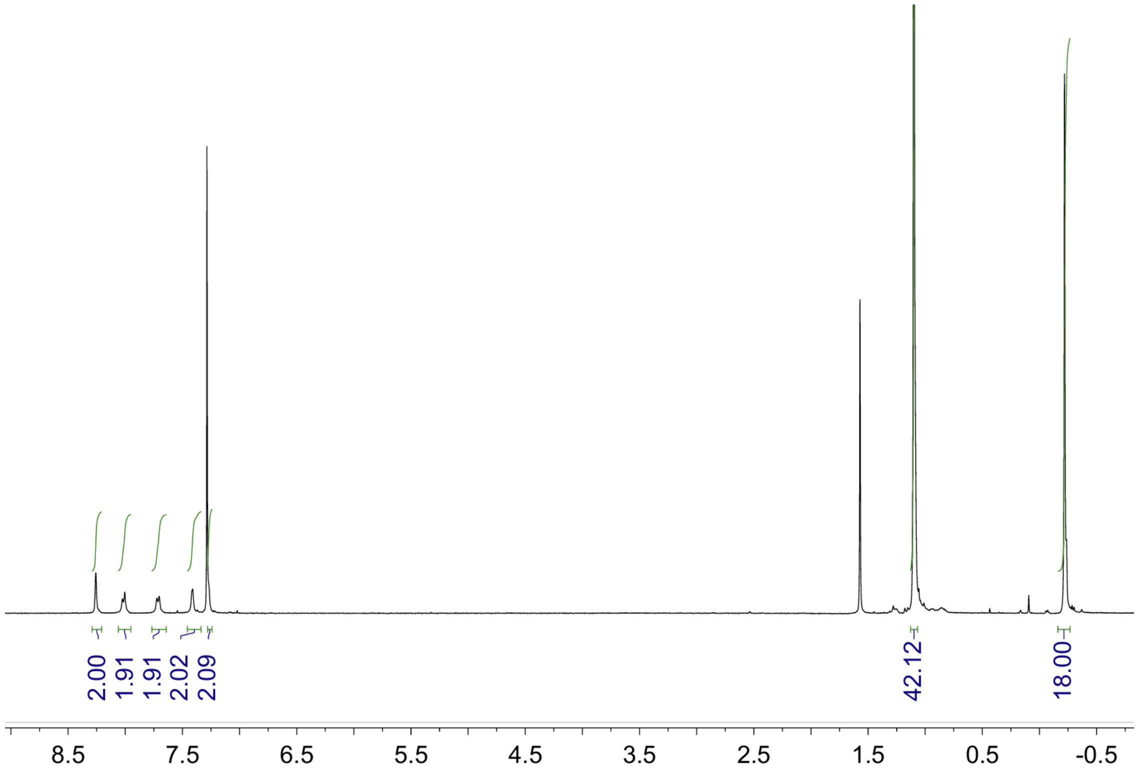

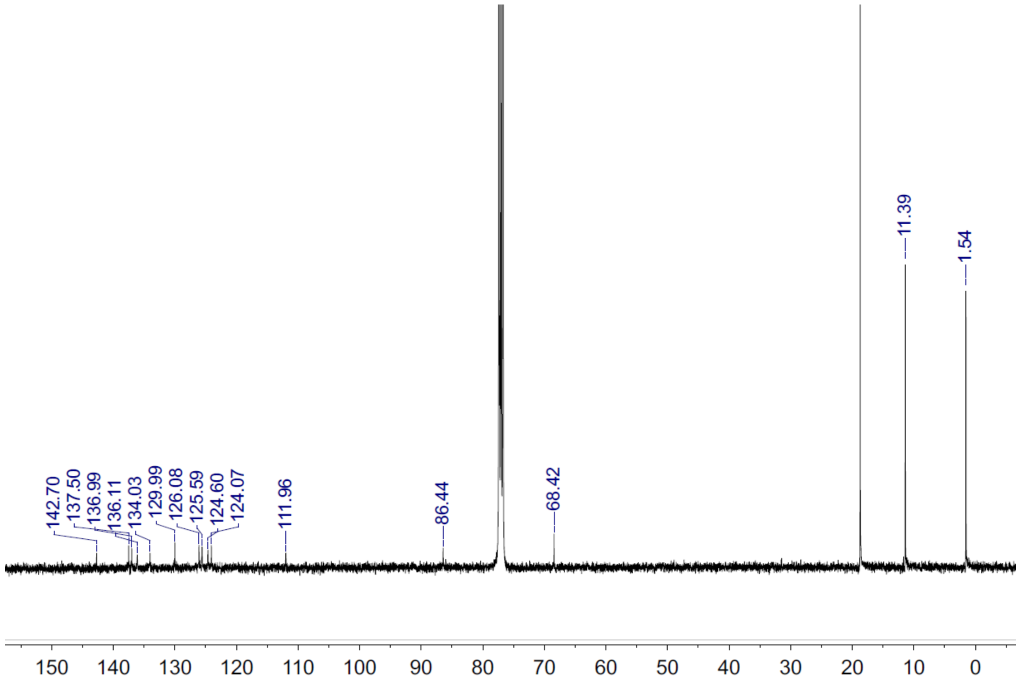

HA(TIPS)(OMe)-BT: The polymer was obtained as an orange powder (85% yield). ^{1}H-NMR (400 MHz, $CDCl_3$) δ (ppm): 8.28 (s, 2H), 8.04 (d, J = 8.0 Hz, 2H), 7.77 (d, J = 8.1 Hz, 2H), 7.43 (s, 2H), 7.28 (s, 2H) 2.88 (s, 6H), 1.09 (s, 42H). Elem. Anal. $C_{46}H_{58}O_2S_2Si_2$. Calc. (%) C 72.39, H 7.66, O 4.19, S 8.40, Si 7.36. Exp. (%) C 72.08, H 8.013, S 8.482.

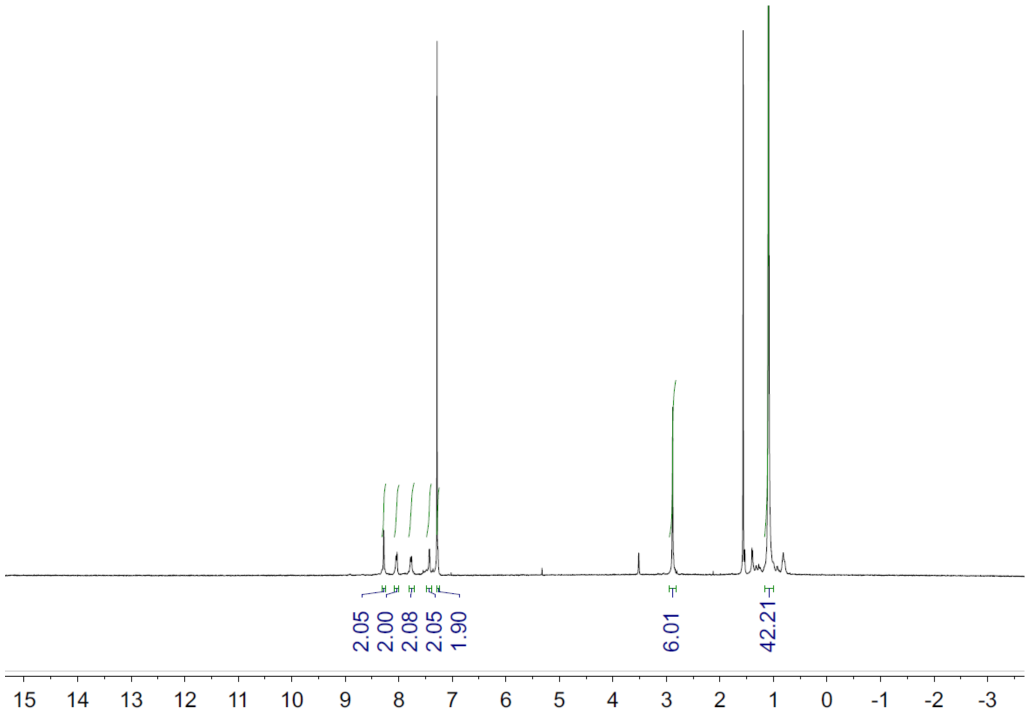

HA(Hex)(OTMS)-BT: The polymer was obtained as a yellow powder (73% yield). ^{1}H-NMR (400 MHz, $CDCl_3$) δ (ppm): 8.18 (s, 2H), 7.95 (d, J = 7.8 Hz, 2H), 7.66 (d, J = 8.1 Hz, 2H), 7.36 (s, 2H), 2.30-2.20 (m, 4H), 1.50-1.45 (m, 4H), 1.37-1.28 (m, 4H), 1.24-1.19 (m, 8 H), 0.82-0.79 (m, 6H), -0.09 (s, 18H). ^{13}C-NMR (101 MHz, $CDCl_3$) δ (ppm): 142.93, 138.60, 137.31, 136.93, 133.85, 129.09, 125.41, 125.39, 124.67, 124.66, 124.06, 123.56, 87.56, 84.66, 68.82, 31.34, 28.65, 28.37, 22.53, 19.13, 14.03, 1.71. Elem. Anal. $C_{44}H_{54}O_2S_2$. Calc. (%) C 71.88, H 7.40, O 4.35, S 8.72, Si 7.64. Exp. (%) C 71.84, H 7.525, S 8.651.

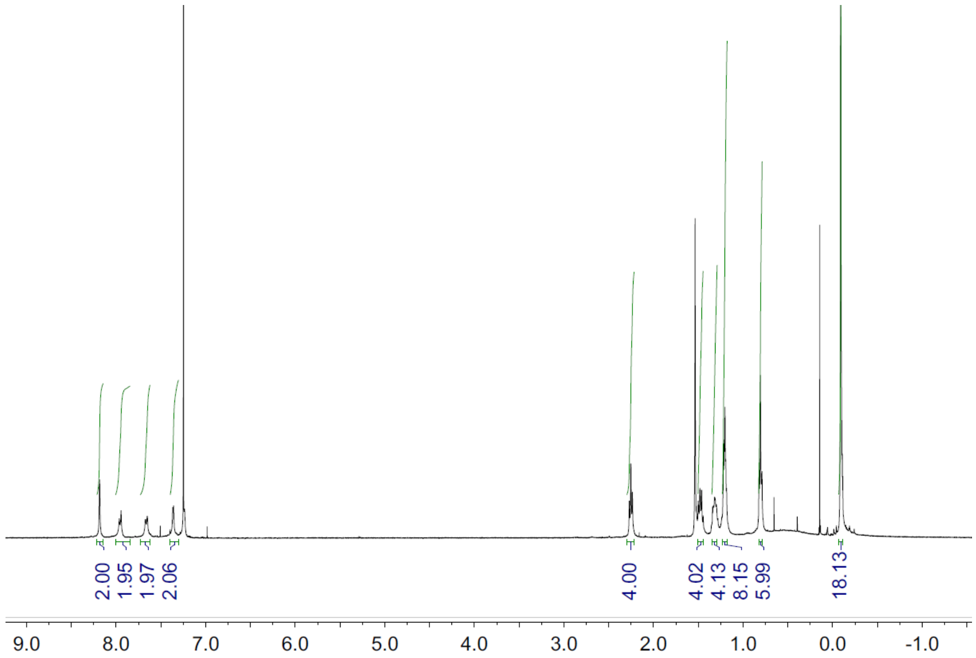

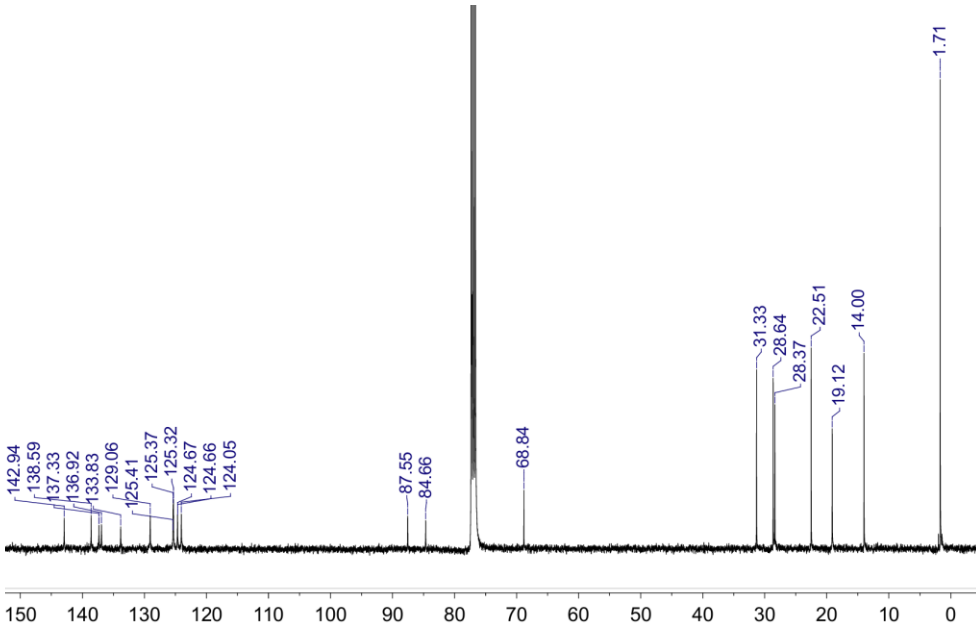

HA(Ph)(OTMS)-TT: The polymer was obtained as a red powder (67% yield). ^{1}H-NMR (400 MHz, $CDCl_3$) δ (ppm): 8.29 (s, 2H), 8.08-8.06 (d, J = 8.1 Hz, 2H), 7.72-7.70 (d, J = 7.7 Hz, 2H), 7.47-7.41 (m, 4H), 7.39-7.37 (d, J = 7.5 Hz, 2H), 7.32-7.27 (m, 6 H), 7.21-7-20 (d, J = 7.2 Hz, 2H), 7.16 (s, 2H), -0.01 (s, 18H). Elem. Anal. $C_{48}H_{42}O_2S_3Si_2$. Calc. (%) C 71.78, H 5.27, O 3.98, S 11.97, Si 6.99. Exp. (%) C 71.26, H 5.38, S 11.81.

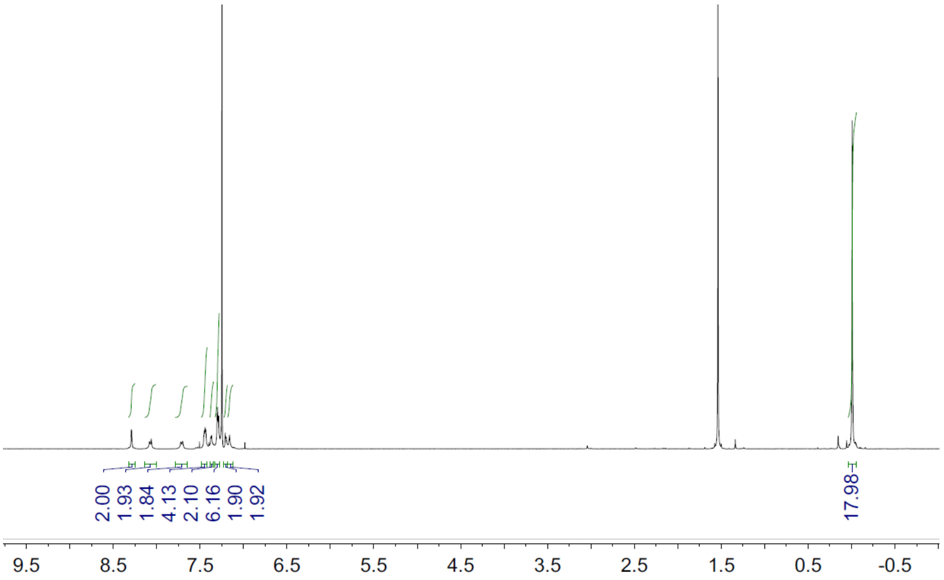

HA(Ph)(OMe)-TT: The polymer was obtained as a red powder (73% yield). ^{1}H-NMR (400 MHz, $CDCl_3$) δ (ppm): 8.3 (s, 2H), 8.09 (d, J = 8.0 Hz, 2H), 7.77 (d, J = 8.1 Hz, 2H), 7.48-7.27 (m, 12 H), 7.22-7.13 (m, 4H), 3.01 (s, 6H). Elem. Anal. $C_{44}H_{30}O_2S_3$. Calc. (%) C 76.94, H 4.40, O 4.66, S 14.00. Exp. (%) C 77.09, H 4.49, S 14.02.

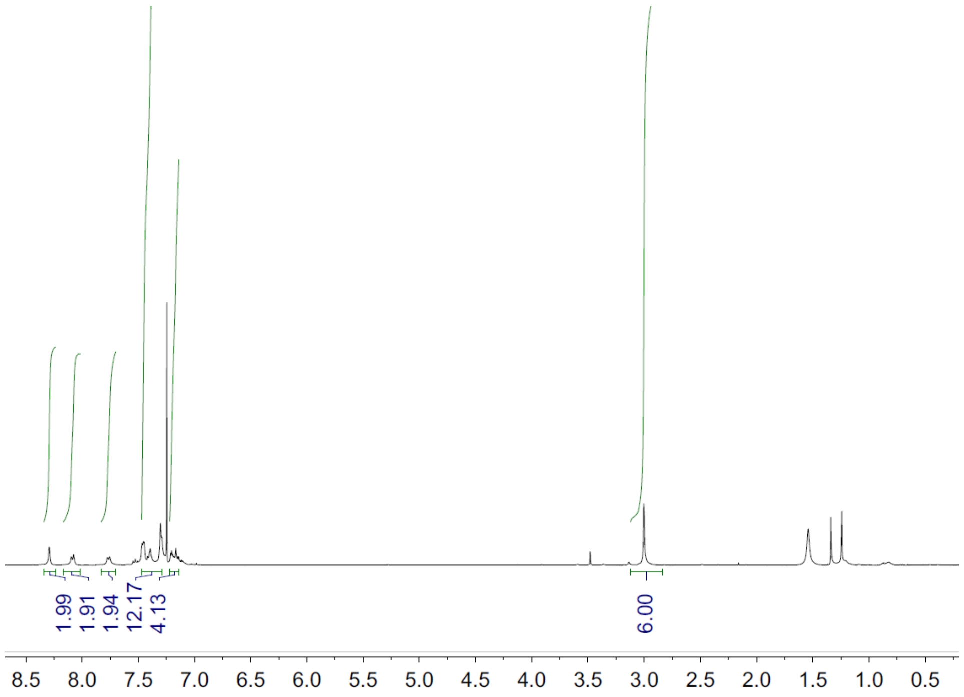

HA(TIPS)(ODMBS)-BT: The polymer was obtained as a yellow powder (86% yield). ^{1}H NMR (400 MHz, $CDCl_3$) δ (ppm): 8.26 (s, 2H), 8.01 (d, J = 7.9 Hz, 2H), 7.71 (d, J = 7.2 Hz, 2H), 7.40 (s, 2H), 7.27-7.20 (m, 2H), 1.28-1.16 (m, 8H), 1.11 (s, 42H), 0.87-0.78 (m, 6H), 0.39-0.29 (m, 4H), -0.34 (s, 6H), -0.37 (s, 6H). Elem. Anal. $C_{48}H_{70}O_2S_2Si_4$. Calc. (%) C 67.39, H 8.25, O 3.74, S 7.49, Si 13.13. Exp. (%) C 66.88, H 8.990, S 7.330.

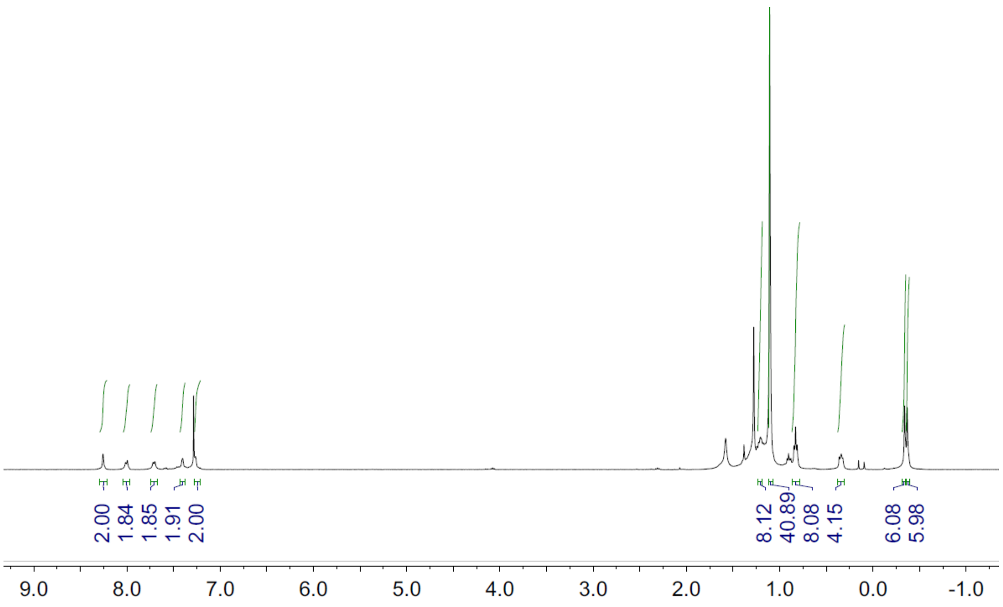

HA(TIPS)(OTMS)-NDI: The synthesis of the monomers and of this polymer are described elsewhere.[S4] The polymer was obtained as a purple powder (82% yield). ^{1}H-NMR (400 MHz, $CDCl_3$) δ (ppm): 8.22 (d, J = 1.9 Hz, 2H), 7.95 (d, J = 8.4 Hz, 2H), 7.67 (dd, J = 8.4 Hz, J = 1.9 Hz, 2H), 7.43 (dd, J = 3.7 Hz, J = 1.1 Hz, 2H), 7.32 (dd, J = 5 Hz, J = 1.1 Hz, 2H), 7.12 (dd, J = 5 Hz, J= 3.7 Hz, 2H), 1.03 (s, 42H), -0.27 (s, 18H). Elem. Anal. $C_{80}H_{106}N_2O_6S_2Si_4$. Calc. (%) C 70.23, H 7.81, N 2.05, O 7.02, S 4.69, Si 8.21. Exp. (%) C 70.98, H 7.654, N 2.13, S 4.97.

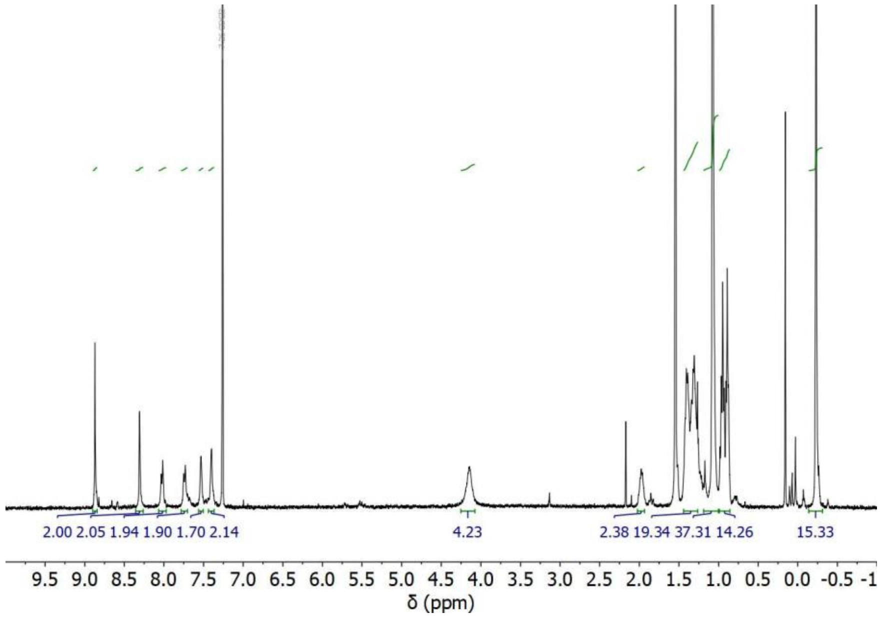

BP(TIPS)(OTMS)-T: The polymer was obtained as a green glassy solid (28% yield). ^{1}H-NMR (400 MHz, $CDCl_3$) δ (ppm): 7.64-7.49 (m, 7H), 7.22 (s, 2H), 1.12 (s, 21H), 0.15 (s, 9 H). Elem. Anal. $C_{31}H_{40}OSSi_2$. Calc. (%) C 72.03, H 7.80, O 3.10, S 6.20, Si 10.87. Exp. (%) C 72.14, H 7.512, S 5.972.

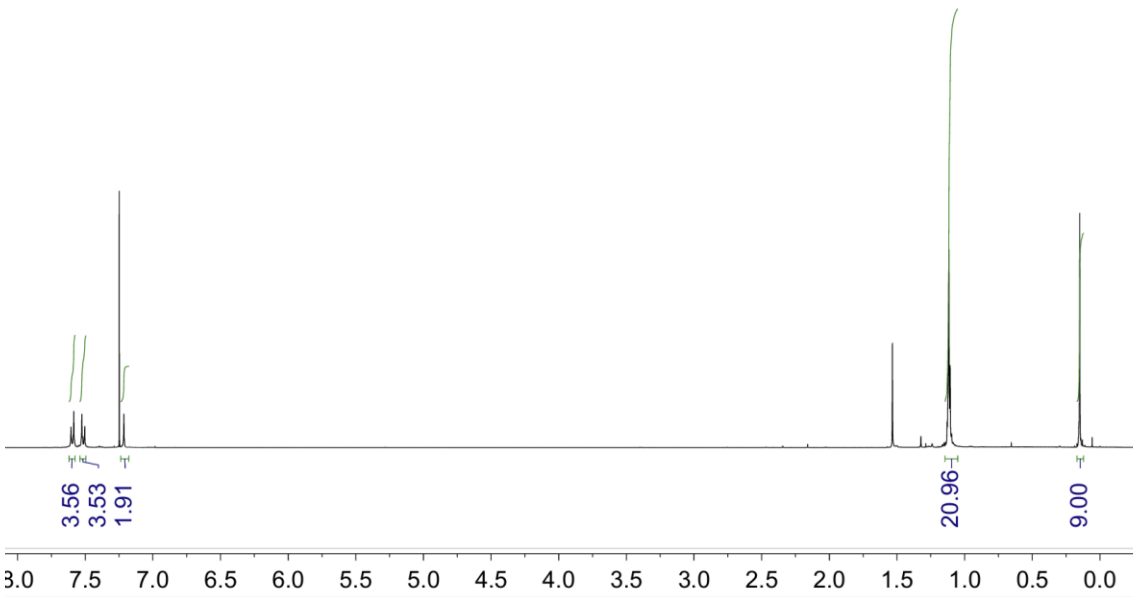

FL(Ph)(OTMS)-T: the polymer was obtained as a brown solid (65% yield). ^{1}H-NMR (400 MHz, $CDCl_3$) δ (ppm): 8.02 (s, 2 H), 7.74-7.59 (m, 4H), 7.46-7.40 (m, 5H), 0.10-0.03 (m, 9H). Elem. Anal. $C_{28}H_{22}OSSi$. Calc. (%) C 77.38, H 5.10, O 3.68, S 7.38, Si 6.46. Exp. (%) C 74.87, H 5.340, S 7.990.

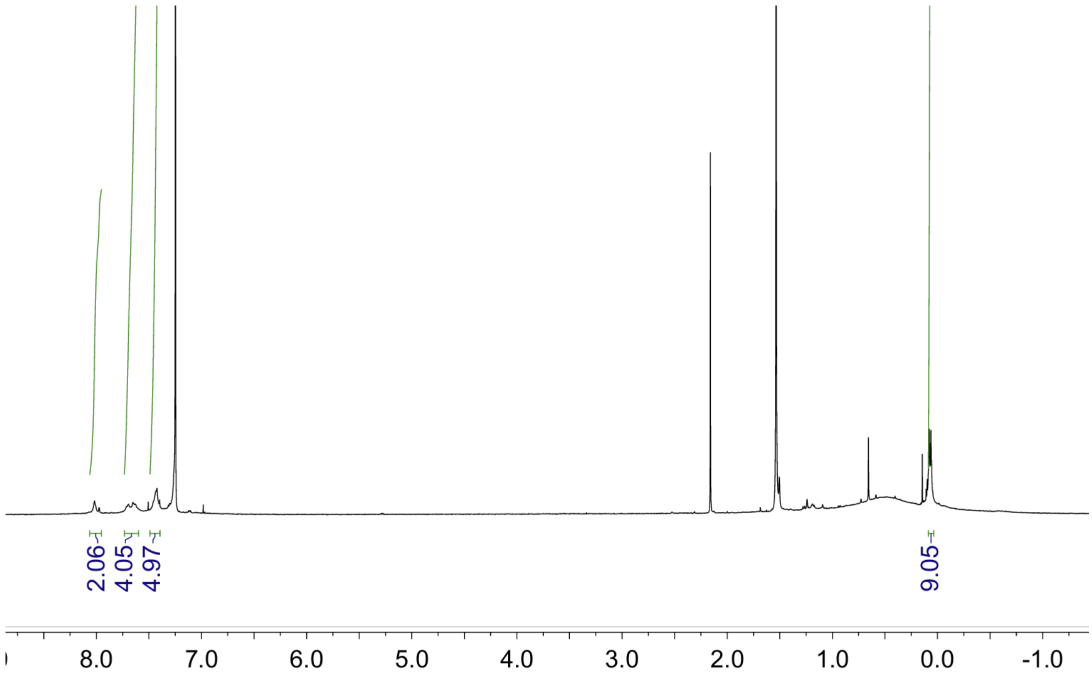

Synthesis of HA(Ph)(OMe)-Ph

DiBrAH(Ph)(OMe), 1,4-dioxane, 1,4-phenylenediboronic acid, and K_2CO_3 were inserted in a Schenk flask. The mixture was degassed for 30 min with N_2, then XPhosPdG2 was added. The reaction was kept at 105 °C for 2 h. Then, 1-bromo-4-(tert-butyl)benzene was added, and the mixture stirred for 30 min. The mixture was then treated with a satured aqueous solution of NH_4Cl and poured in MeOH. The precipitate was filtered and extracted with boiling acetone. It was then dissolved in chloroform and reprecipitated in methanol to afford the polymer as a green powder (51% yield). ^{1}H-NMR (400 MHz, $CDCl_3$) δ (ppm): 8.39 (s, 1H), 8.18 (d, J = 8 Hz, 1H), 7.86 (s, 4H), 7.47 (s, 4 H), 7.29 (d, J = 3.9 Hz, 9 H), 7.27 (1 H, under solvent signal), 3.04 (s, 6H). Elem Anal. $C_{38}H_{26}O_2$. Calc. (%): C 88.69, H 5.09, O 6.22. Exp (%) C 88.42, H 4.955.

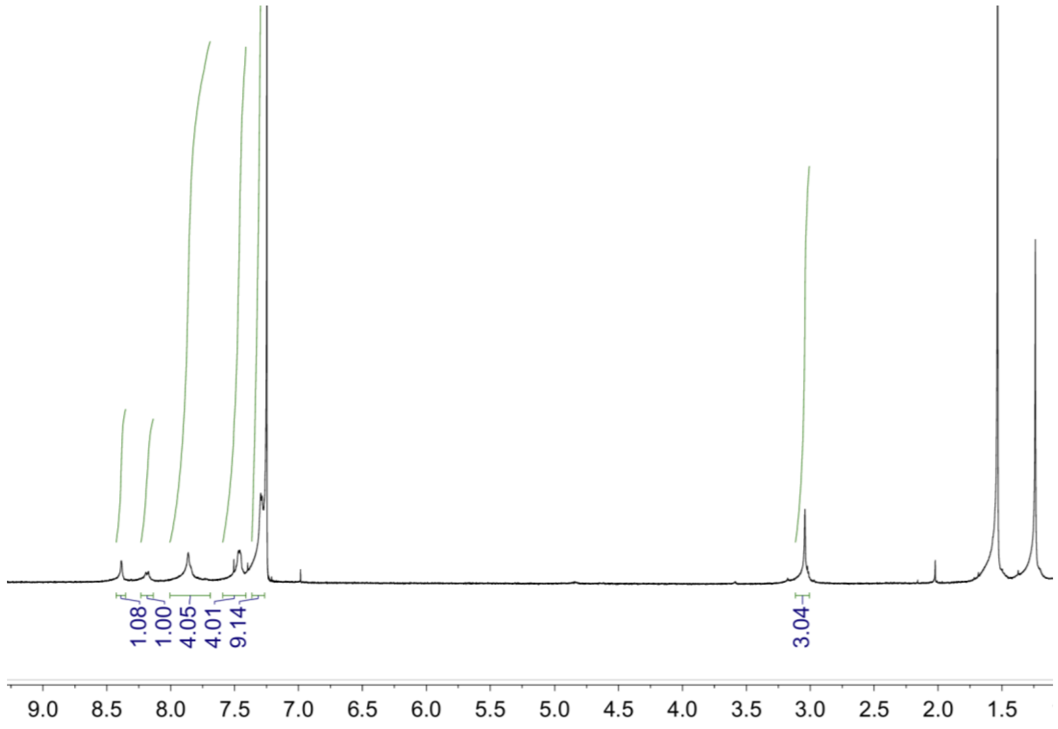

Table S1. Mn, Mw, PDI, degree of polymerization (Xn; calculated from Mn), and polydispersity index of HA-based precursors not reported in Table 1 of the main text.

Polymer	R1	R2	Linker	Yield [%]	Mn	Mw	PDI	Xn
HA(TIPS)(ODMBS)-BT	TIPS	DMBS	Bithiophene	86	10363	19052	1.87	10.8
HA(TIPS)(OTMS)-NDI	TIPS	TMS	N,N'-bis(2-ethyl(hexyl)-2,6-thiopheno-napthalenetetracarboxybisimmide	82	32165	50943	1.58	25.0
FL(Ph)(OTMS)-T	Phenyl	TMS	Thiophene	65	2354	4184	1.78	5.4
BP(TIPS)(OTMS)-T	TIPS	TMS	Thiophene	28	14390	22909	1.59	27.8
HA(Ph)(OMe)-Ph	Phenyl	Methoxy	Phenyl	51	18536	87140	4.70	36.0
BQ(Ph)(OTMS)-BT	Phenyl	TMS	Bithiophene	35	15645	21074	1.38	25.3

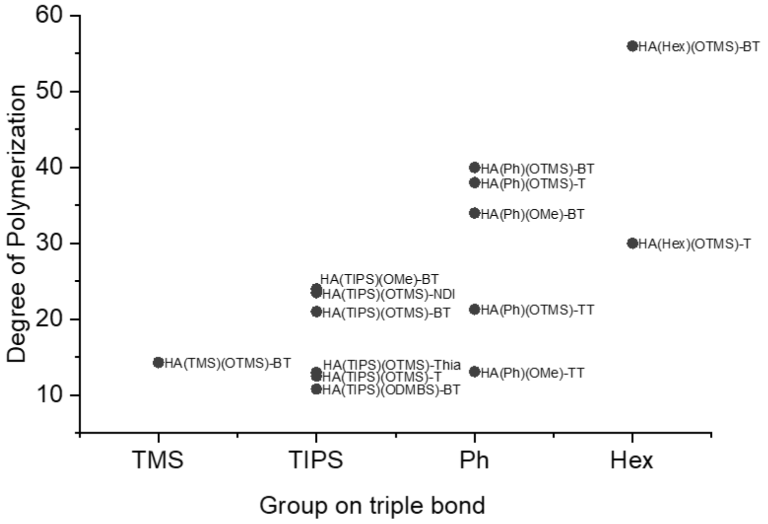

Figure S1. Influence of the substituent on the triple bond on the degree of polymerization.

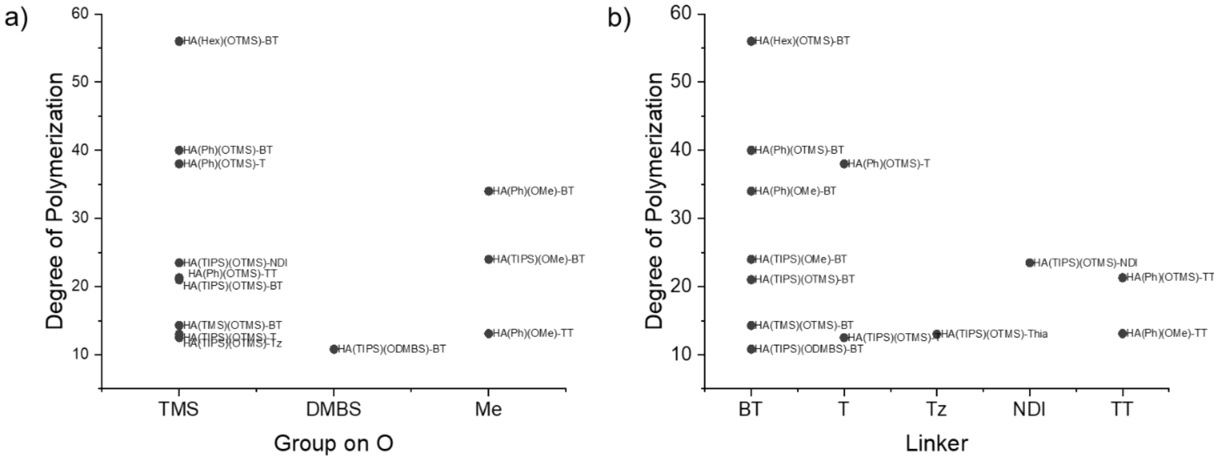

Figure S2. Influence of the O-substitution (a) and the linker nature (b) on the degree of polymerization.

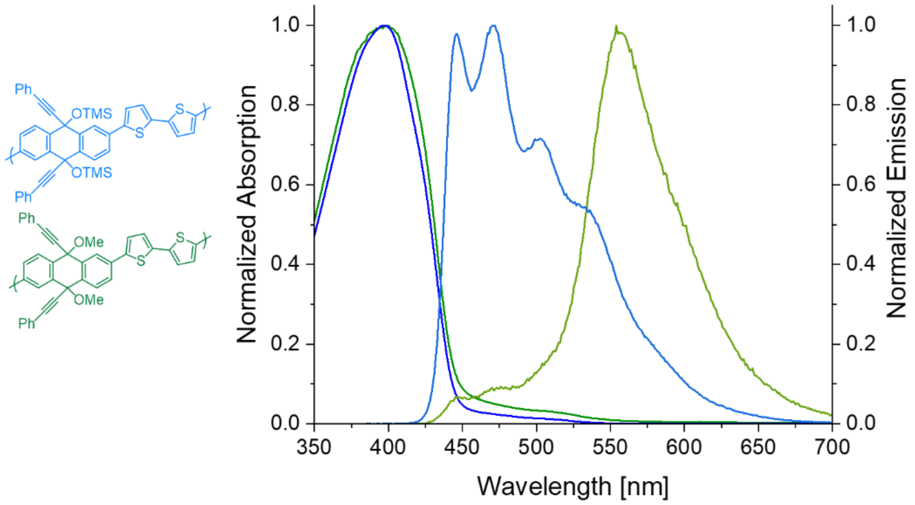

Figure S3. Normalized absorption and emission spectra of **HA(Ph)(OTMS)-BT** and **HA(Ph)(OMe)-BT** in chloroform solution.

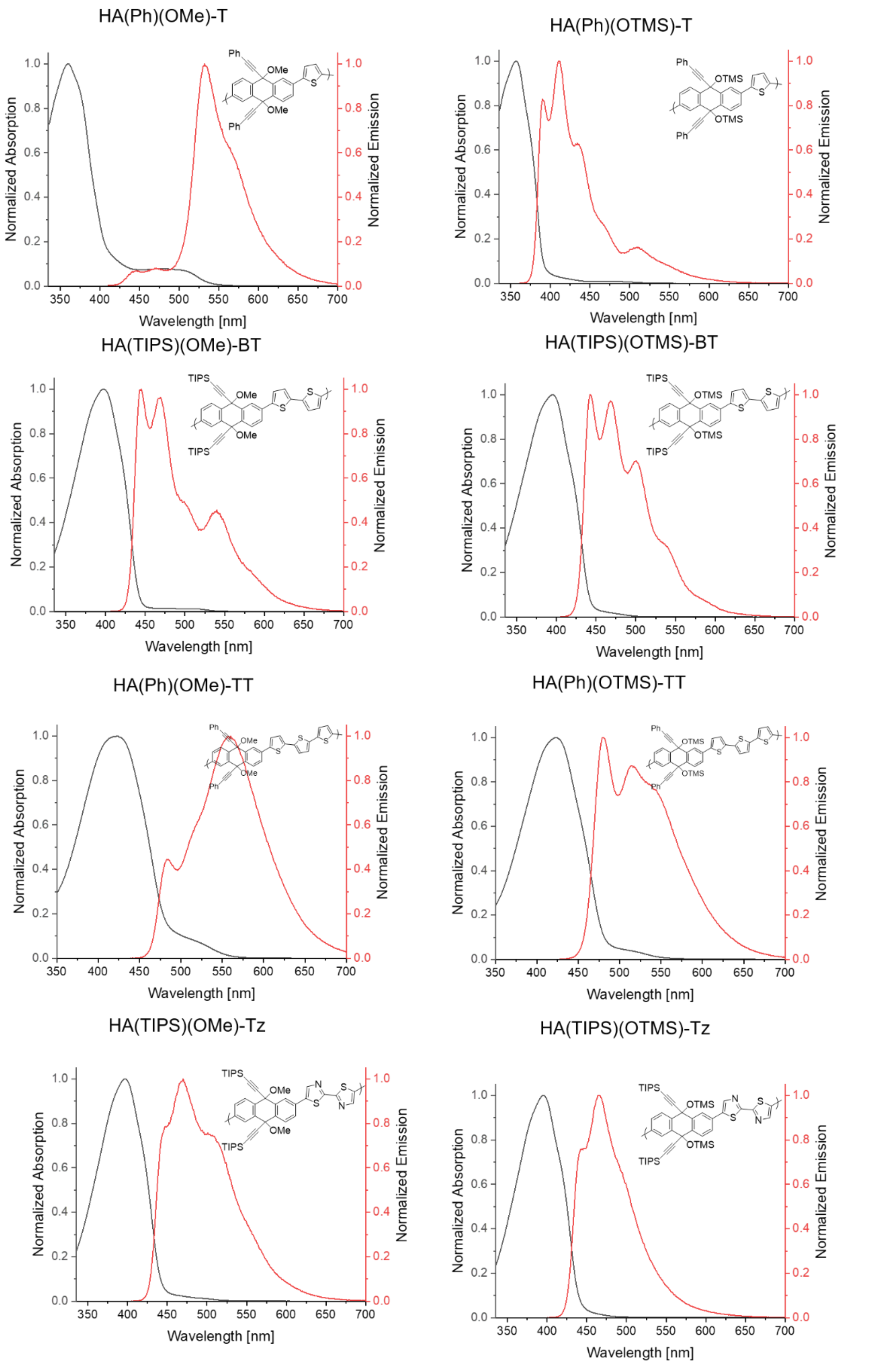

Figure S4. Normalized absorption and emission spectra of various HA-based polymeric precursors in chloroform solution.

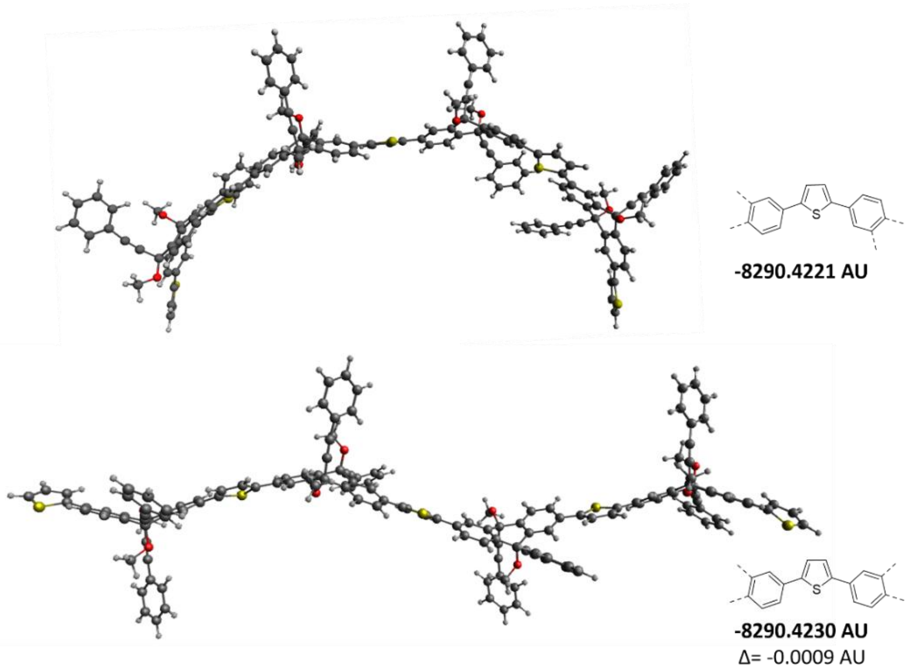

Figure S5. DFT-computed global minimum potential energy conformations for the tetramer of **HA(Ph)(OMe)-T**. Single point energies are reported in atomic units (Hartree).

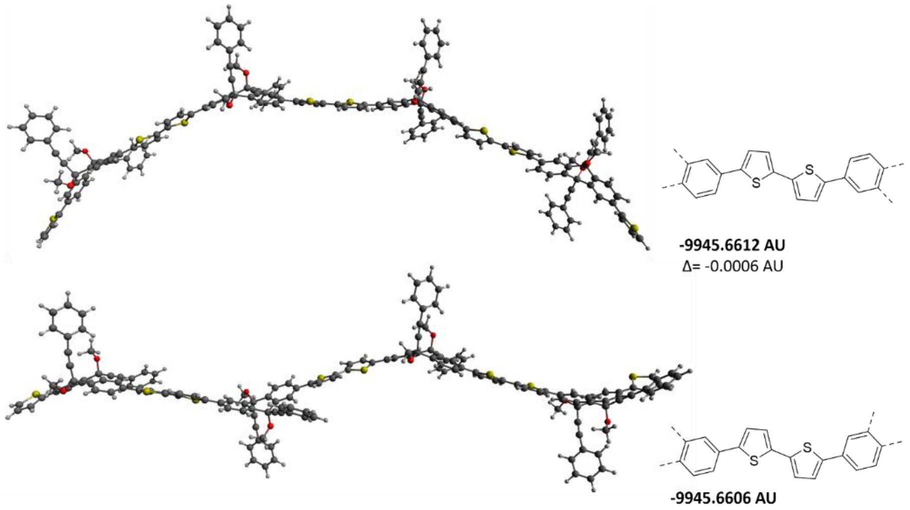

Figure S6. DFT-computed global minimum potential energy conformations for the tetramer of **HA(Ph)(OMe)-BT**. Single point energies are reported in atomic units (Hartree).

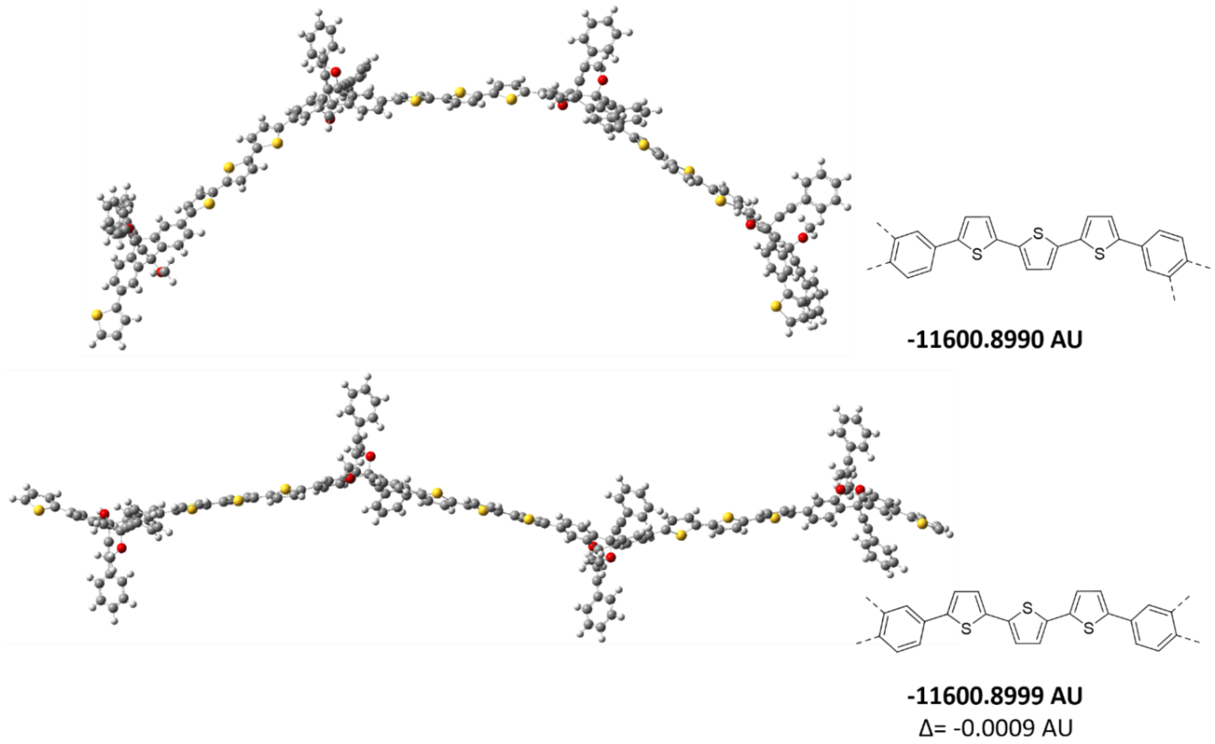

Figure S7. DFT-computed global minimum potential energy conformations for the tetramer of **HA(Ph)(OMe)-TT**. Single point energies are reported in atomic units (Hartree).

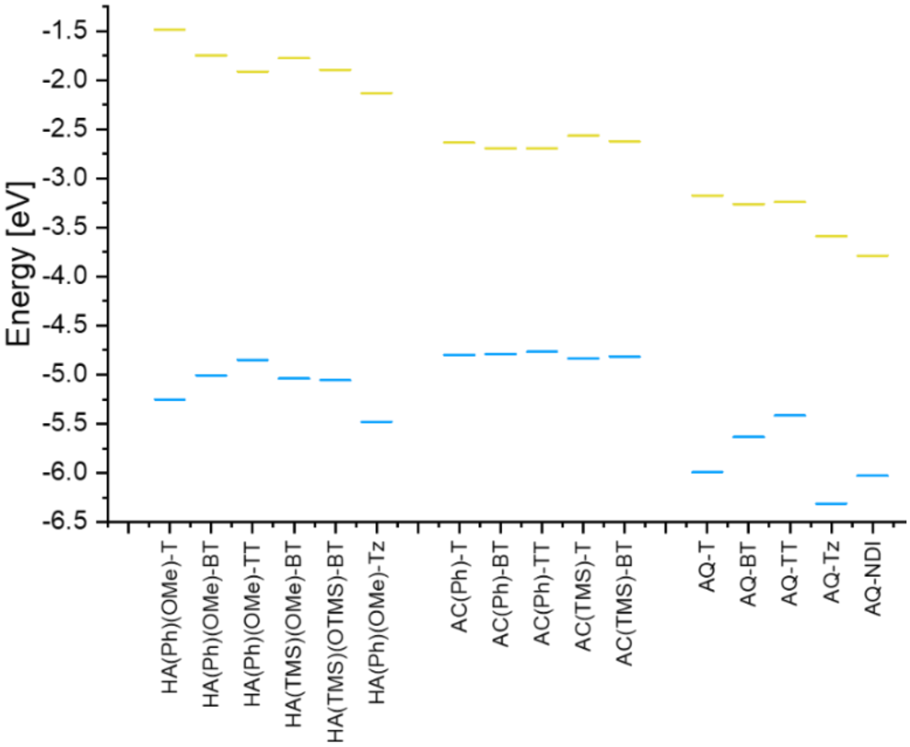

Figure S8. DFT-computed HOMO and LUMO energy levels for three different sets of HA-based polymeric precursors, AC-based fully conjugated polymers, and AQ-based fully conjugated polymers.

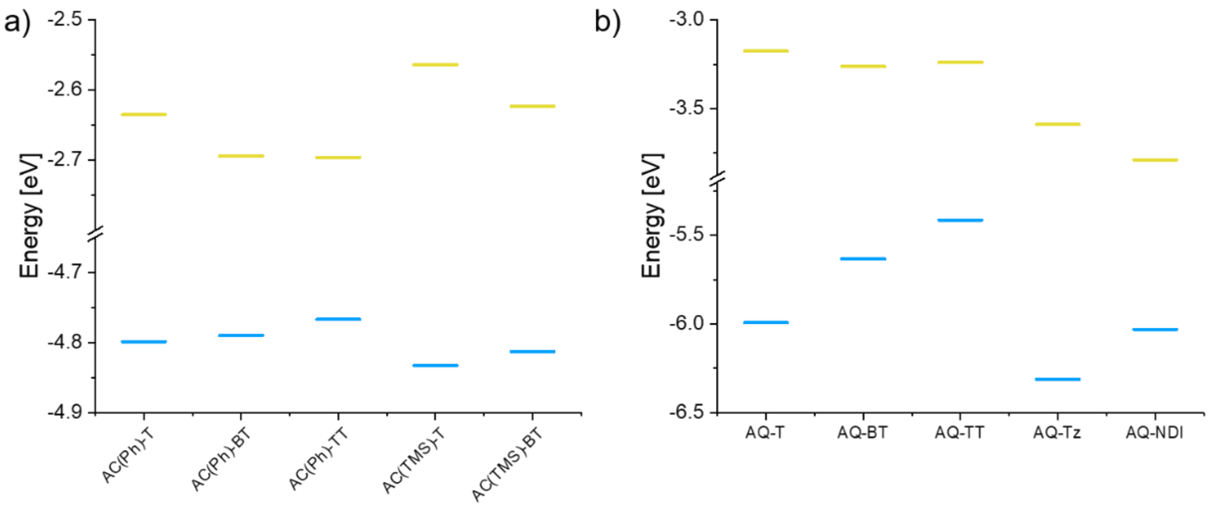

Figure S9. DFT-computed HOMO and LUMO energy levels for two different sets of conjugated polymers: a) AC-based, b) AQ-based.

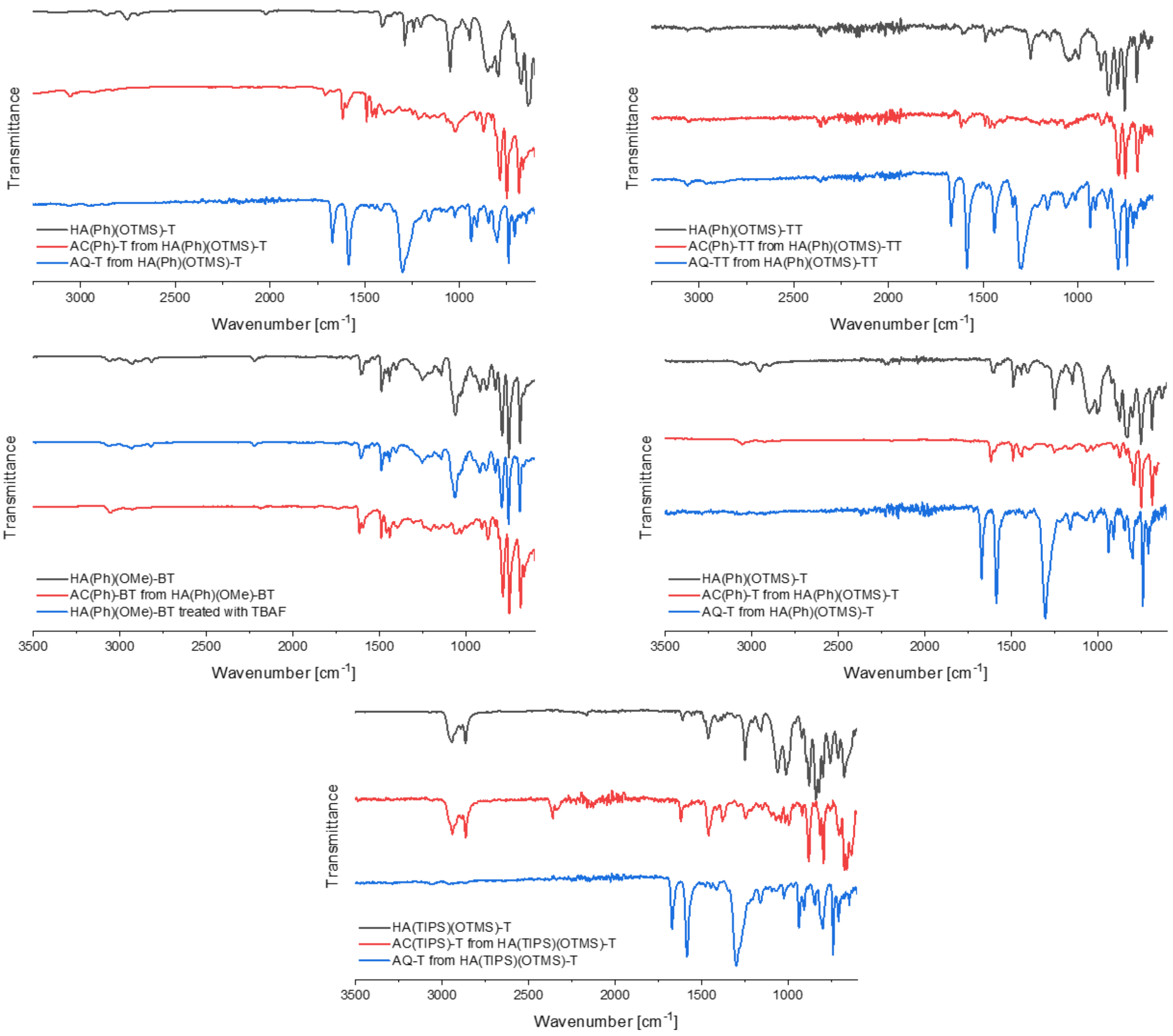

Figure S10. IR spectra comparison between HA-based polymeric precursor, AC-based fully conjugated polymer, and AQ-based fully conjugated polymer for five different sets of triple bond/oxygen substitutions and linker nature of the precursor.

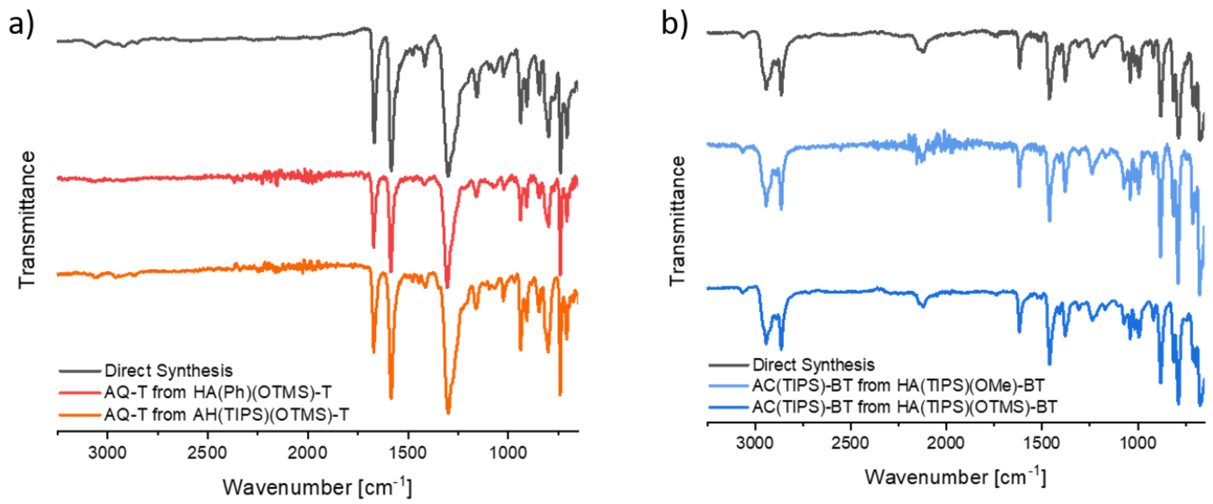

Figure S11. IR spectra comparison between AQ-based (a) and AC-based (b) fully conjugated polymers obtained via direct synthesis and/or HA-based polymeric precursors.

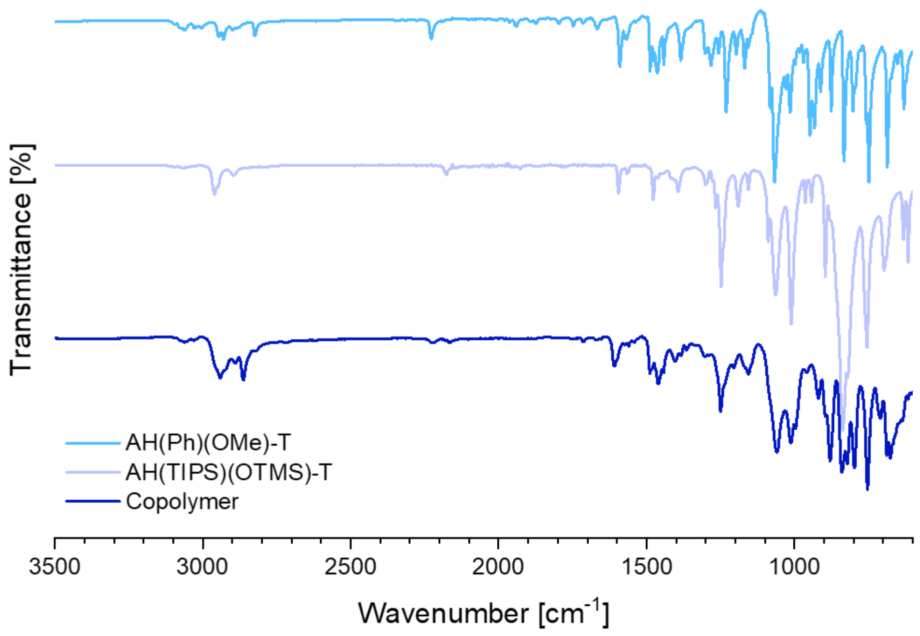

Figure S12. IR spectra comparison between **HA(Ph)(OMe)-T**, **HA(TIPS)(OTMS)-T** and random copolymer between the two different HA-based units with the thiophene linker.

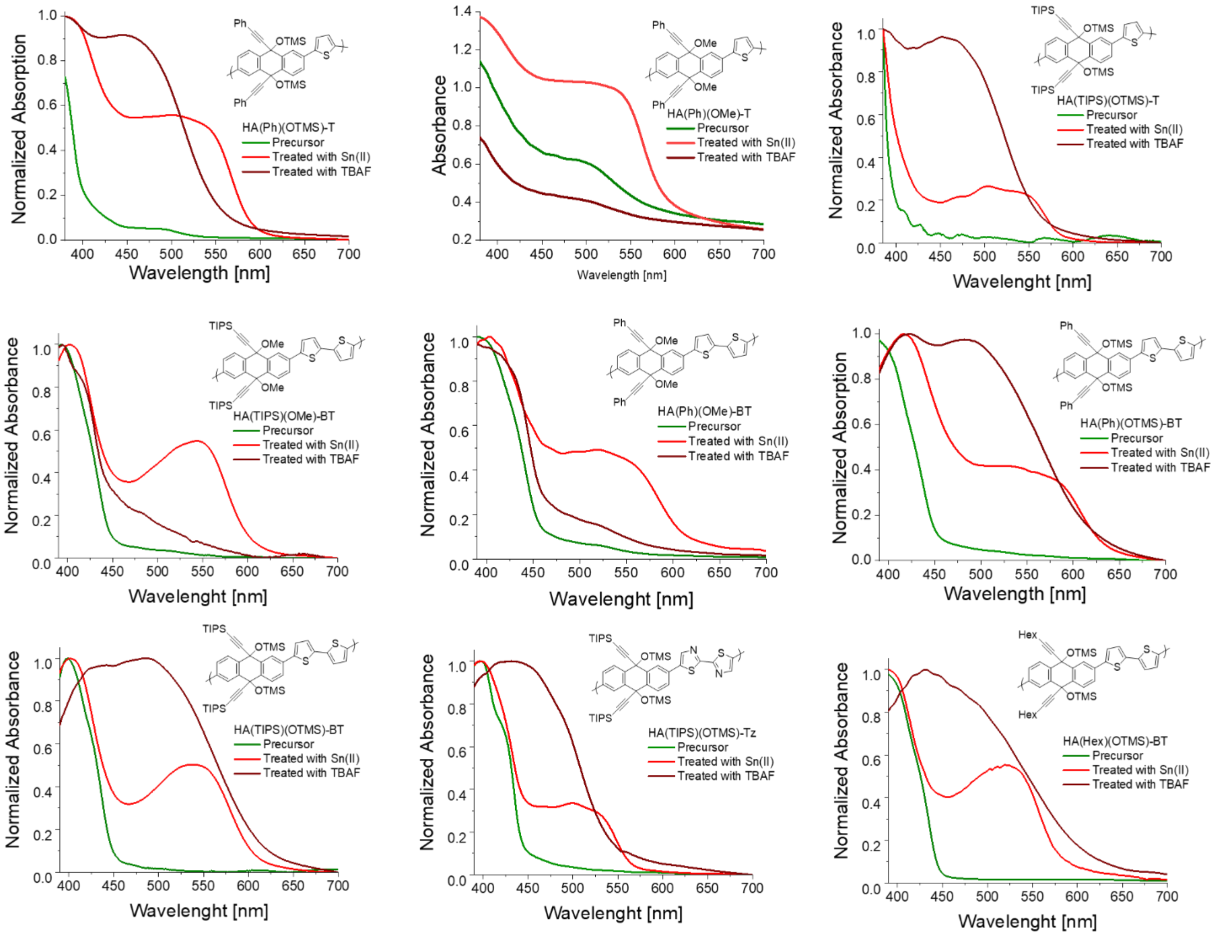

Figure S13. Comparison of normalized absorption spectra of thin films of HA-based polymeric precursor, and after treatment with either Sn^{II}, to perform the reduction-rearomatization, or TBAF. Note that the presence of OMe functionality does not allow the Retro-Favorskii reaction to occur.

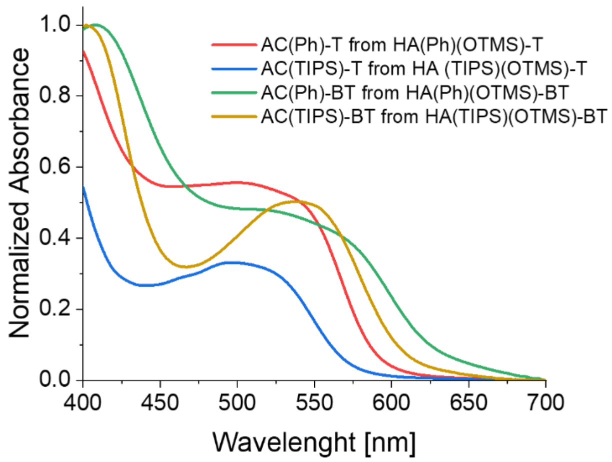

Figure S14. Normalized absorption spectra of films comprising AC-based fully conjugated polymers and different substitutions on the triple bond and linkers.

Table S2. Elemental analysis of conjugated polymers obtained from the HA-precursors. Between brackets, we reported the calculated values for the theoretical structures.

Polymer	C [%]	H [%]	N [%]	S [%]	C/S	N/S
**AC(Ph)-T $C_{34}H_{18}S$**	**(89.05)**	**(3.96)**	**(0)**	**(6.99)**	**12.7**	**-**
From HA(Ph)(OTMS)-T	81.06	3.621	0.01	6.232	13.0	-
From HA(Ph)(OMe)-T	87.23	3.883	0	6.740	12.9	-
**AC(TIPS)-T $C_{40}H_{50}SSi_2$**	**(77.61)**	**(8.14)**	**(0)**	**(5.18)**	**15.0**	**-**
From HA(TIPS)(OTMS)-T	78.03	8.219	0	5.104	15.3	-
**AC(Ph)-BT $C_{38}H_{20}S_2$**	**(84.41)**	**(3.73)**	**(0)**	**(11.86)**	**7.1**	**-**
From HA(Ph)(OMe)-BT	82.939	3.45	0	11.182	7.4	-
From HA(Ph)(OTMS)-BT	81.44	3.932	0.08	11.034	7.4	-
**AC(TIPS)-BT $C_{44}H_{52}S_2Si_2$**	**(75.37)**	**(7.48)**	**(0)**	**(9.14)**	**8.2**	**-**
From HA(TIPS)(OTMS)-BT	72.11	7.284	0	8.716	8.3	-
From HA(TIPS)(OMe)-BT	74.74	7.730	0	8.985	8.3	-
**AC(Ph)-TT $C_{42}H_{22}S_3$**	**(81.00)**	**(3.56)**	**(0)**	**(15.44)**	**5.3**	**-**
HA(Ph)(OMe)-TT	76.94	3.210	0	14.364	5.4	-
HA(Ph)(OTMS)-TT	79.66	3.691	0	15.179	5.2	-
**AC(Ph)-Tz $C_{36}H_{18}N_2S_2$**	**(79.68)**	**(3.34)**	**(5.16)**	**(11.82)**	**6.7**	**0.44**
HA(Ph)(OTMS)-Tz	76.51	3.227	4.93	11.28	6.8	0.44
**AC(TIPS)-Tz $C_{42}H_{50}N_2S_2Si_2$**	**(71.64)**	**(7.17)**	**(3.98)**	**(9.12)**	**7.9**	**0.44**
HA(TIPS)(OTMS)-Tz	68.96	7.135	3.81	8.634	8.0	0.44
**AQ-T $C_{18}H_8O_2S$**	**(74.99)**	**(2.80)**	**(0)**	**(11.12)**	**6.7**	**-**
From HA(Ph)(OTMS)-T	76.55	2.912	0.15	10.597	7.2	-
From HA(TIPS)(OTMS)-T	78.78	3.015	0.09	11.05	7.1	-
**AQ-BT $C_{22}H_{10}O_2S_2$**	**(71.33)**	**(2.72)**	**(0)**	**(17.31)**	**4.2**	**-**
From HA(Ph)(OTMS)-BT	68.61	3.037	0.16	15.599	4.4	-
From HA(TIPS)(OTMS)-BT	69.66	2.939	0.05	16.423	4.2	-
From HA(TIPS)(ODMBS)-BT	71.12	3.112	0.08	16.653	4.3	-
From HA(Hex)(OTMS)-BT	70.45	3.057	0.07	16.537	4.3	-
From HA(TMS)(OTMS)-BT	70.17	2.845	0.09	16.492	4.3	-
**AQ-TT $C_{26}H_{12}O_2S_3$**	**(69.00)**	**(2.67)**	**(0)**	**(21.25)**	**3.2**	**-**
From HA(Ph)(OTMS)-TT	67.78	2.76	0.05	19.647	3.4	-
**AQ-Tz $C_{20}H_8N_2O_2S_2$**	**(64.50)**	**(2.17)**	**(7.52)**	**(17.22)**	**3.7**	**-**
From HA(Ph)(OTMS)-Tz	63.11	2.098	7.07	16.147	3.9	0.44
From HA(TIPS)(OTMS)-Tz	62.98	2.040	7.12	16.355	3.9	0.44
**AQ-NDI $C_{52}H_{46}N_2O_6S_2$**	**(72.60)**	**(5.40)**	**(3.26)**	**(7.46)**	**9.7**	**0.44**
From HA(TIPS)(OTMS)-NDI	71.54	5.233	3.21	7.25	9.9	0.44

Table S3. Elemental percentages determined via XPS of a series of conjugated polymers obtained from HA-precursors. Calculated values are reported in brackets and were calculated from the molecular formulas reported in the first column. Major contaminants are reported in the column ‘Other’.

Polymer	C [%]	S [%]	O [%]	Si [%]	Other [%]
**AC(Ph)-T** $C_{34}S_1$	**(92.7)**	**(7.28)**	**(0)**	**(0)**	-
From HA(Ph)(OTMS)-T	80.5	2.83	9.9	5.83	Sn 0.94
**AC(Ph)-BT** $C_{38}S_2$	**(87.68)**	**(12.32)**	**(0)**	**(0)**	-
From HA(Ph)(OTMS)-BT	77.85	2.08	15.3	4.08	-
**AC(Ph)-TT** $C_{42}S_3$	**(83.99)**	**(16.01)**	**(0)**	**(0)**	-
From HA(Ph)(OTMS)-TT	79.87	4.61	13.13	1.66	Sn 0.63
**AC(TIPS)-BT** $C_{44}S_2Si_2$	**(91.6)**	**(4.2)**	**(0)**	**(4.2)**	-
From HA(TIPS)(OTMS)-BT[a]	87.9	3.4	3.9	4.8	Sn
**AQ-T** $C_{18}O_2S_1$	**(77.14)**	**(11.44)**	**(11.42)**	**(0)**	-
From HA(Ph)(OTMS)-T	77.92	2.66	16.55	0	-
**AQ-BT** $C_{22}O_2S_2$	**(73.33)**	**(17.79)**	**(8.88)**	**(0)**	-
From HA(Ph)(OTMS)-BT	81.68	6.73	11.6	0	-
From HA(TIPS)(OTMS)-BT[a]	86.5	5.9	7.3	0.3	-
**AQ-TT** $C_{26}O_2S_3$	**(70.9)**	**(21.84)**	**(7.26)**	**(0)**	-
From HA(Ph)(OTMS)-TT	71.86	4.08	17.93	6.13	-

[a] Measured in ref. [26]

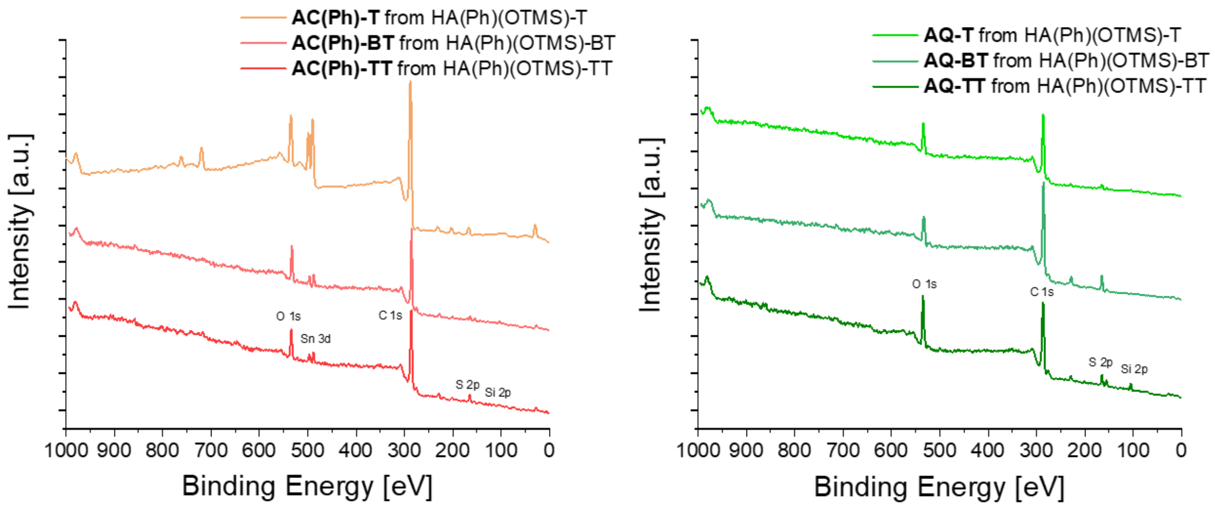

Figure S15. XPS wide scans of conjugated polymers obtained from HA-precursors. The main XPS peaks for the elements observed are labelled in the panels. Percentage values are reported in Table S2 and compared to theoretical values.

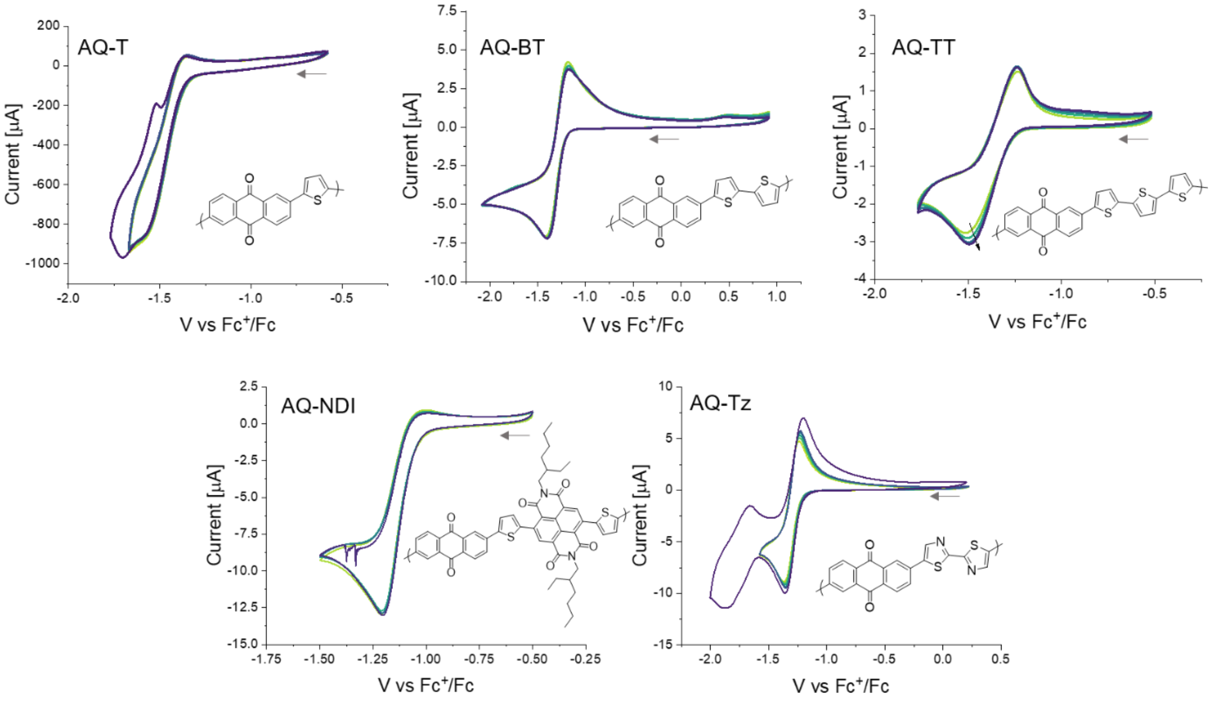

Figure S16. Voltammograms (5 scans; from light green to blue) of different films of AQ containing polymers on Au in Bu_4NPF_6 0.1 M acetonitrile solution.

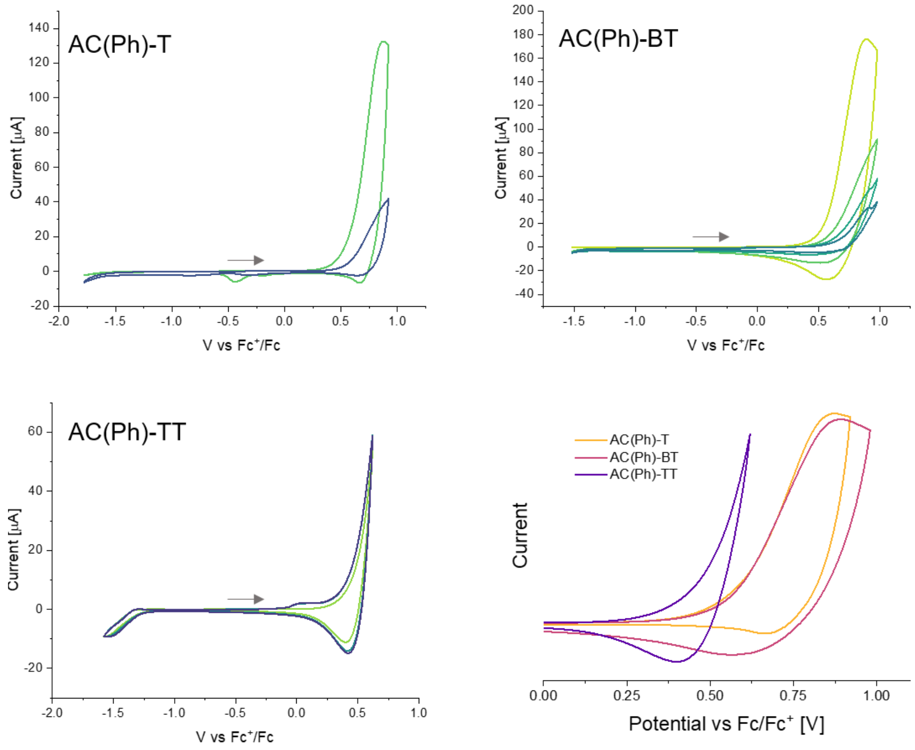

Figure S17. Voltammograms (5 scans; from light green to blue) of different films of AC containing polymers on Au in Bu_4NPF_6 0.1 M acetonitrile solution. In the bottom right corner, we reported a comparison of the 1st CV scan for all the polymers.

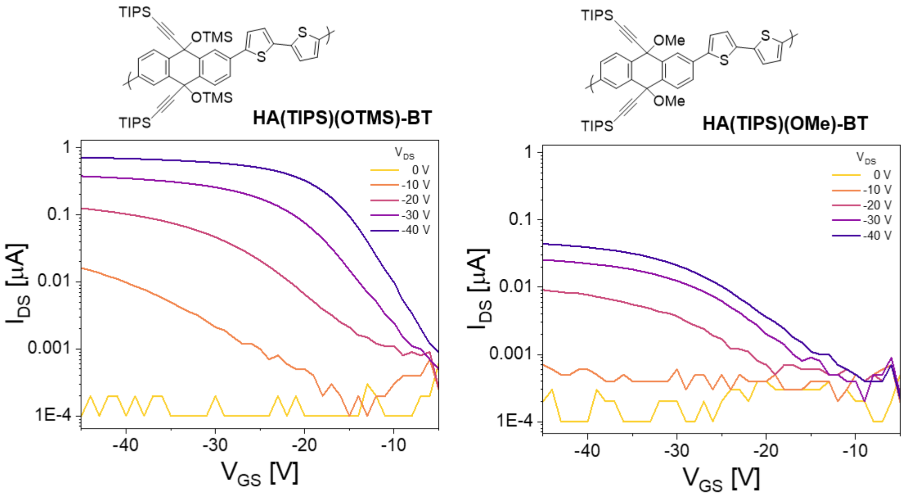

Figure S18. Transfer curves of OFET devices, operated in p-mode, comprising **AC(TIPS)-BT** obtained from **HA(TIPS)(OTMS)-BT** (left) and **HA(TIPS)(OMe)-BT** (right).

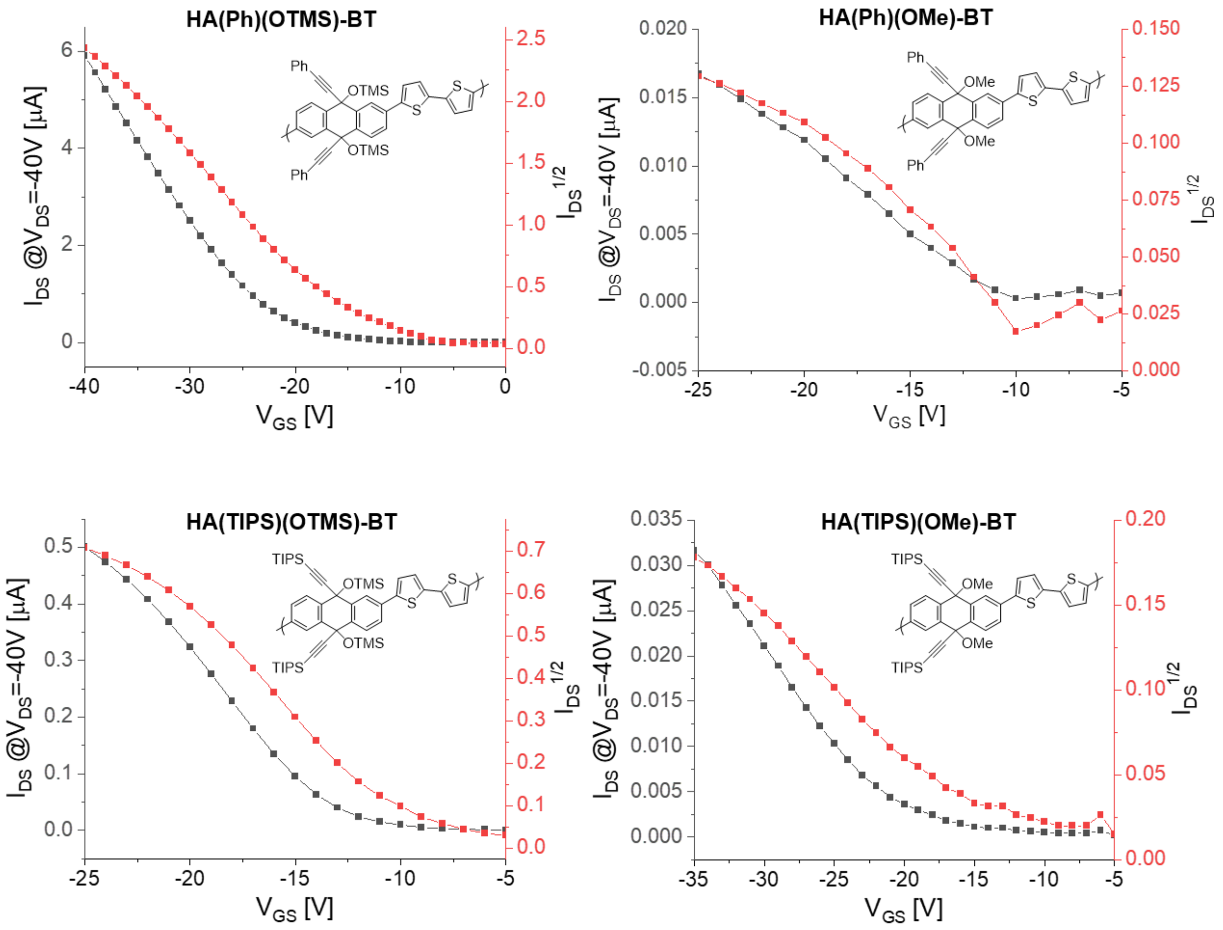

Figure S19. Particulars of the V_{DS} = -40 V transfer curves of **AC(TIPS)-BT** and **AC(Ph)-BT** obtained from different precursors (insets).

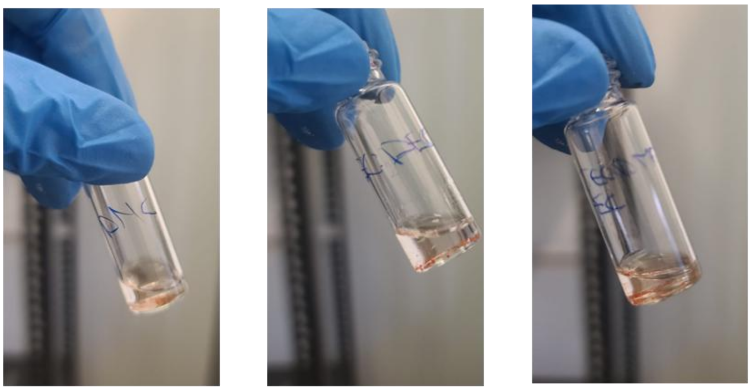

DMC EC:DEC TEGDME:EC

Figure S20. Solubility test of oAQ-T in DMC, EC:DEC 1:1, and TEGDME:EC 1:1 after stirring.

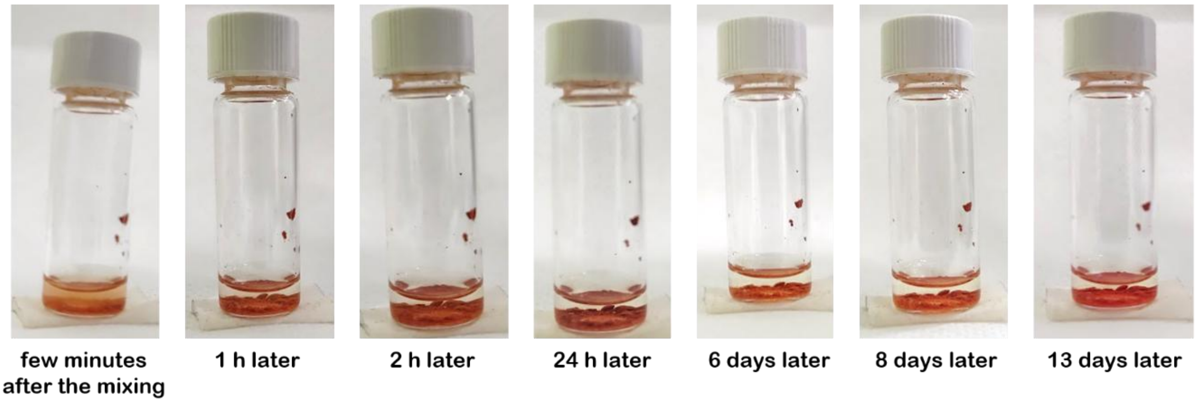

Figure S21. Solubility test of oAQ-T in DME solvent throughout 13 days.

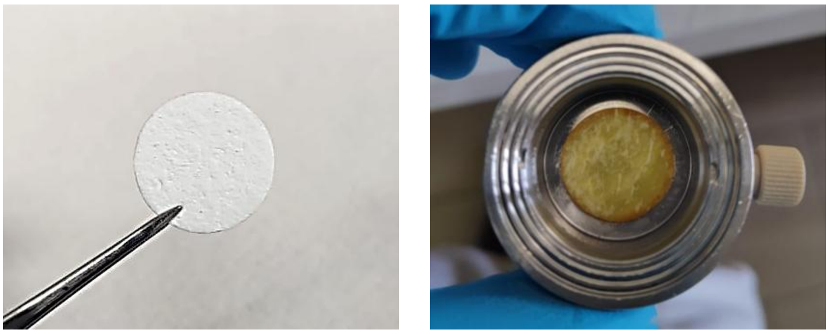

Figure S22. Pictures of Whatman glass fiber separator before (left) and after (right) cycling using **oAQ-T** electrode as cathode material in KIBs.

References

[S1] K. Oniwa, H. Kikuchi, T. Kanagasekaran, H. Shimotani, S. Ikeda, N. Asao, Y. Yamamoto, K. Tanigaki, T. Jin, *Chem Commun* **2016**, *52*, 4926.

[S2] J. Han, A. Chiu, C. Ganley, P. McGuiggan, S. M. Thon, P. Clancy, H. E. Katz, *Angew. Chem. Int. Ed.* **2021**, *60*, 27212.

[S3] M. Carlotti, T. Losi, F. De Boni, F. M. Vivaldi, E. Araya-Hermosilla, M. Prato, A. Pucci, M. Caironi, V. Mattoli, *Polym Chem* **2023**, *14*, 4465.

[S4] A. Giovanelli, M. Carlotti, *J Fluor Chem* **2025**, *283–284*, 110405.
